# Tilting and Tumbling in Transmembrane Anion Carriers: Activity Tuning through *n*‐Alkyl Substitution

**DOI:** 10.1002/chem.201504057

**Published:** 2016-01-08

**Authors:** Sophie J. Edwards, Igor Marques, Christopher M. Dias, Robert A. Tromans, Nicholas R. Lees, Vítor Félix, Hennie Valkenier, Anthony P. Davis

**Affiliations:** ^1^ School of ChemistryUniversity of BristolCantock's CloseBristolBS8 1TSUK; ^2^ Departamento de QuímicaiBiMED and CICECOUniversidade de Aveiro3810-193AveiroPortugal

**Keywords:** anion transport, lipophilicity, membranes, molecular dynamics, supramolecular chemistry

## Abstract

Anion transport by synthetic carriers (anionophores) holds promise for medical applications, especially the treatment of cystic fibrosis. Among the factors which determine carrier activity, the size and disposition of alkyl groups is proving remarkably important. Herein we describe a series of dithioureidodecalin anionophores, in which alkyl substituents on one face are varied from C_0_ to C_10_ in two‐carbon steps. Activities increase then decrease as the chain length grows, peaking quite sharply at C_6_. Molecular dynamics simulations showed the transporter chloride complexes releasing chloride as they approach the membrane‐aqueous interface. The free transporter then stays at the interface, adopting an orientation that depends on the alkyl substituent. If chloride release is prevented, the complex is positioned similarly. Longer chains tilt the binding site away from the interface, potentially freeing the transporter or complex to move through the membrane. However, chains which are too long can also slow transport by inhibiting movement, and especially reorientation, within the phospholipid bilayer.

## Introduction

The transport of anions across phospholipid bilayers has become an active field of supramolecular chemistry.^1^ There is particular interest in developing small molecules for use in biological research and medicine. For example, a number of genetic conditions are caused by malfunctioning chloride channels. These include Bartter syndrome, Best disease and, most importantly, cystic fibrosis.^2^ Synthetic anion transporters could potentially be used to treat such conditions by replacing the activity of the missing or malfunctioning chloride channels.^3^

The activities of synthetic anion carriers are determined by various factors. Early work is focused on anion affinities, and there are many examples of series within which transport rates correlate with binding constants.^4^ However, these correlations do not extend beyond closely related molecules, and it is clear that other considerations are relevant.^5^, ^6^ Interactions of receptors and complexes with the membrane environment are especially significant. On one level, it is clearly important that transporters must partition into the membrane from water, and that if added to pre‐existing membranes (e.g., cells or aqueous vesicle suspensions) they must be able to find the interiors of the bilayers. Molecules which are too hydrophilic may fail on the first count, whereas molecules which are too hydrophobic may fail on the second (due to precipitation from water). These factors may underlie the observation of optimum lipophilicity in studies of transporters such as tambjamines **1**,^7^ phenylthioureas **2** 8, 9 and acylthioureas **3**.^10^ 
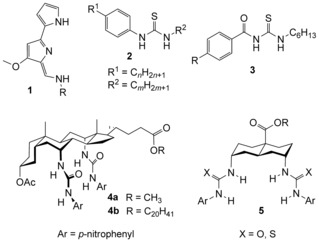


However, other investigations show that lipophilic groups can also affect the intrinsic transport activities of anionophores, that is, the rate at which they operate once present in the membranes. To study these effects, it is necessary to use assay methods in which the transporter concentration within the membranes is known reliably. In practice, this can be achieved by: 1) employing transporters which are too lipophilic to leach into the aqueous phase; and 2) ensuring that these molecules are in the membranes by pre‐incorporating them during vesicle production. Implementation of such methodology is non‐trivial, because the presence of anionophores in the membranes means that the vesicles are anion permeable at all stages of the experiment. However, the problem has been solved by the lucigenin method^11^ as discussed later. This technique has yielded some interesting results when applied to anion carriers with variable lipophilic substituents. In some cases, effects are small. Thus, for the steroid‐based “cholapods”, such as **4**, the methyl and eicosyl esters **4 a** and **b** possess very similar activities.4b However, for other systems, the effects can be large. For example, in bis‐(thio)ureidodecalins **5**, extending side‐chain R gave substantial benefits (e.g., a four‐fold increase in activity for R=methyl→octyl^12^). Moreover, these effects do not just depend on the overall lipophilicity of the transporters. In a collaboration with the group of Gale, we have studied a series of simple thioureas **2** for which *n*+*m*=11.^13^ These molecules are equally lipophilic, but their transport activities were found to vary by a factor of four. Performance was optimal when the side‐chains were roughly equal in length (e.g., *n*=5, *m*=6), suggesting that “lipophilic balance” is advantageous for anionophore activity.

The variations observed within **2** and **5** are presumably related to the way that the anionophores and/or their anion complexes interact with the membranes. Tuning these interactions to promote transport will play a significant role in anionophore development, and it is therefore important to develop an understanding of the phenomena involved. Herein, we report a study on a new set of alkyl‐substituted transporters **7**. Similar to **2**, these molecules provide continuous structural variation over a range of chain lengths, giving fine‐grained information on lipophilic substituent effects. At the same time, they belong to the family of bis‐(thio)ureidodecalins **5**, which are more relevant to potential applications; members include the most powerful anionophores reported to date,4g and have shown encouraging responses in cell‐based assays.^14^ We find that activities are remarkably sensitive to the alkyl substituent, passing through a maximum as the chain length increases. The results are interpreted with the help of extensive molecular dynamics (MD) calculations,^15^ which suggest explanations for both increases and decreases of transport rates along the series. The study provides further evidence that anion carriers are strongly affected by their interaction with the membrane environment, and that these interactions are surprisingly sensitive to subtle changes in apolar substitution.

## Results and Discussion

### Synthesis

**Scheme 1:**
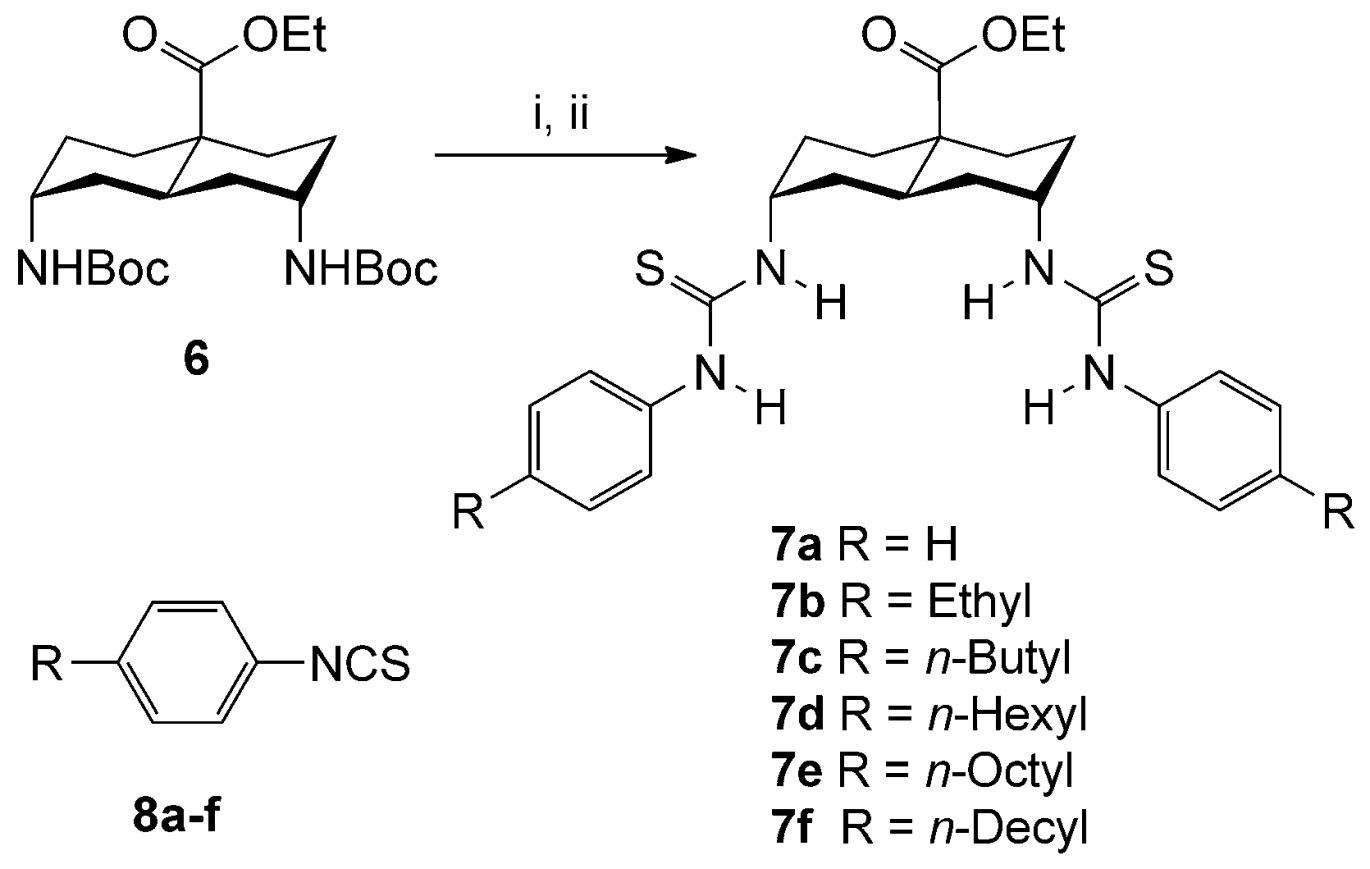
(i) Trifluoroacetic acid (TFA), CH_2_Cl_2_, rt, 16 h; (ii) isothiocyanate **8 a**–**f** (2.2 equiv), 4‐dimethylaminopyridine (DMAP; 2.7 equiv), diisopropylethylamine (DIPEA), THF, rt, 16 h.

Transporters **7 a**–**f** were synthesised in two steps from Boc‐protected diamine **6** (see Scheme 1), which was prepared following a published route.^12^ The isothiocyanates **8 a**–**f** required to synthesise thioureas **7 a**–**f** are commercially available with the exception of 4‐hexylphenyl isothiocyanate **8 d** and 4‐octylphenyl isothiocyanate **8 e**, which were made from the corresponding anilines (see ESI for details).^16^ The Boc‐protected diamine **6** was deprotected and treated with the appropriate isothiocyanate **8 a‐f** and base. Dithioureido decalins **7 a**–**f** were isolated in yields of 90 %, 94 %, 80 %, 68 %, 48 % and 47 % respectively.

### Anion binding

The binding affinities of dithioureido decalins **7 a**–**f** to Et_4_N^+^Cl^−^ in chloroform were measured using a modification of Cram's extraction method.^17^ Affinities to Bu_4_N^+^Cl^−^ in [D_6_]DMSO/H_2_O (200:1) were also obtained by using ^1^H NMR titration. The results are listed in Table 1. The binding constants in wet DMSO were roughly three orders of magnitude lower than those in chloroform, reflecting the different polarities of the solvents. The slightly higher *K*_a_ value measured for the phenyl thiourea **7 a** in chloroform can be explained by the increased acidity of its NH groups relative to those of compounds **7 b**–**f**, which are moderated by the electron‐rich nature of their alkyl substituents. However, overall there was no significant or constant variation between the *K*_a_ values across the series. Therefore, it can be assumed that any variation in transport activity is unrelated to binding affinities.

**Table 1:** Binding affinities and transport data for dithioureido decalins **7 a**–**f**.

	R	*c* log *P* ^[a]^	*K*_a_ [m ^−1^]		Initial rate [s^−1^]^[d]^		[*I*]
			CHCl_3_^[b]^	[D_6_]DMSO^[c]^	1:2 500	1:1 000	[s^−1^]^[e]^
**7 a**	H	5.2	4.3×10^5^	7.3×10^2^	0.0010	0.0026	2.6
**7 b**	Et	6.4	1.6×10^5^	7.0×10^2^	0.0012	0.0033	3.2
**7 c**	Bu	7.9	1.6×10^5^	7.2×10^2^	0.0027	0.0043	5.5
**7 d**	Hex	9.5	1.5×10^5^	7.2×10^2^	0.0031	0.0068	7.3
**7 e**	Oct	11.0	1.7×10^5^	7.1×10^2^	0.0018	0.0034	4.0
**7 f**	Dec	12.6	1.8×10^5^	6.7×10^2^	0.0015	0.0024	3.1

[a] *c* log *P* values were calculated with torchV10 Lite (available as freeware from Cresset BMD, see http://www.cresset‐group.com). [b] Obtained by extraction of Et_4_N^+^Cl^−^ from water into chloroform at 303 K, as described in ref. 17c. [c] Obtained from ^1^H NMR titrations with Bu_4_N^+^Cl^−^ in [D_6_]DMSO/H_2_O (200:1) at 298 K. [d] Transporter/lipid 1:2 500 or 1:1 000. Obtained from fits (0–500 s) of *F*_0_/*F* to a double exponential function. [e] Specific initial rate [*I*]: Initial slope of *F*_0_ */F* versus time *t*, divided by the transporter/lipid ratio in the vesicle bilayers and averaged over a range of experiments at different ratios.4g

### Anion transport measurements

Dithioureido decalins **7 a**–**f** are hydrophobic compounds, designed to partition exclusively into vesicle membranes. This means that the transporter/lipid ratio can be known exactly once the molecules are in place, but also that delivery to preformed vesicles is problematic (see earlier discussion). Therefore, it is necessary that the transporters should be premixed with membrane lipids before vesicle preparation. If the transporters are already present at the beginning of an assay, the experiment must be started by adding the target anion (as opposed to transporter) to the external solution. This means that anion efflux cannot be followed, so that analytical methods, which sample the external solution (e.g., anion‐selective electrodes4a, 18) cannot be used. On the other hand, it is possible to monitor chloride influx by enclosing a halide‐sensitive fluorescent dye (e.g., lucigenin) in the vesicles,^11^ and it is this method which was used in the present work.

**Figure 1:**
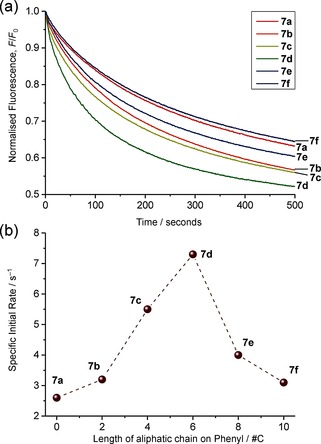
(a) Chloride transport into vesicles (POPC/cholesterol 7:3) facilitated by decalins **7 a** (red), **7 b** (orange), **7 c** (yellow), **7 d** (green), **7 e** (blue), and **7 f** (purple) at a 1:1000 transporter to lipid ratio. (b) Plot of the specific initial rate values of chloride transport by decalins **7 a**–**f** as a function of the length of the aliphatic chain R. Activities peak for **7 d**.

Large unilamellar vesicles (LUVs, 0.4 mm, 200 nm average diameter) consisting of 1‐palmitoyl‐2‐oleoylphosphatidyl‐choline (POPC) and cholesterol in a 7:3 ratio were employed, and the decalins were pre‐incorporated into the membrane at transporter/lipid ratio of 1:2 500 and 1:1 000. The vesicles were prepared with internal and external aqueous NaNO_3_ (225 mm) and internal lucigenin (0.8 mm). The vesicle solutions were placed in a fluorescence spectrometer, and an external pulse of sodium chloride (25 mm) was added. The influx of Cl^−^ was monitored through the decay in lucigenin's fluorescence, which was analysed to give initial rates (which are concentration dependent) and specific initial rates [*I*]4g (independent of concentration of transporter) for chloride–nitrate exchange. Further details of the experimental and analysis methods are given in the Supporting Information. The fluorescence decay traces are shown in Figure 1 a, the initial rates and specific initial rates are summarised in Table 1, and the trend in specific initial rate is visualised in Figure 1 b. To ensure that the transporters were located exclusively in membranes, so that variations reflected intrinsic transport ability, a “leaching test”^12^, ^13^ was performed for the least lipophilic variant **7 a**. Briefly, the transporter was incorporated in vesicles in the normal fashion, then aliquots of the vesicle suspension were either diluted or passed through a size‐exclusion column. If the transporter is partitioning between lipid and aqueous phases, both procedures should result in loss of transporter molecules from the bilayers. Subsequent transport assays showed no sign of transporter loss, implying that **7 a** is confined to the membranes. Further details are given in the Supporting Information.

As was expected, the bis‐thioureas **7 a**–**f** were found to be moderately effective as anion transporters, less active than analogues with electron‐deficient aryl groups,4g but more efficient than the simple thioureas **2**. As shown in Figure 1 b, adjusting groups R produces significant effects. On increasing the length from C_0_ to C_6_, transport rates increase by a factor of approximately 3. However, further increases to C_10_ then lower the rates by almost the same amount. Thus, activity passes through a maximum, in a fashion reminiscent of the results for **2**.^13^ This trend was observed at transporter to lipid ratios of both 1:1 000 and 1:2 500 (see the Supporting Information). The observation of a maximum in Figure 1 b suggested a subtle role for groups R in determining activity, probably involving the details of interactions with membrane lipids. To explore this possibility further, we turned to computational methods, as discussed below.

### MD insights into position, orientation and movement within the bilayer

To assess how the lipophilic groups affect movement and positioning within the membrane, we carried out extensive molecular dynamics (MD) simulations at the atomistic level with **7 a**–**f** in a POPC bilayer. The starting scenarios for the MD simulations had the chloride complexes of the transporters placed at the centre of a phospholipid bilayer and randomly orientated.^19^ The simulations showed the complexes moving towards the surface of the bilayer, followed by chloride release and further motions of the free receptors. The MD simulations were performed using the AMBER software suite^20^ with the thiourea transporters described with atomic charges obtained by multi‐conformational RESP^21^ fitting, and force‐field parameters taken from GAFF.^22^ Force‐field parameters from Lipid14^23^ were used for the POPC phospholipids. The bilayer was composed of 128 POPC phospholipids and 6500 TIP3P water molecules.^24^ 18 sodium and 18 chloride ions, described with suitable parameters,^25^ were also present in the membrane model. The computational details are given in the Supporting Information together with a thorough description of all simulation protocols.

The structures of chloride‐thiourea complexes, depicted in Figure S27 in the Supporting Information, were obtained in the gas phase by quenched molecular dynamics as detailed in the Supporting Information. Afterwards, each chloride–thiourea complex was initially positioned at the core of the POPC bilayer (setup A) and subjected to a multi‐stage MD equilibration protocol with a positional restraint on the complex. The restraint was then removed, and the complex was allowed to diffuse freely for 150 ns. Four independent replicates were carried out, leading to a total sampling of 600 ns for each system. These MD runs are summarised in Table S3 in the Supporting Information, along with the other setups A′ and B used in this theoretical investigation (see below). Each simulation ID derives from the combination of the initial position of the transporter in the phospholipid bilayer, the replicate number and the transporter. For instance, A_1_.**7 a** denotes the first replicate carried out with **7 a** in setup A. A preliminary analysis of all MD simulation runs revealed that the presence of the chloride complexes in the POPC membrane system has a negligible impact on the structural parameters of the bilayer (area per lipid, bilayer thickness, electron‐density profiles and order parameters) compared with a pure membrane system, as further detailed in the Supporting Information (Section 4.2).

**Figure 2:**
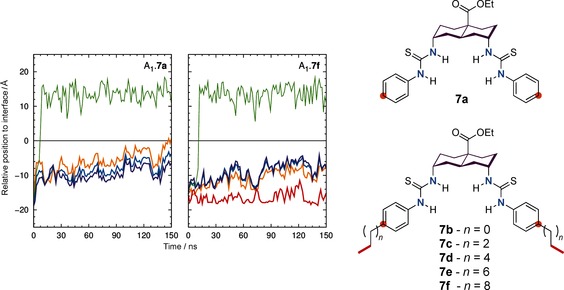
Evolution of P_int_ **⋅⋅⋅**decalin_COM_ (purple), P_int_ **⋅⋅⋅**N−H_COM_ (blue), P_int_ **⋅⋅⋅** *p*‐C_COM_ (orange) and P_int_ **⋅⋅⋅**tail_COM_ (red) distances for A_1_.**7 a** (left plot) and A_1_.**7 f** (right plot), as well as the corresponding P_int_ **⋅⋅⋅**Cl^−^ (light green) during 150 ns of simulation time. The water/lipid interface is represented as a black line at *z*=0 Å. The atoms used to define each centre of mass are identified in the far right sketch with purple, blue, orange, and red for decalin_COM_, N−H_COM_, *p*‐C_COM_, and tail_COM_, respectively, in agreement with the corresponding lines in the plots.

The relative position of the chloride towards the closest membrane interface was evaluated throughout the course of the MD runs using the P_int_ **⋅⋅⋅**Cl^−^ distance, in which P_int_ represents the centre of mass determined by the phosphorus atoms in that monolayer (which we consider as the water/lipid interface). Likewise, the position of the transporter was evaluated by the distance between P_int_ and centre of mass (COM) defined by the carbon atoms of the decalin skeleton (P_int_ **⋅⋅⋅**decalin_COM_). The P_int_ **⋅⋅⋅**N−H_COM_, P_int_ **⋅⋅⋅** *p*‐C_COM_, P_int_ **⋅⋅⋅**tail_COM_ distances were also determined, in which N−H_COM_, *p*‐C_COM_ and tail_COM_ represent the centres of mass determined by the nitrogen atoms of the two thiourea binding sites, the phenylene carbon atoms *para* to the thiourea groups, and the two terminal carbon atoms in each of the aliphatic groups R in **7 b**–**f**, respectively. All these reference points are identified in the sketch given in Figure 2, with colour coding. Taken together, the P_int_ **⋅⋅⋅**decalin_COM_, P_int_ **⋅⋅⋅**N−H_COM_ and P_int_ **⋅⋅⋅** *p*‐C_COM_ distances give the position and the orientation of the receptor core relative to the membrane interface. Meanwhile, the P_int_ **⋅⋅⋅**tail_COM_ distance provides information on the extension and orientation of the alkyl substituents.

**Figure 3:**
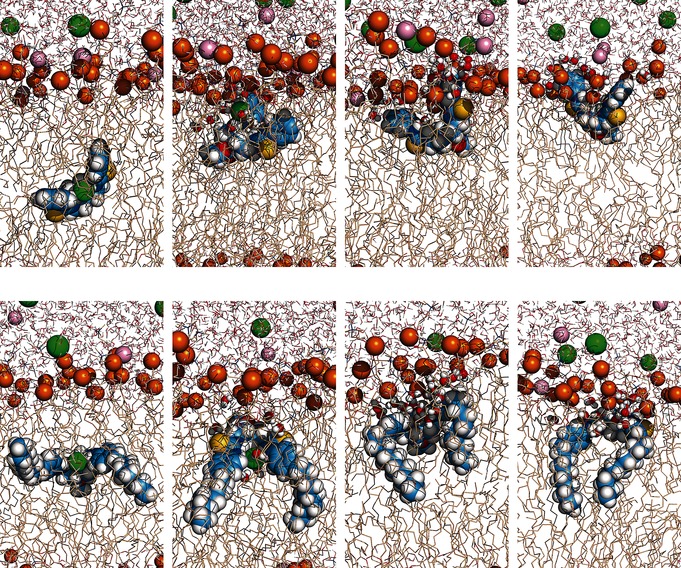
Consecutive snapshots depicting the diffusion of the chloride complex of **7 a** and **7 f** in simulations A_1_.**7 a** (top) and A_1_.**7 f** (bottom). The transporter, the phosphorus atoms and ions are represented as spheres. The hydrogen atoms are shown in white, oxygen atoms in red, nitrogen atoms in blue, sulfur atoms in yellow, phosphorus atoms in orange and carbon atoms in light blue (transporter) or wheat (phospholipids), whereas the chloride and sodium ions are shown in green and pink, respectively. The chloride decomplexation assisted by water is emphasized with the depiction of water molecules within 3.5 Å from **7 a** or **f** as spheres. The lipid C−H bonds are omitted for clarity.

The evolution of these four distances in simulations A_1_.**7 a** and A_1_.**7 f** are shown in Figure 2 (left and right plots, respectively), whereas the full set of data is represented similarly in Figures S40–S45 in the Supporting Information. In all cases, chloride release occurs within the first 15 ns of the 150 ns simulation. Snapshots of the simulations (Figures 3 and S46, S47 in the Supporting Information) revealed that when the complex moves towards the water/lipid interface, it is met by water molecules permeating from the aqueous phase. The water molecules solvate the chloride anion and promote the breakup of the complex, so that chloride release occurs before the complex reaches the membrane interface. The diffusion of the chloride complex of **7 d** in simulation A_1_.**7 d** is featured in Movie S1 in the Supporting Information, which also presents the anion release assisted by water molecules. The time taken for chloride release varies between runs, and does not show meaningful differences between receptors.

Once the chloride ions have departed, the simulations showed the transporter molecules continuing their movement towards the interface and settling into equilibrium positions. In these later parts of the simulations, consistent differences emerge between **7 a**–**f**. In the case of **7 a**, the transporter adopts an orientation in which the aromatic rings (Figure 2 left, orange line) are clearly closer to the interface than the decalin moiety and thiourea‐binding units (purple and blue lines, respectively). This preferential orientation, with the phenyl groups angled towards the interface, is also evident in the last two consecutive snapshots presented in Figure 3 (top). In contrast, the medium‐sized transporters **7 b** and **c** (R=ethyl, *n*‐butyl) do not display a clear preferential orientation, whereas the largest transporters **7 d**–**f** (R=*n*‐hexyl, *n*‐octyl, *n*‐decyl) are positioned with the aromatic groups angled away from the interface (see Figures 2 (right plot), S45 in the Supporting Information and Figure 3 (bottom) for **7 f**, as well as Figures S43, S44, and S47 for **7 d** and **e**).

**Figure 4:**
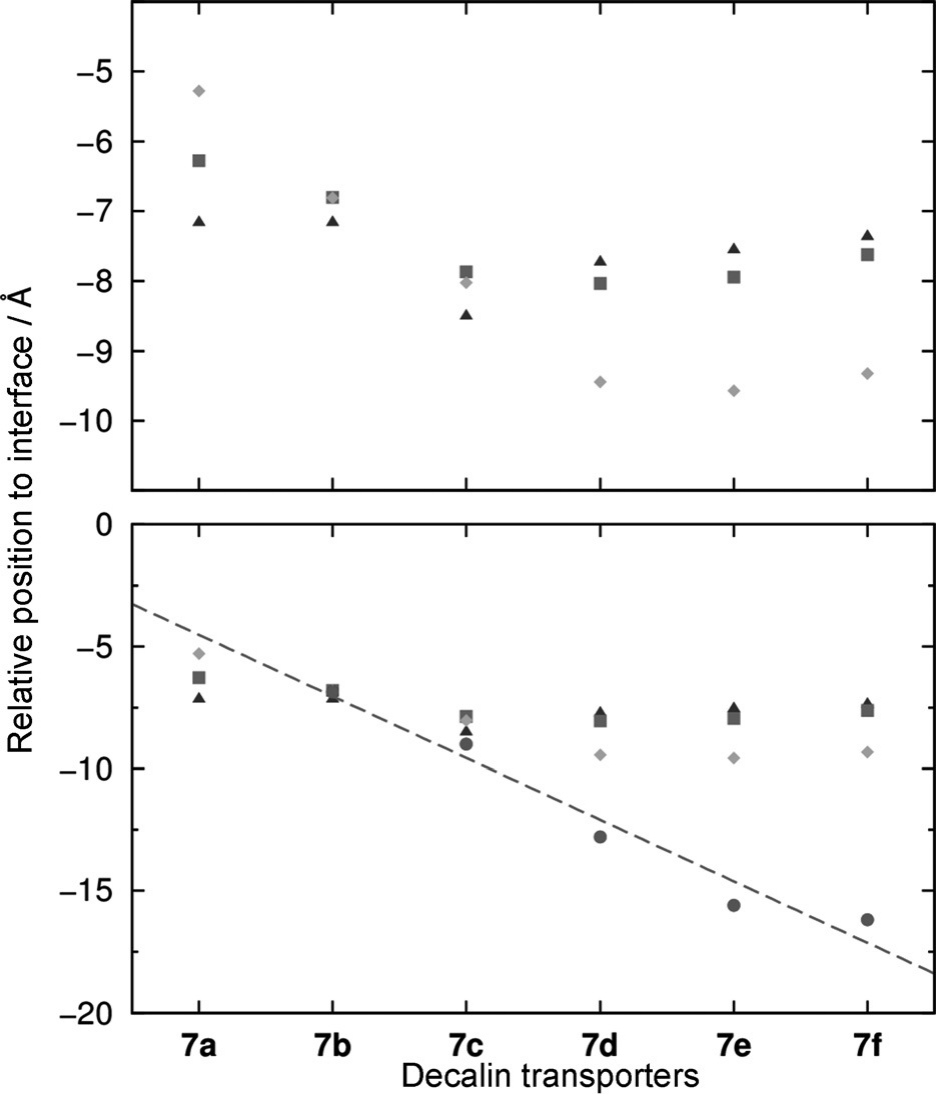
Average positions of decalin_COM_ (▴), N−H_COM_ (▪), *p*‐C_COM_ (⧫), and tail_COM_ (•) thiourea reference points relative to the closest interface. Each point was calculated averaging the last 50 ns of the four independent setup A MD simulation replicates, resulting in 200 ns of total sampling time.

The differences in orientation are highlighted in Figure 4, which shows the average position of the reference points (decalin_COM_, N−H_COM_, *p*‐C_COM_, and tail_COM_) over the final 50 ns of each simulation (including replicates). For compound **7 a**, the reference points *p*‐C_COM_ (⧫), N−H_COM_ (▪), and decalin_COM_ (▴) lie at increasing distances from the interface. For compounds **7 b** and **c**, they are close to each other, and for **7 d**–**f**, they appear in the reverse order.

When the simulations were performed with the chloride–thiourea complexes positioned initially in the water phase (setup B), the chloride was also released quickly, after which the thioureas entered the membrane. Their final positions and orientations (see Figure S48 in the Supporting Information) resemble those found when the bilayer core was the starting position, indicating that our results are independent of the initial setup of the experiments.

To assess the effect of chloride binding on transporter orientation, a single MD run for each complex was also undertaken with four distance restraints between the chloride and the nitrogen atoms of the thiourea binding units, as described in the Supporting Information (setup A′). The six chloride complexes migrate from the bilayer core to the interface, staying at P_int_ **⋅⋅⋅**decalin_COM_ distances (see Figure S48 in the Supporting Information) comparable to those calculated for setup A. The average P_int_ **⋅⋅⋅**N−H_COM_, P_int_ **⋅⋅⋅** *p*‐C_COM_ and P_int_ **⋅⋅⋅**tail_COM_ distances are also comparable in both setups suggesting that the preferred orientations for complex and transporter are similar in each case.

The change of orientation on passing from **7 a** through to **7 d** suggests an explanation for the increase in activity along this sequence (Figure 1 b, first four data points). For compound **7 a**, both transporter and chloride complex lie with their polar regions directed towards the membrane interface. They are thus able to form effective polar interactions, either with the phospholipid head groups (in the case of **7 a** itself) or with water molecules that are bound to bulk aqueous phase (for both **7 a** and **7 a**⋅Cl^−^). These polar interactions must be broken before the transporter/complex can pass through the membrane, and therefore represent potential barriers to transport. When hydrocarbon chains are added to the *para* positions of the phenyl groups, they tend to turn the transporter/complex, pulling the polar region away from the interface. The interactions with the interface are now easier to break, so that transport is facilitated. Although this logic can be applied to both transporter and chloride complex, the movement of the complex is probably more relevant, because this likely serves as the rate‐determining step for the overall transport process. A related argument was previously used to explain why “lipophilic balance” favours transport in thioureas **2**.^13^ In that case, a polar unit at one end of a chain was expected to interact especially well with the membrane interface (although, unlike the present work, the hypothesis was not supported by calculations).

The decrease in activity from **7 d** to **f** (Figure 1 b, last three data points) does not seem to result from the positioning of the receptors or complexes in the membranes. As shown in Figure 4, the distance parameters for *p*‐C_COM_ (⧫), N−H_COM_ (▪), and decalin_COM_ (▴) remain fairly constant along the series. However, it is notable that the P_int_ **⋅⋅⋅**tail_COM_ distance (•) increases steadily from **7 b** through to **7 f**, implying that the hydrocarbon tails are extended into the membrane interior, generally aligned with the phospholipids (see also Figure 3, bottom right). Viewing the geometry of the transporter/complex at the interface, it is apparent that a 180° rotation must occur at some point to allow complex formation or chloride release at the opposite interface. Such inversions were observed during the simulations, but only occasionally and much more often for transporters with shorter side‐chains (especially, **7 a**–**c**, R=H, Et, Bu). Although the alkyl side‐chains are flexible, they will tend to pack with the phospholipid in a manner that maximises van der Waals interactions (as was evident from the molecular mechanics energetic analysis presented in the Supporting Information, Section 4.4), providing a significant length‐dependent barrier to rotation. Although the calculations focus on the free receptors, it is likely that the chloride complexes would behave similarly. When the side‐chain increases in length, it is possible that reorientation of the receptor or (more likely) complex could become rate‐determining, accounting for the trend from **7 d** to **f**. The increase in chain length could also hinder other types of movement, but the reorientation would appear to be especially critical.

## Conclusion

**Figure 5:**
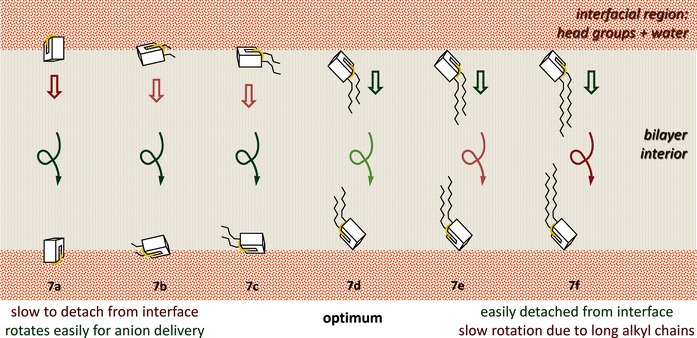
Rationalisation of transport data for **7 a**–**f**, as was suggested by molecular dynamic simulations. Cartoons depict chloride complexes of the six transporters. All complexes locate at the membrane interface, but orientations are different. For **7 a**, the binding site is directed towards the aqueous phase. This allows the bound anion and polar groups to interact effectively with water molecules, which are linked by hydrogen bonding to bulk water. These interactions are relatively difficult to break, inhibiting transport. When alkyl chains are added to the receptor, the complex is turned by hydrophobic interactions. The connection to the aqueous phase becomes weaker, and more easily broken. Transport rates reach a maximum at **7 d**, after which reorientation of the complex in the membrane becomes rate determining. This process is especially important for longer chains, which obstruct release of chloride if pointing in the wrong direction, and also becomes slower as chain lengths increase, in agreement with our molecular mechanics energetic analysis.

We have shown that the intrinsic activity of *trans*‐decalin anion carriers **7** is remarkably sensitive to alkyl substitution. When groups R increase in length, conflicting effects seem to operate, first increasing then decreasing transport activities. The result is a sharp maximum for anion transport rates at R=hexyl. Molecular dynamics calculations have provided detailed information on the movements and positions of the transporters in the membranes. The results point to explanations for both effects, as summarised in Figure 5. For short R, the polar regions of the carriers/complexes are angled towards the membrane‐aqueous interface, in which they can interact with water or phosphate groups, hindering transport. For long R, the transporter/complex finds difficulty reorienting within the membrane when it passes between interfaces. Maximum activity occurs when the alkyl chains are long enough to help the transporter/complex escape the interface, but not so long as to hinder rotation. These interpretations are suggestive at present, but if confirmed by future studies, they will provide useful insight into anionophore design. Meanwhile, the molecular dynamics results suggest that further simulations employing improved force‐field parameters and complementary methodologies, including free‐energy calculations from suitable MD simulation approaches, could lead to a full understanding of anion transport in phospholipid membranes. This theoretical investigation is currently in progress.

## Supporting information

As a service to our authors and readers, this journal provides supporting information supplied by the authors. Such materials are peer reviewed and may be re‐organized for online delivery, but are not copy‐edited or typeset. Technical support issues arising from supporting information (other than missing files) should be addressed to the authors.

Supplementary

Supplementary
