# Supplementary material for: Tilting and Tumbling in Transmembrane Anion Carriers: Activity Tuning through n‐Alkyl Substitution
Source: Chemistry. 2016 Jan 8;22(6):2004–11. doi: 10.1002/chem.201504057 (PMC5064602; doi:10.1002/chem.201504057)
Supplement: Supplementary file 1 — Supplementary [file CHEM-22-2004-s001.pdf]

CHEMISTRY
A European Journal

Supporting Information

Tilting and Tumbling in Transmembrane Anion Carriers: Activity Tuning through *n*-Alkyl Substitution

Sophie J. Edwards,[a] Igor Marques,[b] Christopher M. Dias,[a] Robert A. Tromans,[a] Nicholas R. Lees,[a] Vítor Félix,*[b] Hennie Valkenier,*[a] and Anthony P. Davis*[a]

chem_201504057_sm_miscellaneous_information.pdf
chem_201504057_sm_SI.mp4

Contents

1. Synthesis and Characterisation 3
   1.1 General methods 3
   1.2 Synthesis of isothiocyanates **8d** and **8e** 4
   1.3 General Procedure I for synthesis of decalin transporters **7a-f** 5
   1.4 NMR Spectra of **7a-f** 10
2. Anion Binding Affinities 16
   2.1 Chloride binding affinities by ^{1}H NMR titration in DMSO-d_6 16
   2.2 Chloride binding affinities in chloroform by Cram's extraction method 23
3. Transport Studies 25
   3.1 General experimental description of transport measurements 25
   3.2 Fitting of transport data 27
   3.3 Test for leaching of decalin **7a** from vesicle membranes 28
4. Molecular Dynamics Simulations 29
   4.1 Computational Methods 29
   4.2 Structural impact of **7a-f** on the bilayer model 33
   4.3 Detailed Results of the Molecular Dynamics Simulations 41
   4.4 Interaction energies between transporters **7a-f** and POPC bilayer 50
   4.5 Supplementary MD Movie Caption 53
5. References 54

1. Synthesis and Characterisation

The six new decalin transporters described in this communication were synthesised as detailed below. The Boc-protected diamine **6** was prepared as previously described.[1] The required isothiocyanates were obtained commercially, with exception of 4-hexylphenylisothiocyanate **8d** and 4-octylphenylisothiocyanate **8e** which are not commercially available, and were prepared as described below.[2]

1.1 General methods

All reagents were purchased from commercial suppliers and used without further purification, unless otherwise stated. Anhydrous DCM and THF were dried by passing through a modified Grubbs system, with an alumina column manufactured by Anhydrous Engineering. Flash column chromatography was performed using silica gel (Aldrich, pore size 60 Å, particle size 40-63 µm) as the absorbent. Routine monitoring of reactions was performed using precoated silica gel TLC plates (Merck silica gel 60 F_{254}). Spots were visualised under UV light or by staining with phosphomolybdic acid, potassium permanganate or ninhydrin; R_f values are given under these conditions.

^{1}H, ^{13}C and ^{19}F NMR spectra were recorded using a ECS 300, ECS 400, Varian 400, Varian 500A (carbon sensitive) or Varian 500B (proton sensitive) spectrometer. All spectra are recorded at 298 K unless otherwise stated. Chemical shifts (δ) are quoted in parts per million (ppm), coupling constants (*J*) are quoted in Hz and spectra are referenced to the appropriate residual solvent peak. Mass spectra were recorded on a Bruker microTOF II (ESI), VG Analytical Quattro (ESI) or VG Analytical Autospec (EI). IR spectra were recorded on a Perkin-Elmer Spectrum 100 FT-IR spectrometer. Elemental analysis was carried out by the microanalysis department at the School of Chemistry, University of Bristol.

The carbon numbering system for the decalin-based transporters **7a-f** is as shown below for **7f**:

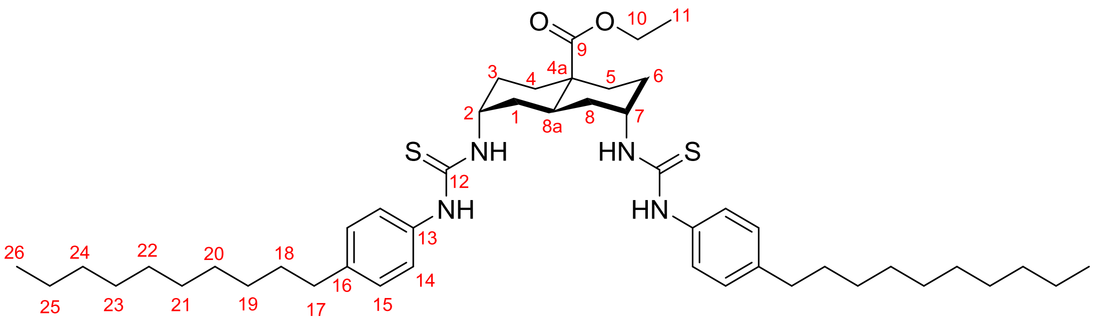

1.2 Synthesis of isothiocyanates 8d and 8e

4-Hexylphenyl isothiocyanate (8d)

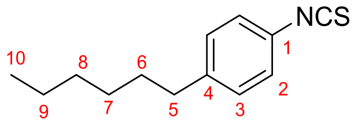

To a solution of 4-hexylaniline (1.0 mL, 5.1 mmol) in absolute EtOH (5 mL) was added carbon disulfide (3.0 mL, 50 mmol) and triethylamine (0.7 mL, 5 mmol). The mixture was stirred at room temperature for 1 h and then cooled to 0 °C using an ice-bath. A solution of di-*t*-butyl dicarbonate (1.10 g, 5.04 mmol) in absolute EtOH (1 mL) was added to the reaction mixture, followed by a solution of DMAP (12 mg, 0.10 mmol) in absolute EtOH (1 mL), and the mixture was stirred at 0 °C for 5 minutes. The mixture was allowed to warm to room temperature and was stirred for 30 minutes after which the solvent was removed *in vacuo* yielding 4-hexylphenyl isothiocyanate **8d** (1.15 g) as a crude brown residue which was used without further purification: R_f 0.8 (50% EtOAc in hexane); ^{1}H NMR (400 MHz, $CDCl_3$) δ 0.93 (3H, t, *J* 6.5, 10-H_3), 1.26-1.44 (6H, m, 7-H_2, 8-H_2 and 9-H_2), 1.57-1.70 (2H, m, 6-H_2), 2.63 (2H, t, *J* 7.8, 5-H_2), 7.09-7.21 (4H, m, 2 × 2-H and 2 × 3-H); ^{13}C NMR (100 MHz, $CDCl_3$) δ 14.2 (10-CH_3), 22.7 (CH_2), 28.9 (CH_2), 31.3 (6-CH_2), 31.7 (CH_2), 35.6 (5-CH_2), 125.6 (2-CH), 128.6 (1-C), 129.5 (3-CH), 134.6 (C=S), 142.6 (4-C); ν_{max} (neat)/cm^{-1} 2957, 2919, 2848, 2088, 1505, 927, 844; HRMS (EI) calc. for $[C_{13}H_{17}NS]^+$ 219.1082, found 219.1088.

4-Octylphenyl isothiocyanate (8e)

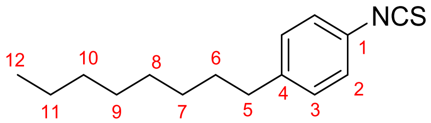

To a solution of 4-octylaniline (1.0 mL, 4.4 mmol) in absolute EtOH (5 mL) was added carbon disulfide (2.7 mL, 45 mmol) and triethylamine (0.6 mL, 4 mmol). The mixture was stirred at room temperature for 1 h and then cooled to 0 °C using an ice-bath. A solution of di-*t*-butyl dicarbonate (0.94 g, 4.3 mmol) in absolute EtOH (1 mL) was added to the reaction mixture, followed by a solution of DMAP (10 mg, 0.082 mmol) in absolute EtOH (1 mL), and the mixture was stirred at 0 ºC for 5 minutes. The mixture was allowed to warm to room temperature and was stirred for 30 minutes after which the solvent was removed *in vacuo* yielding 4-octylphenyl isothiocyanate **8e** (1.13 g) as a crude brown residue, which was used without further purification: R_f 0.9 (50% EtOAc in hexane); ^{1}H NMR (400 MHz, $CDCl_3$) δ 0.93 (3H, t, *J* 6.9, 12-H_3), 1.24-1.40 (10H, m, 7-H_2, 8-H_2, 9-H_2, 10-H_2 and 11-H_2), 1.57-1.67 (2H, m, 6-H_2), 2.63 (2H, t, *J* 7.7, 5-H_2), 7.10-7.22 (4H, m, 2 × 2-H and 2 × 3-H); ^{13}C NMR (100 MHz, $CDCl_3$) δ 14.2 (12-CH_3), 22.8 (CH_2), 29.3 (CH_2), 29.4 (CH_2), 29.5 (CH_2), 31.5 (6-CH_2), 31.9 (CH_2),

35.7 (5-CH_2), 125.7 (2-CH), 128.6 (1-C), 129.6 (3-CH), 134.6 (CS), 142.7 (4-C); ν_{max} (neat)/cm^{-1} 2953, 2915, 2847, 2189, 2140, 1501, 925, 835; HRMS (EI) calc. for $[C_{15}H_{21}NS]^+$ 247.1395, found 247.1401.

1.3 General Procedure I for synthesis of decalin transporters 7a-f

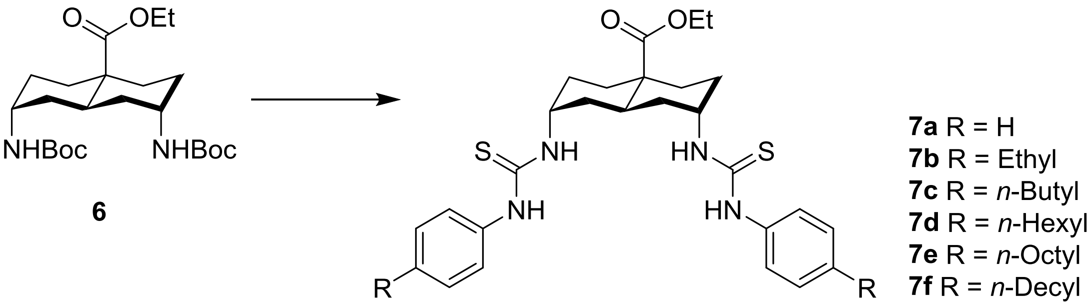

To a solution of Boc-protected diamine **6** (50 mg, 0.11 mmol) in anhydrous DCM (4 mL) was added TFA (1 mL) dropwise over 5 min and the solution was stirred at room temperature, under $N_{2,}$ for 16 h. The solvent was removed *in vacuo* and excess TFA was removed by co-evaporation with toluene and MeOH. The resulting white solid was dried under high vacuum for 1 h. To this solid was added DMAP (36 mg, 0.30 mmol) and anhydrous THF (10 mL). DIPEA (1 mL, 6 mmol) was then added, followed by the appropriate isothiocyanate (2.2 eq, 0.25 mmol) and the reaction mixture left to stir at r.t. for 16 h. The solvent was removed *in vacuo* and the crude material was redissolved in EtOAc (100 mL), washed with aqueous H_2SO_4 (25 mL, 0.25 M), saturated aqueous $NaHCO_3$ (25 mL), dried ($MgSO_4$), filtered and concentrated *in vacuo*. The crude mixture was purified by flash column chromatography.

Transporter 7a (R=H)

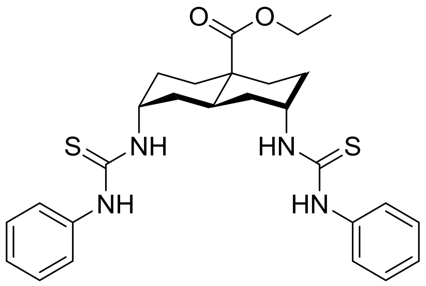

Prepared according to General Procedure I using phenyl isothiocyanate (30 µL, 0.25 mmol) and purified by flash column chromatography (1% MeOH in DCM) to give decalin bis-thiourea **7a** as an off-white solid (52 mg, 90%). R_f 0.1 (1% MeOH in DCM); ^{1}H NMR (500 MHz, acetone-d_6) δ 1.26 (3H, t, *J* 7.1, 11-H_3), 1.35-1.47 (2H, m, 4-H_{ax} and 5-H_{ax}), 1.52 (2H, tt, *J* 14.5 and 3.6, 3-H_{ax} and 6-H_{ax}), 1.67 (2H, ddd, *J* 14.2, 3.8 and 2.1, 1-H_{eq} and 8-H_{eq}), 1.72-1.82 (1H, m, 8a-H), 1.93-2.02 (4H, m, 4-H_{eq} and 5-H_{eq}, 3-H_{eq} and 6-H_{eq},), 2.20 (2H, td, *J* 13.6 and 4.3, 1-H_{ax} and 8-H_{ax}), 4.19 (2H, q, *J* 7.1, 10-H_2), 4.58 (2H,

br s, 2-H and 7-H), 7.09 (2H, tt, *J* 7.4 and 1.2, 2 × 16-H), 7.21 (2H, d, *J* 7.2, 2α-NH and 7α-NH), 7.32 (4H, dd, *J* 8.5 and 7.4, 4 × 15-H), 7.43-7.61 (4H, m, 4 × 14-H), 8.78 (2H, br s, 2 × Ph-NH); ^{13}C NMR (126 MHz, acetone-d$_6$) δ 14.6 (11-CH_3), 27.7 (3-CH_2 and 6-CH_2), 33.5 (1-CH_2 and 8-CH_2), 33.5 (4-CH_2 and 5-CH_2), 35.6 (8a-CH), 48.3 (4a-C), 50.2 (2-CH and 7-CH), 60.6 (10-CH_2), 124.1 (14-CH), 125.40 (16-CH), 129.6 (15-CH), 140.2 (13-C), 174.7 (9-CO), 181.3 (12-CS); ν_{max} (neat)/cm^{-1} 3270 (N–H), 2926 (C–H), 2857 (C–H), 1719 (C=O), 1598, 1526, 1495, 1192, 694; HRMS (ESI) calc. for $[C_{27}H_{34}N_4O_2S_2Na]^+$ 533.2015, found 533.2010.

Transporter 7b (R= Ethyl)

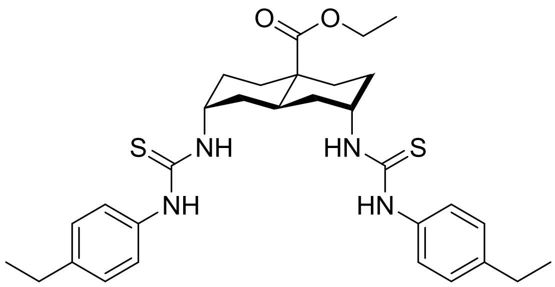

Prepared according to General Procedure I using 4-ethylphenyl isothiocyanate (36 μL, 0.24 mmol) and purified by flash column chromatography (1% MeOH in DCM) to give decalin bis-thiourea **7b** as a light brown solid (60 mg, 94%). R_f 0.2 (1% MeOH in DCM); ^{1}H NMR (400 MHz, acetone-d$_6$) δ 1.16 (6H, t, *J* 7.5, 2 × 18-H_3), 1.26 (3H, t, *J* 7.1, 11-H_3), 1.34-1.46 (2H, m, 4-H_{ax} and 5-H_{ax}), 1.53 (2H, tt, *J* 14.6 and 3.6, 3-H_{ax} and 6-H_{ax}), 1.67-1.75 (2H, m, 1-H_{eq} and 8-H_{eq}), 1.75-1.82 (1H, m, 8a-H), 1.92-2.03 (4H, m, 4-H_{eq} and 5-H_{eq}, 3-H_{eq} and 6-H_{eq}), 2.20 (2H, td, *J* 13.6 and 4.3, 1-H_{ax} and 8-H_{ax}), 2.54 (4H, q, *J* 7.5, 2 × 17-H_2), 4.19 (2H, q, *J* 7.1, 10-H_2), 4.56 (2H, br s, 2-H and 7-H), 7.14 (2H, br s, 2α-NH and 7α-NH), 7.16 (4H, d, *J* 8.0, 4 × 15-H), 7.38 (4H, d, *J* 8.0, 4 × 14-H), 8.71 (2H, br s, 2 × Ph-NH); ^{13}C NMR (100 MHz, acetone-d$_6$) δ 14.6 (11-CH_3), 16.0 (18-CH_3), 27.8 (3-CH_2 and 6-CH_2), 28.8 (17-CH_2), 33.5 (1-CH_2 and 8-CH_2), 33.5 (4-CH_2 and 5-CH_2), 35.6 (8a-CH), 48.3 (4a-C), 50.2 (2-CH and 7-CH), 60.6 (10-CH_2), 124.5 (14-CH), 129.0 (15-CH), 137.8 (13-C), 141.6 (16-C), 174.8 (9-CO), 181.3 (12-CS); ν_{max} (neat)/cm^{-1} 3369 (N-H), 2932 (C-H), 2842 (C-H), 1659 (C=O), 1602, 1544, 1319, 1229, 1160, 1108, 1065, 1014, 837; HRMS (ESI) calc. for $[C_{31}H_{42}N_4O_2S_2Na]^+$ 589.2641, found 589.2618.

Transporter 7c (R= Butyl)

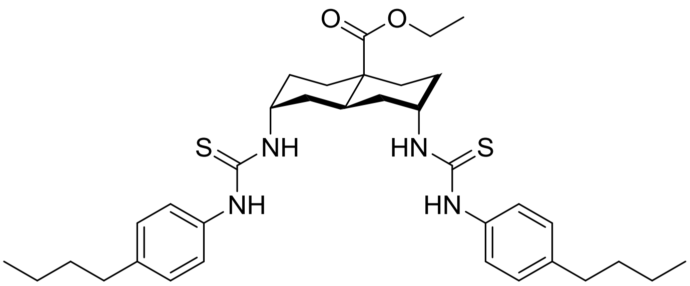

Prepared according to General Procedure I using 4-butylphenyl isothiocyanate (48 μL, 0.26 mmol) and purified by flash column chromatography (1% MeOH in DCM) to give decalin bis-thiourea **7c** as a white solid (59 mg, 80%). R_f 0.3 (1% MeOH in DCM); ^{1}H NMR (500 MHz, acetone-d_6) δ 0.90 (6H, t, *J* 7.3, 2 × 20-H_3), 1.26 (3H, t, *J* 7.1, 11-H_3), 1.28-1.46 (6H, m, 4-H_{ax} and 5-H_{ax}, 2 × 19-H_2), 1.46-1.61 (6H, m, 3-H_{ax} and 6-H_{ax}, 2 × 18-H_2), 1.63-1.73 (2H, m, 1-H_{eq} and 8-H_{eq}), 1.73-1.85 (1H, m, 8a-H), 1.87-2.02 (4H, m, 4-H_{eq} and 5-H_{eq}, 3-H_{eq} and 6-H_{eq}), 2.20 (2H, td, *J* 13.5 and 4.3, 1-H_{ax} and 8-H_{ax}), 2.54 (4H, t, *J* 7.7, 2 × 17-H_2), 4.18 (2H, q, *J* 7.1, 10-H_2), 4.57 (2H, br s, 2-H and 7-H), 7.15 (6H, app. d, *J* 8.3, 4 × 15-H, 2α-NH and 7α-NH), 7.38 (4H, d, *J* 8.3, 4 × 14-H), 8.70 (2H, br s, 2 × Ph-NH); ^{13}C NMR (126 MHz, acetone-d_6) δ 14.2 (20-CH_3), 14.6 (11-CH_3), 23.0 (19-CH_2), 27.8 (3-CH_2 and 6-CH_2), 33.5 (1-CH_2 and 8-CH_2), 33.6 (4-CH_2 and 5-CH_2), 34.4 (18-CH_2), 35.6 (8a-CH), 35.6 (17-CH_2), 48.4 (4a-C), 50.3 (2-CH and 7-CH), 60.7 (10-CH_2), 124.4 (14-CH), 129.5 (15-CH), 137.8 (13-C), 140.2 (16-C), 174.8 (9-CO), 181.3 (12-CS); ν_{max} (neat)/cm^{-1} 3269 (N–H), 2955 (C–H), 2925 (C–H), 2856 (C–H), 1721 (C=O), 1527, 1511, 1192; HRMS (ESI) calc. for $[C_{35}H_{50}N_4O_2S_2Na]^+$ 645.3267, found 645.3271.

Transporter 7d (R= Hexyl)

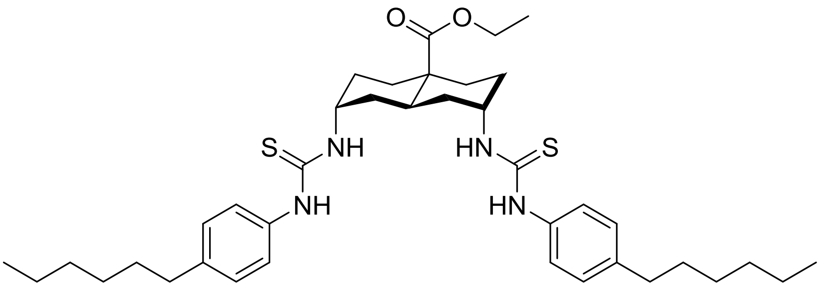

Prepared according to General Procedure I using 4-hexylphenyl isothiocyanate **8d** (53 mg, 0.24 mmol) and purified by flash column chromatography (1% MeOH in DCM) to give decalin bis-thiourea **7d** as an off-white solid (51 mg, 68%). R_f 0.3 (1% MeOH in DCM); ^{1}H NMR (500 MHz, acetone-d_6) δ 0.88 (6H, t, *J* 6.7, 2 × 22-H_3), 1.26 (3H, t, *J* 7.1, 11-H_3), 1.28-1.43 (14H, m, 4-H_{ax} and 5-H_{ax}, 2 × 19-H_2, 2 × 20-H_2 and 2 × 21-H_2), 1.47-1.62 (6H, m, 3-H_{ax} and 6-H_{ax} and 2 × 18-H_2), 1.69-1.80 (3H, m, 8a-H, 1-H_{eq} and 8-H_{eq}), 1.92-2.02 (4H, m, 4-H_{eq} and 5-H_{eq}, 3-H_{eq} and 6-H_{eq}), 2.20 (2H, td, *J* 13.3 and 4.6, 1-H_{ax} and 8-H_{ax}), 2.54 (4H, t, *J* 7.7, 2 × 17-H_2), 4.19 (2H, q, *J* 7.1, 10-H_2), 4.56 (2H, br s, 2-H and 7-H), 7.08

(2H, br s, 2α-NH and 7α-NH), 7.17 (4H, d, *J* 8.3, 4 × 15-H), 7.37 (4H, d, *J* 8.3, 4 × 14-H), 8.66 (2H, br s, 2 × Ph-NH); ^{13}C NMR (126 MHz, acetone-d$_6$) δ 14.4 (22-CH$_3$), 14.6 (11-CH$_3$), 23.2 (21-CH$_2$), 27.8 (3-CH$_2$ and 6-CH$_2$), 29.7 (19-CH$_2$), 32.3 (18-CH$_2$), 32.5 (20-CH$_2$), 33.5 (1-CH$_2$ and 8-CH$_2$), 33.6 (4-CH$_2$ and 5-CH$_2$), 35.8 (8a-CH), 35.9 (17-CH$_2$), 48.4 (4a-C), 50.3 (2-CH and 7-CH), 60.7 (10-CH$_2$), 124.3 (14-CH), 129.6 (15-CH), 137.7 (13-C), 140.3 (16-C), 174.8 (9-CO), 181.5 (12-CS); ν_{max} (neat)/cm^{-1} 3251 (N–H), 2924 (C–H), 2855 (C–H), 1721 (C=O), 1509, 1192, 667; HRMS (ESI) calc. for $[C_{39}H_{58}N_4O_2S_2Na]^+$ 701.3893, found 701.3885.

Transporter 7e (R= Octyl)

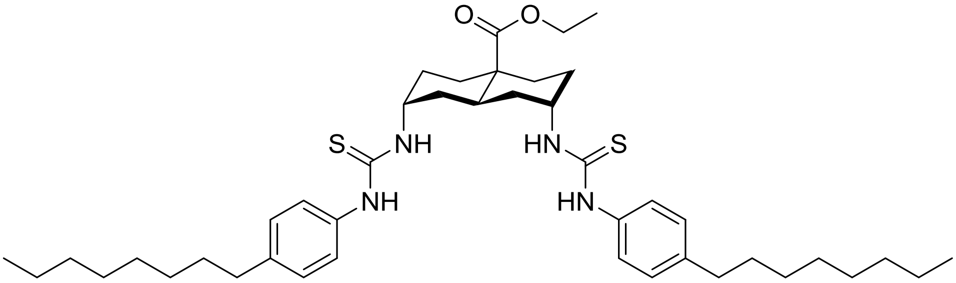

Prepared according to General Procedure I using 4-octylphenyl isothiocyanate **8e** (63 mg, 0.26 mmol) and purified by flash column chromatography (1% MeOH in DCM) to give decalin bis-thiourea **7e** as an off-white solid (40 mg, 48%). R$_f$ 0.4 (1% MeOH in DCM); ^{1}H NMR (500 MHz, acetone-d$_6$) δ 0.88 (6H, t, *J* 6.9, 2 × 24-H$_3$), 1.26 (3H, t, *J* 7.1, 11-H$_3$), 1.27-1.35 (22H, m, 4-H$_{ax}$ and 5-H$_{ax}$, 2 × 19-H$_2$, 2 × 20-H$_2$ and 2 × 21-H$_2$, 2 × 22-H$_2$, 2 × 23-H$_2$), 1.48-1.62 (6H, m, 3-H$_{ax}$ and 6-H$_{ax}$ and 2 × 18-H$_2$), 1.66-1.79 (3H, m, 8a-H, 1-H$_{eq}$ and 8-H$_{eq}$), 1.93-2.02 (4H, m, 4-H$_{eq}$ and 5-H$_{eq}$, 3-H$_{eq}$ and 6-H$_{eq}$), 2.20 (2H, td, *J* 13.5 and 4.3, 1-H$_{ax}$ and 8-H$_{ax}$), 2.54 (4H, t, *J* 7.8, 2 × 17-H$_2$), 4.18 (2H, q, *J* 7.1, 10-H$_2$), 4.55 (2H, br s, 2-H and 7-H), 7.07 (2H, br s, 2α-NH and 7α-NH), 7.18 (4H, d, *J* 8.3, 4 × 15-H), 7.37 (4H, d, *J* 8.3, 4 × 14-H), 8.65 (2H, br s, 2 × Ph-NH); ^{13}C NMR (126 MHz, acetone-d$_6$) δ 14.4 (24-CH$_3$), 14.6 (11-CH$_3$), 23.3 (23-CH$_2$), 27.8 (3-CH$_2$ and 6-CH$_2$), 29.8 (CH$_2$), 30.1 (CH$_2$), 30.2 (CH$_2$), 32.3 (CH$_2$), 32.6 (CH$_2$), 33.5 (1-CH$_2$ and 8-CH$_2$), 33.6 (4-CH$_2$ and 5-CH$_2$), 35.7 (8a-CH), 36.0 (17-CH$_2$), 48.4 (4a-C), 50.3 (2-CH and 7-CH), 60.7 (10-CH$_2$), 124.3 (14-CH), 129.6 (15-CH), 137.7 (13-C), 140.3 (16-C), 174.8 (9-CO), 181.5 (12-CS) ν_{max} (neat)/cm^{-1} 3271 (N–H), 2923 (C–H), 2853 (C–H), 1722 (C=O), 1526, 1509, 1192, 663; MS (ESI) calc. for $[C_{39}H_{58}N_4O_2S_2Na]^+$ 757.5, found 757.6.

Transporter 7f (R= Decyl)

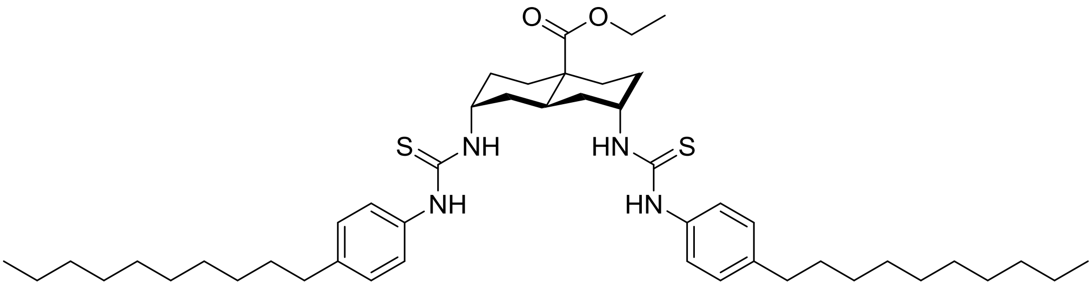

Prepared according to General Procedure I using 4-decylphenyl isothiocyanate (69 mg, 0.25 mmol) and purified by flash column chromatography (25% EtOAc in hexane) to give decalin bis-thiourea **7f** as a white solid (42 mg, 47%). R_f 0.5 (1% MeOH in DCM); ^{1}H NMR (500 MHz, acetone-d_6) δ 0.88 (6H, t, *J* 6.7, 2 × 26-H_3), 1.03-1.47 (33H, m, 11-H_3, 4-H_{ax} and 5-H_{ax}, 2 × 19-H_2, 2 × 20-H_2, 2 × 21-H_2, 2 × 22-H_2, 2 × 23-H_2, 2 × 24-H_2, 2 × 25-H_2), 1.47-1.62 (6H, m, 3-H_{ax} and 6-H_{ax} and 2 × 18-H_2), 1.63-1.71 (2H, m, 1-H_{eq} and 8-H_{eq}), 1.71-1.81 (1H, m, 4a-H), 1.88-2.02 (4H, m, 4-H_{eq} and 5-H_{eq}, 3-H_{eq} and 6-H_{eq}), 2.20 (2H, td, *J* 13.2 and 4.1, 1-H_{ax} and 8-H_{ax}), 2.53 (4H, t, *J* 7.7, 2 × 17-H_2), 4.18 (2H, q, *J* 7.2, 10-H_2), 4.57 (2H, br s, 2-H and 7-H), 7.12 (2H, br s, 2α-NH and 7α-NH), 7.16 (4H, d, *J* 8.7, 4 × 15-H), 7.38 (4H, d, *J* 8.7, 4 × 14-H), 8.70 (2H, br s, 2 × Ph-NH); ^{13}C NMR (126 MHz, acetone-d_6) δ 14.4 (26-CH_3), 14.6 (11-CH_3), 23.3 (25-CH_2), 27.8 (3-CH_2 and 6-CH_2), 30.0 (CH_2), 30.1 (CH_2), 30.3 (CH_2), 30.4 (CH_2), 30.4 (CH_2), 32.3 (CH_2), 32.7 (CH_2), 33.5 (1-CH_2 and 8-CH_2), 33.5 (4-CH_2 and 5-CH_2), 35.7 (8a-CH), 36.0 (17-CH_2), 48.4 (4a-C), 50.3 (2-CH and 7-CH), 60.7 (10-CH_2), 124.3 (14-CH), 129.6 (15-CH), 137.7 (13-C), 140.3 (16-C), 174.8 (9-CO), 181.4 (12-CS); ν_{max} (neat)/cm^{-1} 3286 (N–H), 2921 (C–H), 2852 (C–H), 1723 (C=O), 1530, 1512, 1192; HRMS (ESI) calc. for $[C_{47}H_{74}N_4O_2S_2Na]^+$ 813.5145, found 813.5141; Elemental analysis $[C_{47}H_{74}N_4O_2S_2]$ calculated C 71.34, H 9.43, N 7.08, found C 71.34, H 9.49, N 6.91.

1.4 NMR Spectra of 7a-f

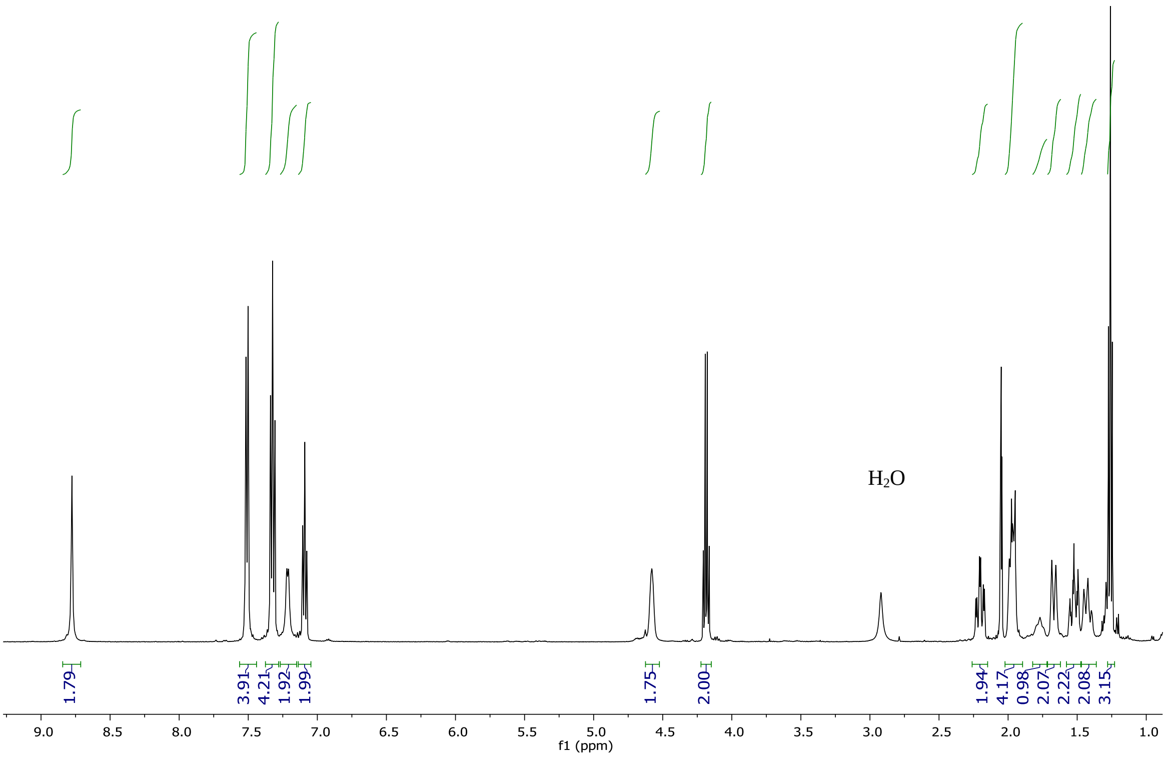

Figure S1. ^{1}H NMR spectrum of decalin bis-thiourea **7a** in acetone-d$_6$.

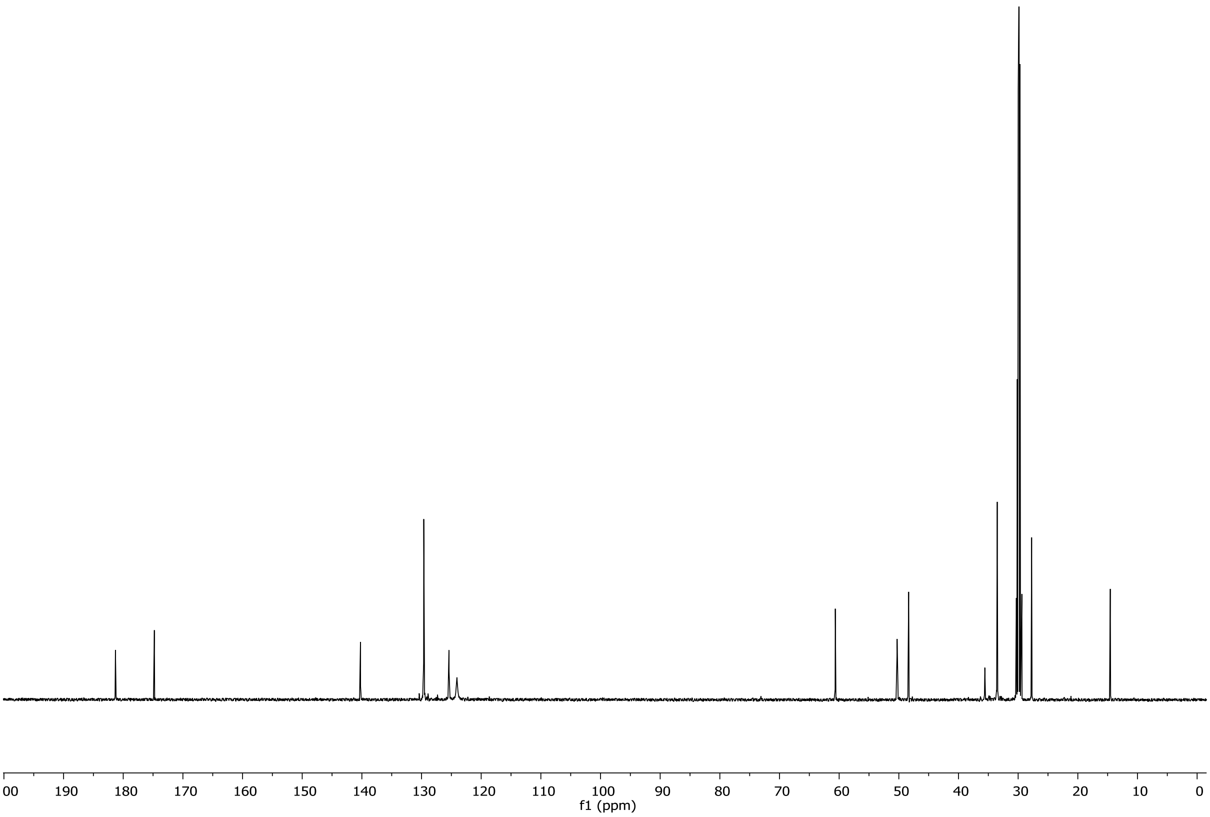

Figure S2. ^{13}C NMR spectrum of decalin bis-thiourea **7a** in acetone-d$_6$.

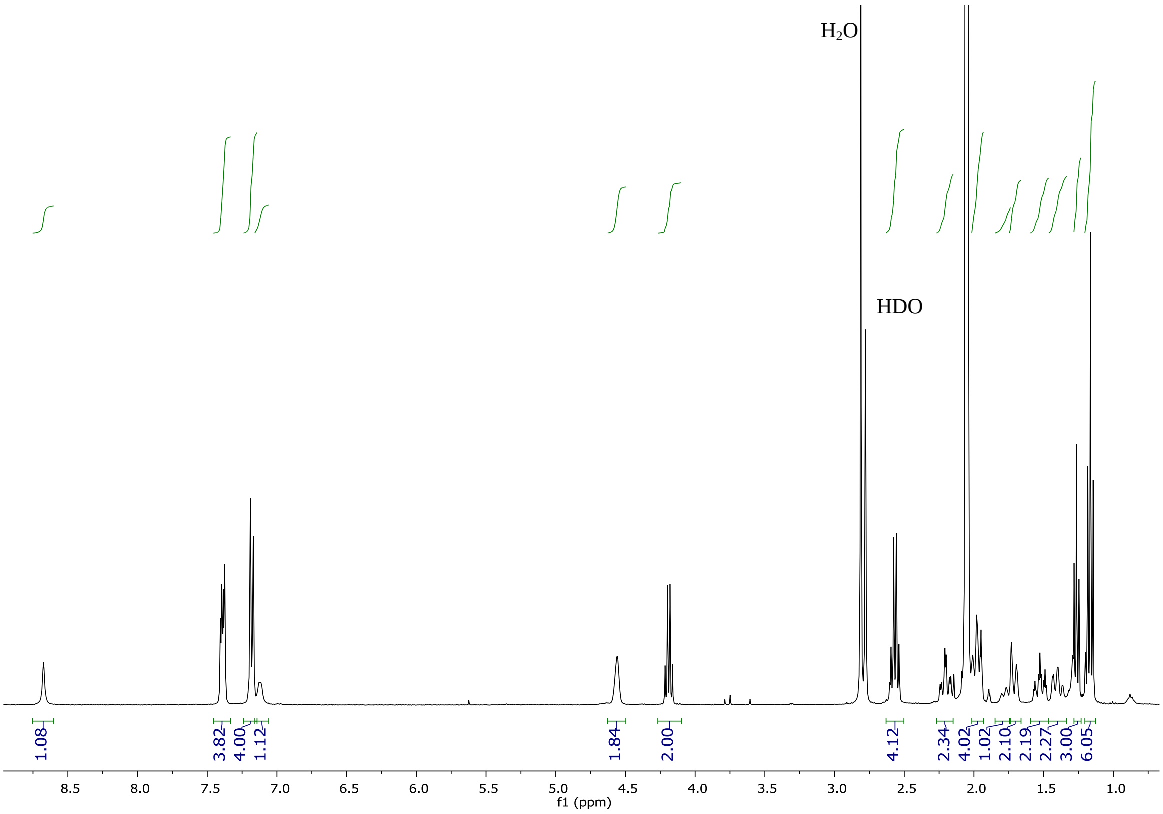

Figure S3. ^{1}H NMR spectrum of decalin bis-thiourea **7b** in acetone-d_6.

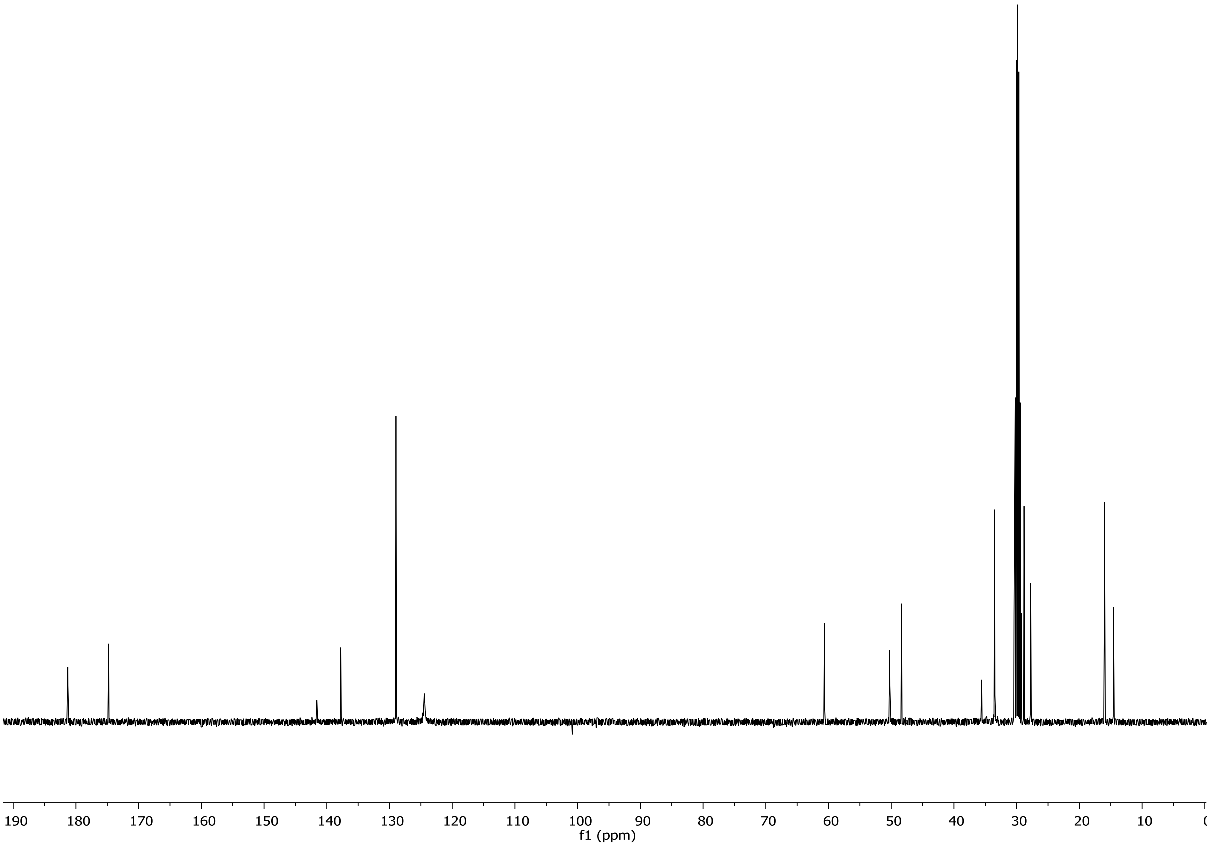

Figure S4. ^{13}C NMR spectrum of decalin bis-thiourea **7b** in acetone-d_6.

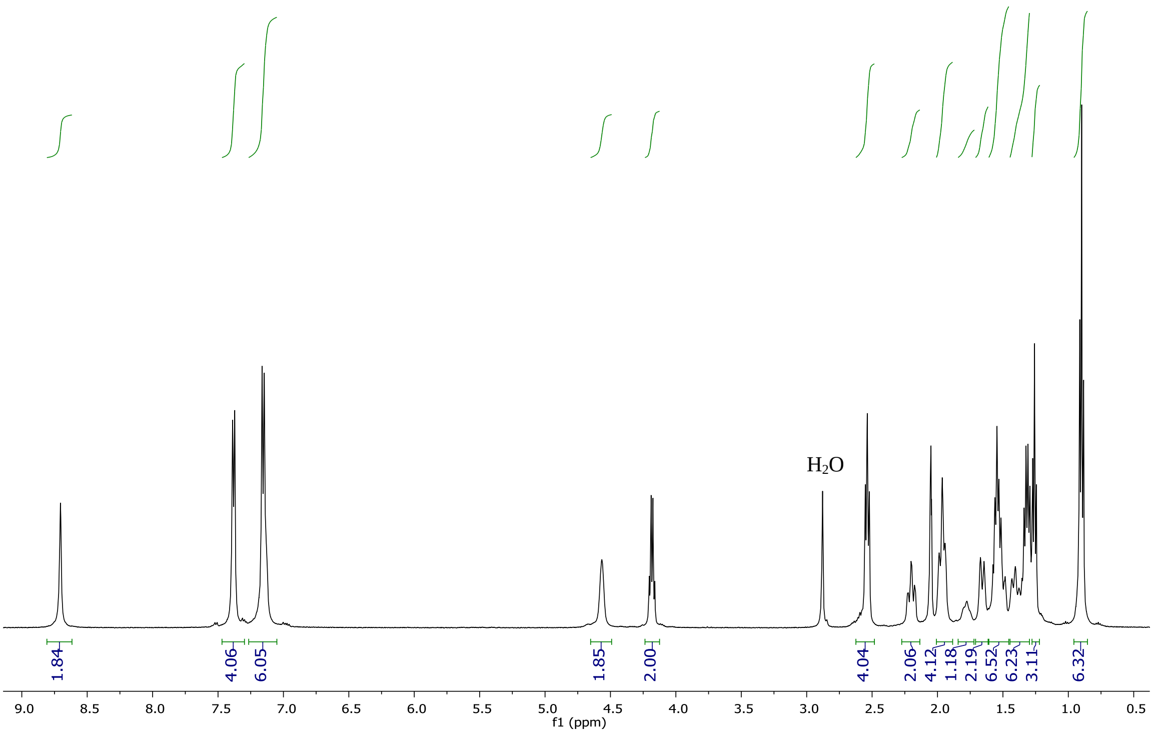

Figure S5. ^{1}H NMR spectrum of decalin bis-thiourea **7c** in acetone-d$_6$.

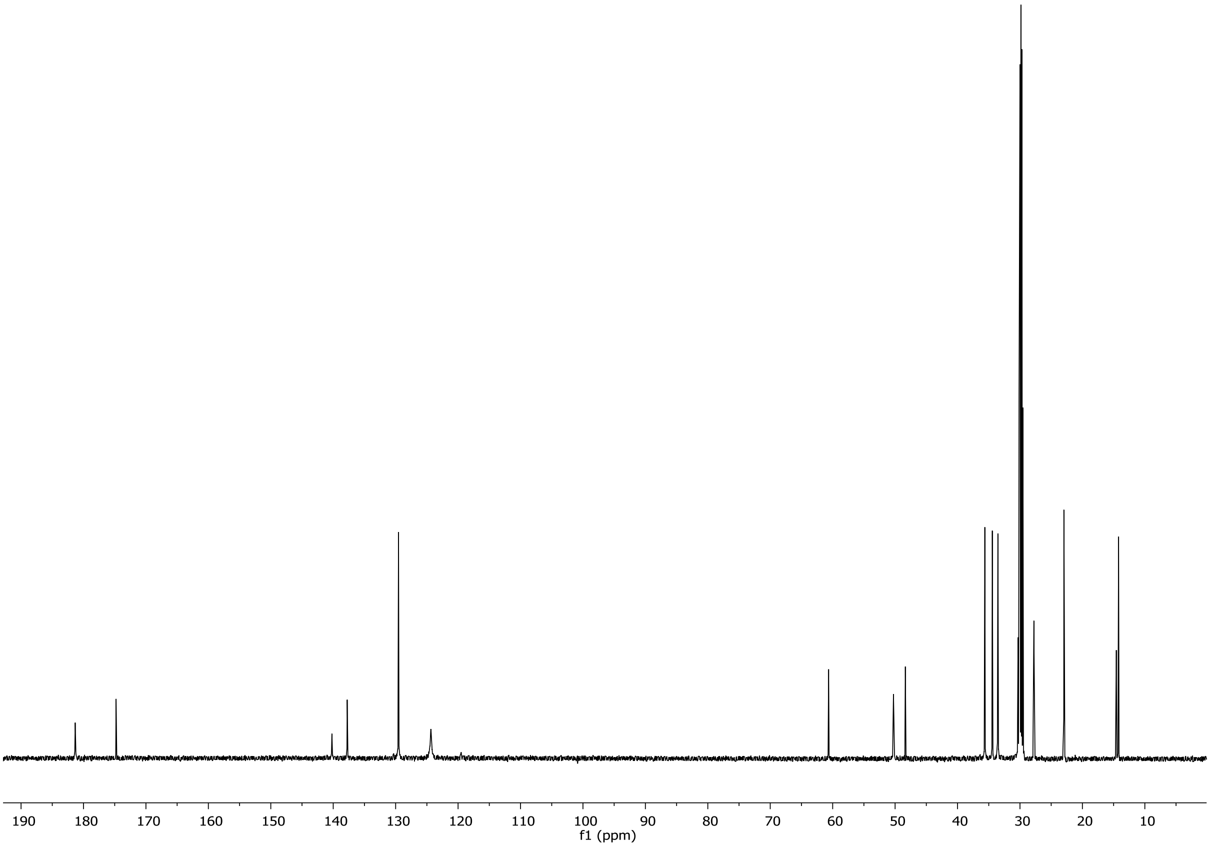

Figure S6. ^{13}C NMR spectrum of decalin bis-thiourea **7c** in acetone-d$_6$.

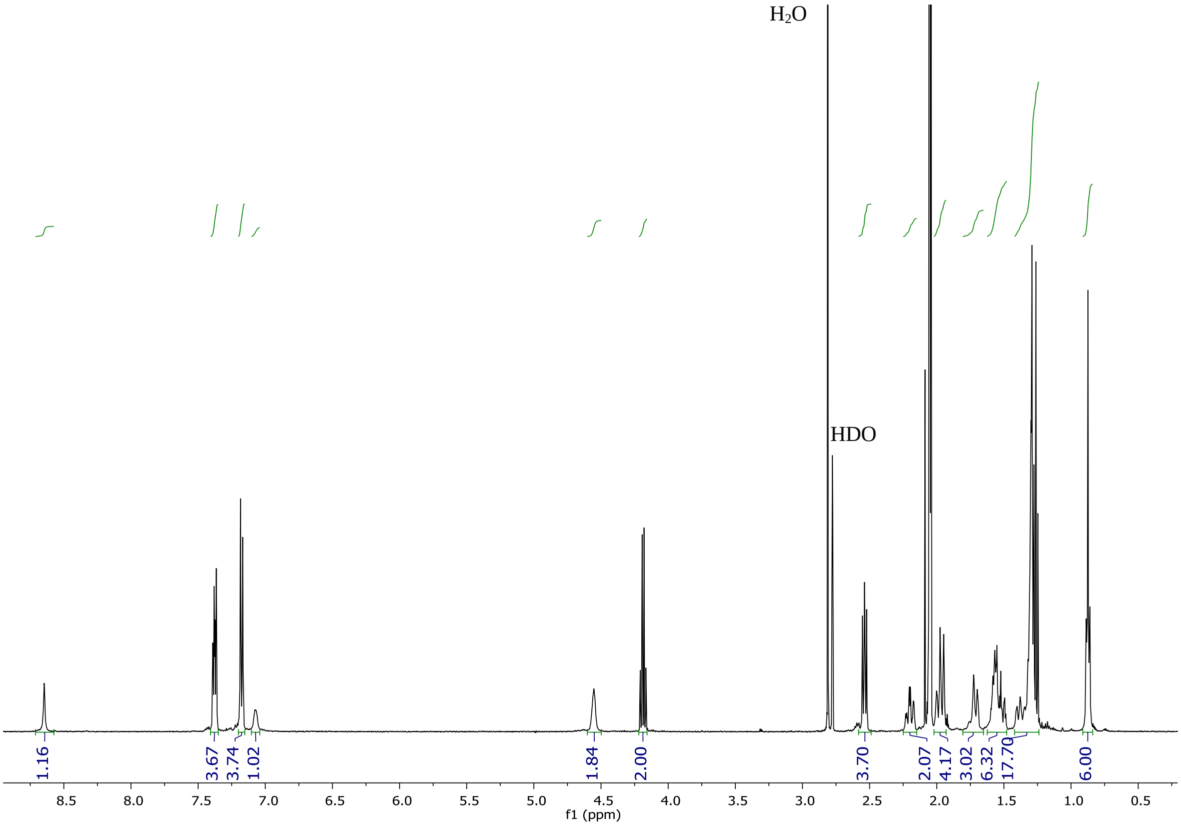

Figure S7. ^{1}H NMR spectrum of decalin bis-thiourea **7d** in acetone-d_6.

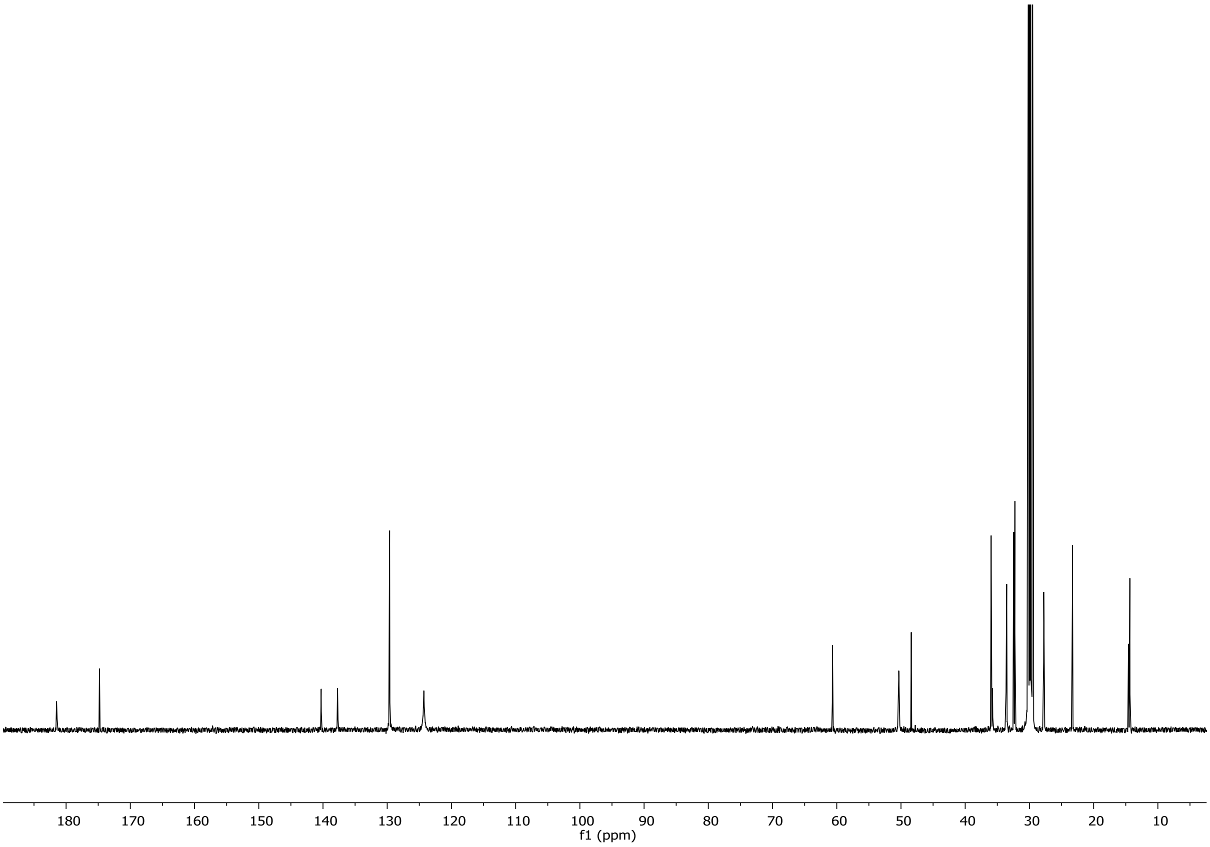

Figure S8. ^{13}C NMR spectrum of decalin bis-thiourea **7d** in acetone-d_6.

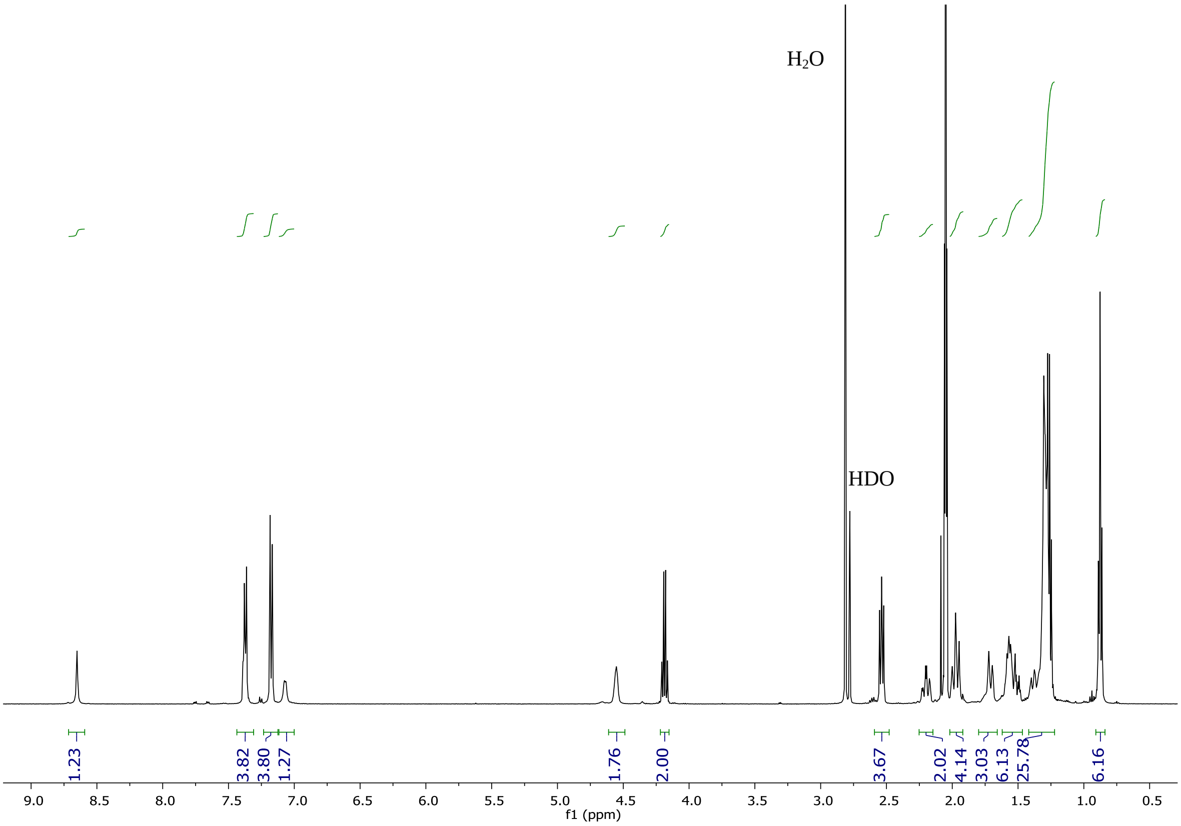

Figure S9. ^{1}H NMR spectrum of decalin bis-thiourea **7e** in acetone-d_6.

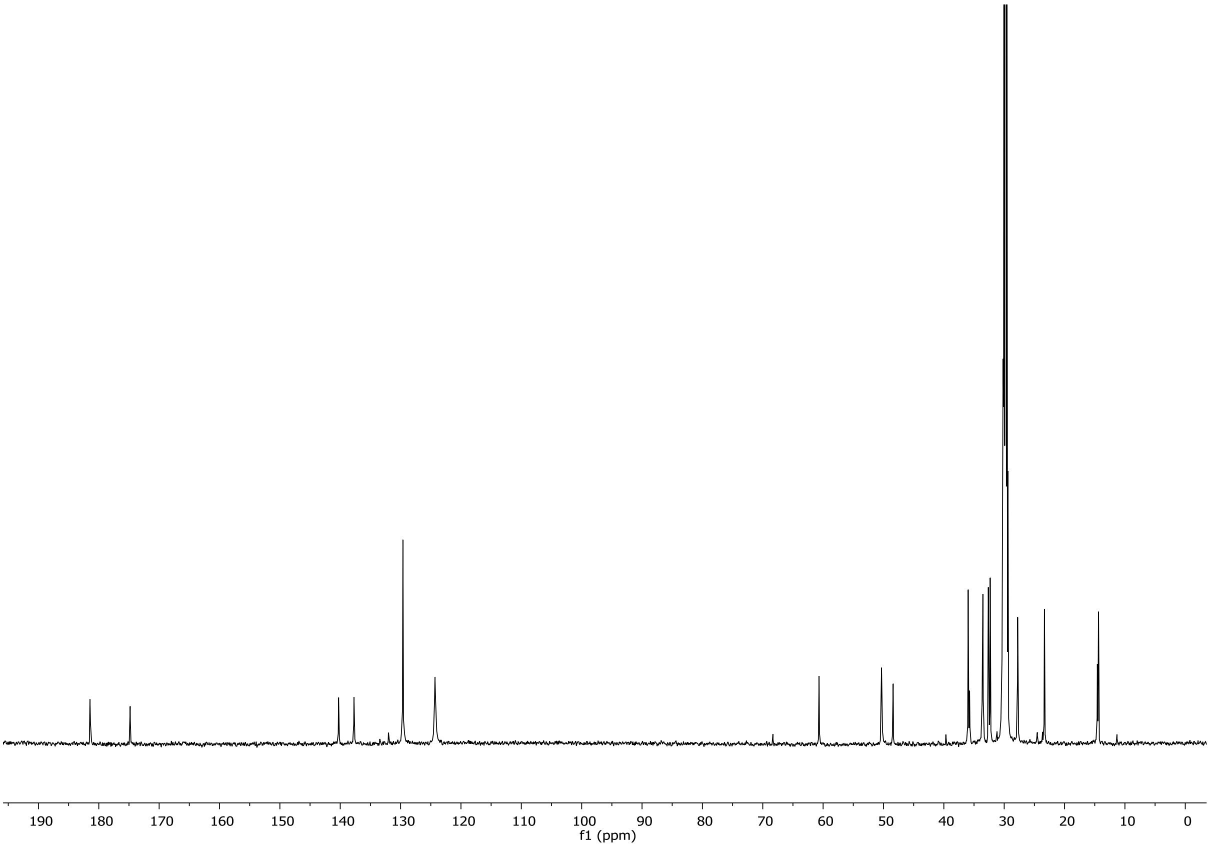

Figure S10. ^{13}C NMR spectrum of decalin bis-thiourea **7e** in acetone-d_6.

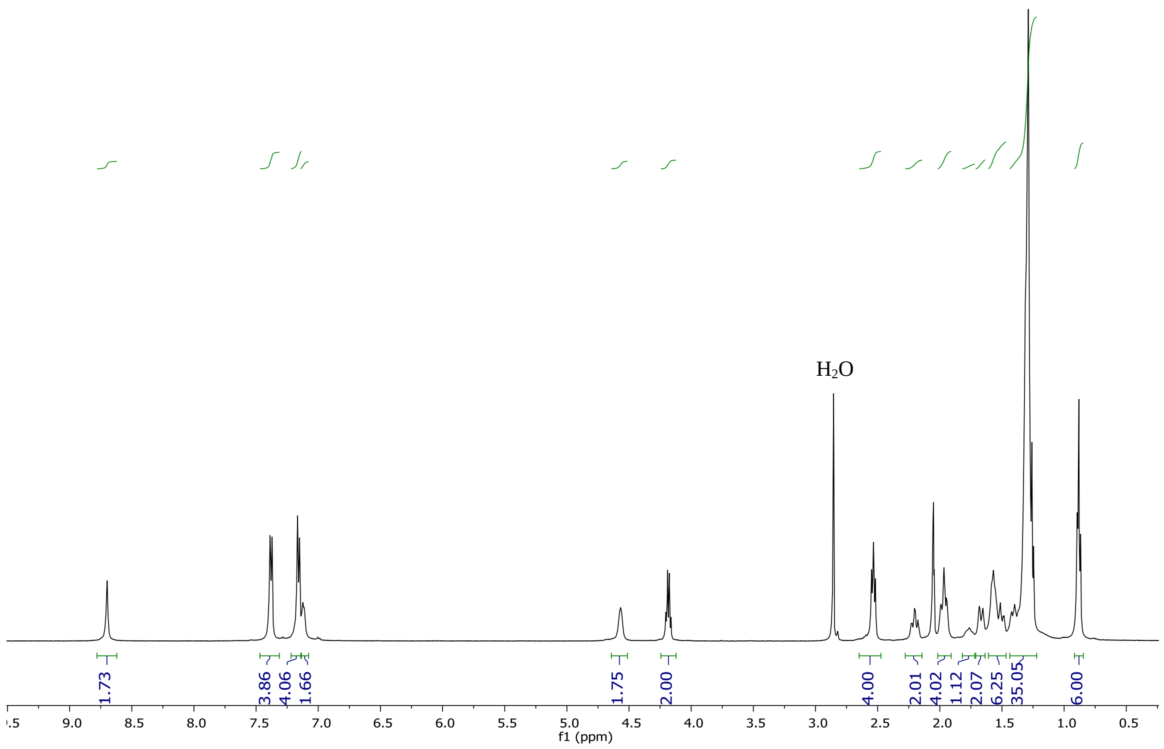

Figure S11. ^{1}H NMR spectrum of decalin bis-thiourea **7f** in acetone-d_6.

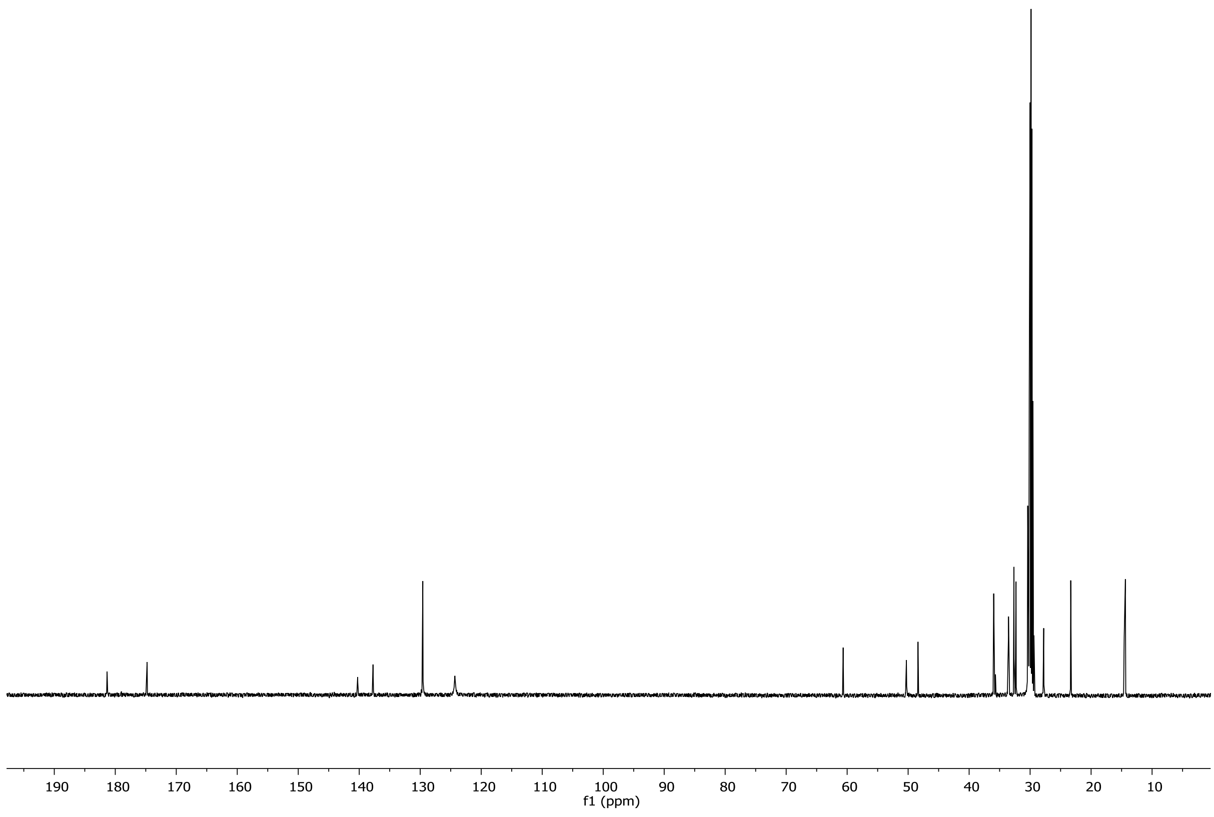

Figure S12. ^{13}C NMR spectrum of decalin bis-thiourea **7f** in acetone-d_6.

2. Anion Binding Affinities

2.1 Chloride binding affinities by ^{1}H NMR titration in DMSO-d_6

The binding affinities of receptors **7** for chloride were measured by ^{1}H NMR titrations against n-$Bu_4N^+Cl^-$ in DMSO-d_6 containing 0.5% H_2O. The hygroscopic guest n-$Bu_4N^+Cl^-$ and the host compounds were dried under high vacuum to remove residual solvents or water prior to solution preparation.

The concentration of the host was around 1 mM in all titrations; the concentration of guest and the volumes of the aliquots of guest solution added to the host solution were varied over the experiments. The guest solution contained the same concentrations of host as the starting host solution (*i.e.*, n-$Bu_4N^+Cl^-$ was dissolved in a solution of ~1 mM host in DMSO-d_6 with 0.5% H_2O), so that the concentration of host did not decrease over the course of the experiment.

All ^{1}H NMR titration binding studies were performed using a Varian 500 MHz NMR spectrometer (proton sensitive probe) at 298 K. Spectra were calibrated to the residual solvent peak in DMSO-d_6 (δ = 2.50 ppm). Binding constants were determined by fitting the shift of both NH signals to a 1:1 binding model using a least-square fitting procedure in a custom-made Excel spreadsheet.

^{1}H NMR titration of 7a with Bu_4NCl in DMSO-d_6/0.5% H_2O

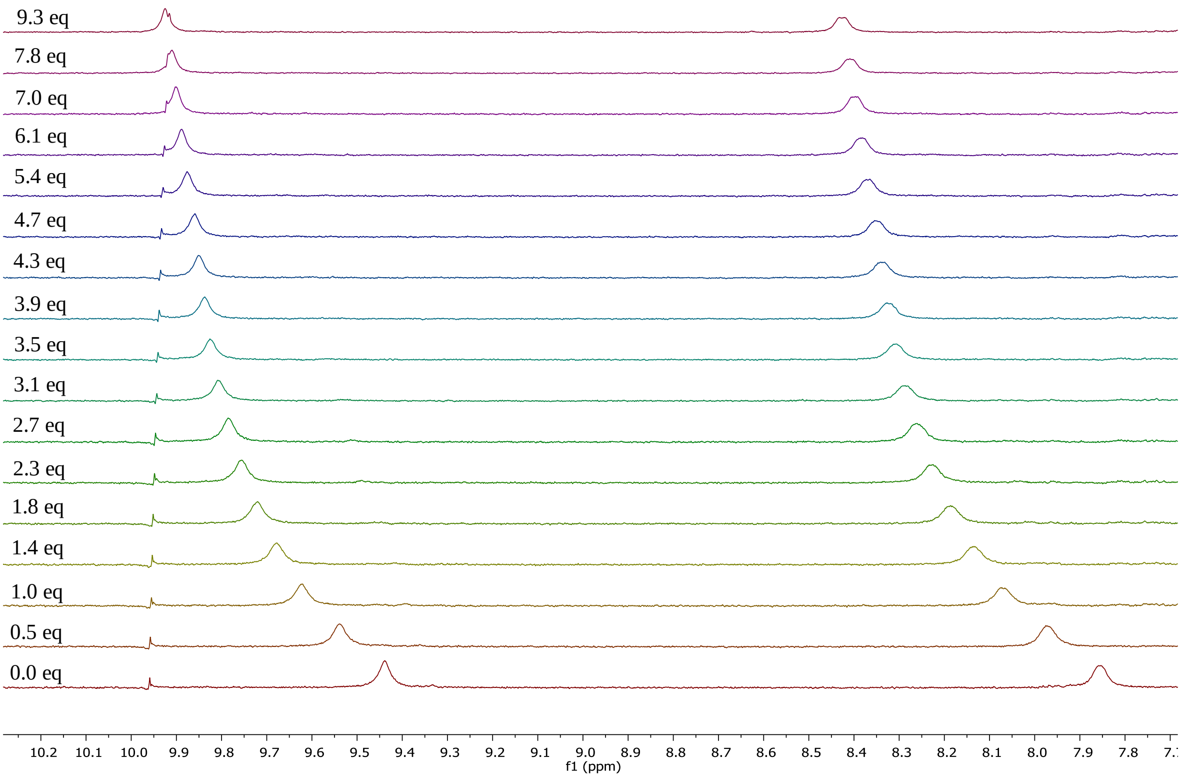

Figure S13. ^{1}H NMR spectra from the titration of $Bu_4N^+Cl^-$ into **7a** (1 mM) in DMSO-d_6/0.5% H_2O at 298 K. The amount of $Bu_4N^+Cl^-$ added is shown in equivalents relative to **7a**.

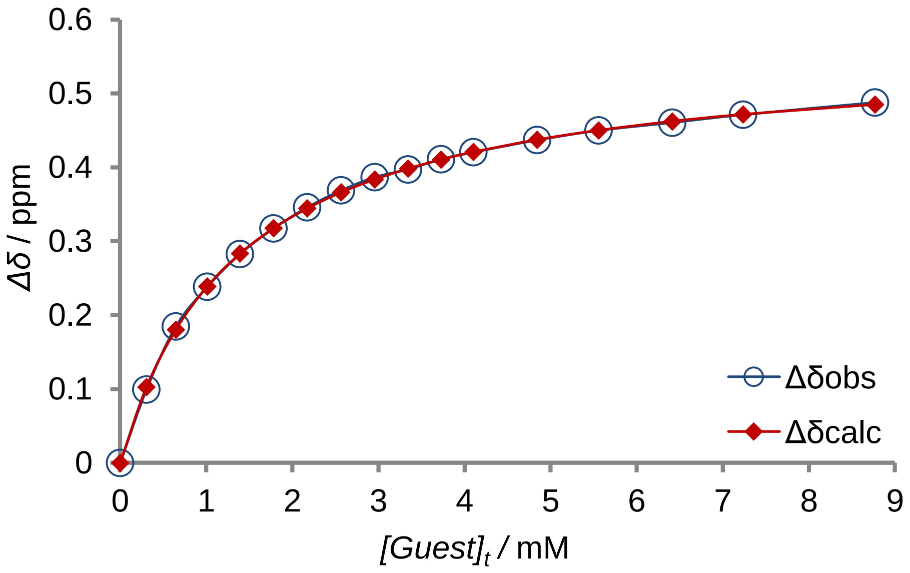

Figure S14. Graph showing the observed binding curve (red) and calculated fitting (blue) for **7a** when titrated against chloride at 298 K. The NH signal at δ = 9.43 ppm is plotted and both NH signals were used to find K_a = 734 ± 8 M^{-1}.

^{1}H NMR titration of 7b with Bu_4NCl in DMSO-d_6/0.5% H_2O

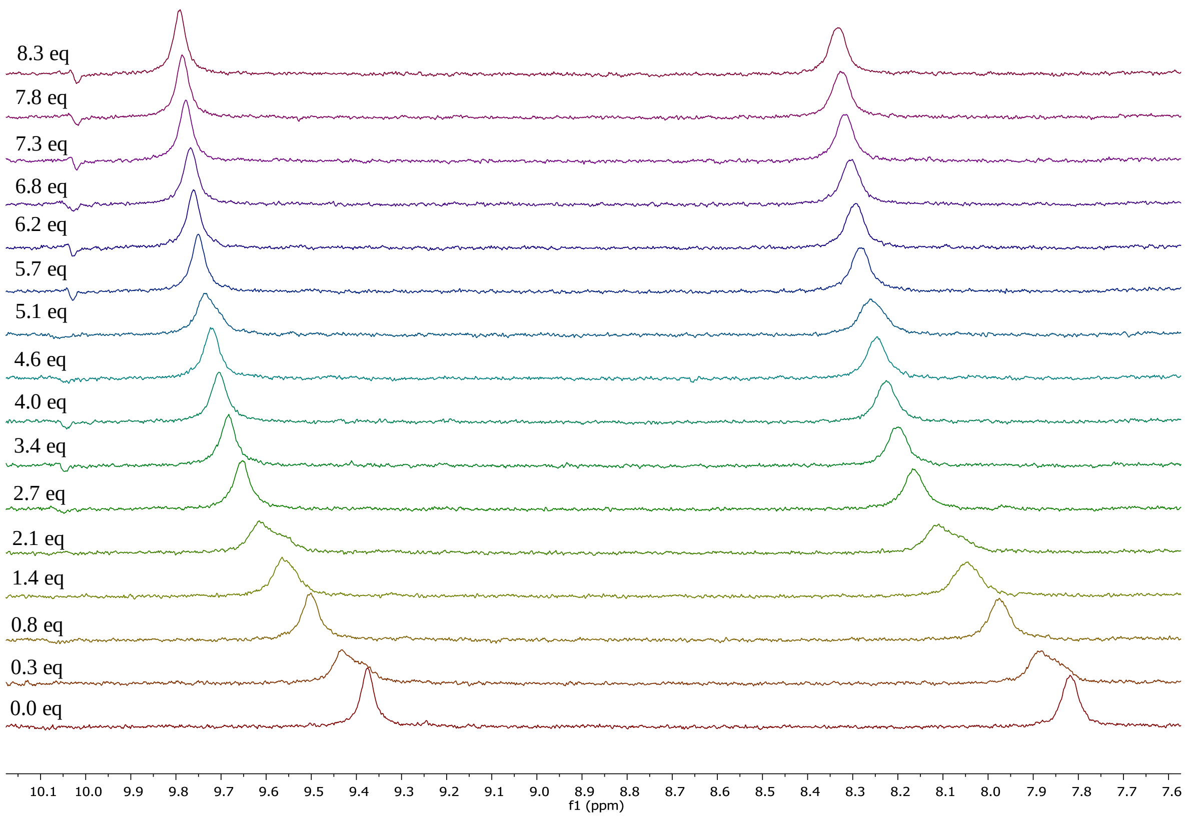

Figure S15. ^{1}H NMR spectra from the titration of $Bu_4N^+Cl^-$ into **7b** (1 mM) in DMSO-d_6/0.5% H_2O at 298 K. The amount of $Bu_4N^+Cl^-$ added is shown in equivalents relative to **7b**.

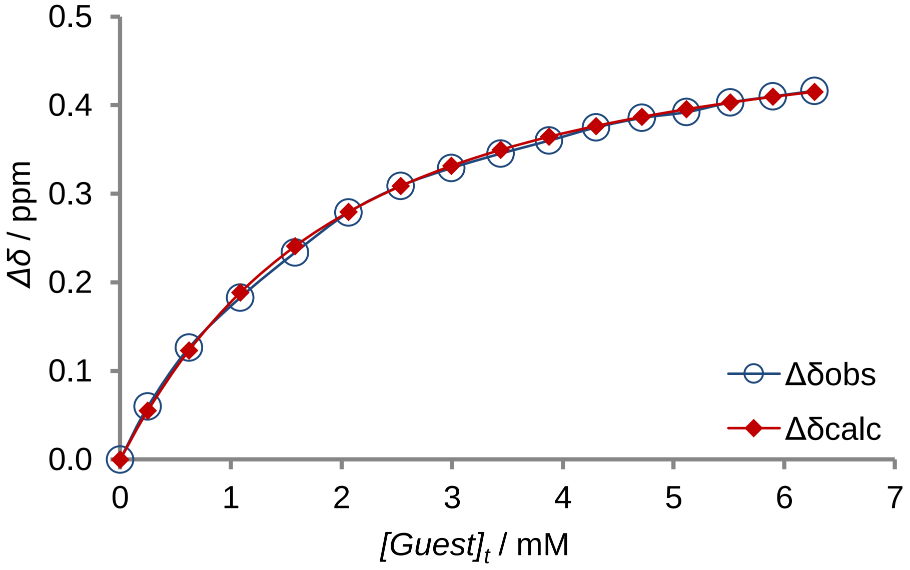

Figure S16. Graph showing the observed binding curve (red) and calculated fitting (blue) for **7b** when titrated against chloride at 298 K. The NH signal at δ = 9.37 ppm is plotted and both NH signals were used to find K_a = 703 ± 15 M^{-1}.

^{1}H NMR titration of 7c with Bu_4NCl in DMSO-d$_6$/0.5% H_2O

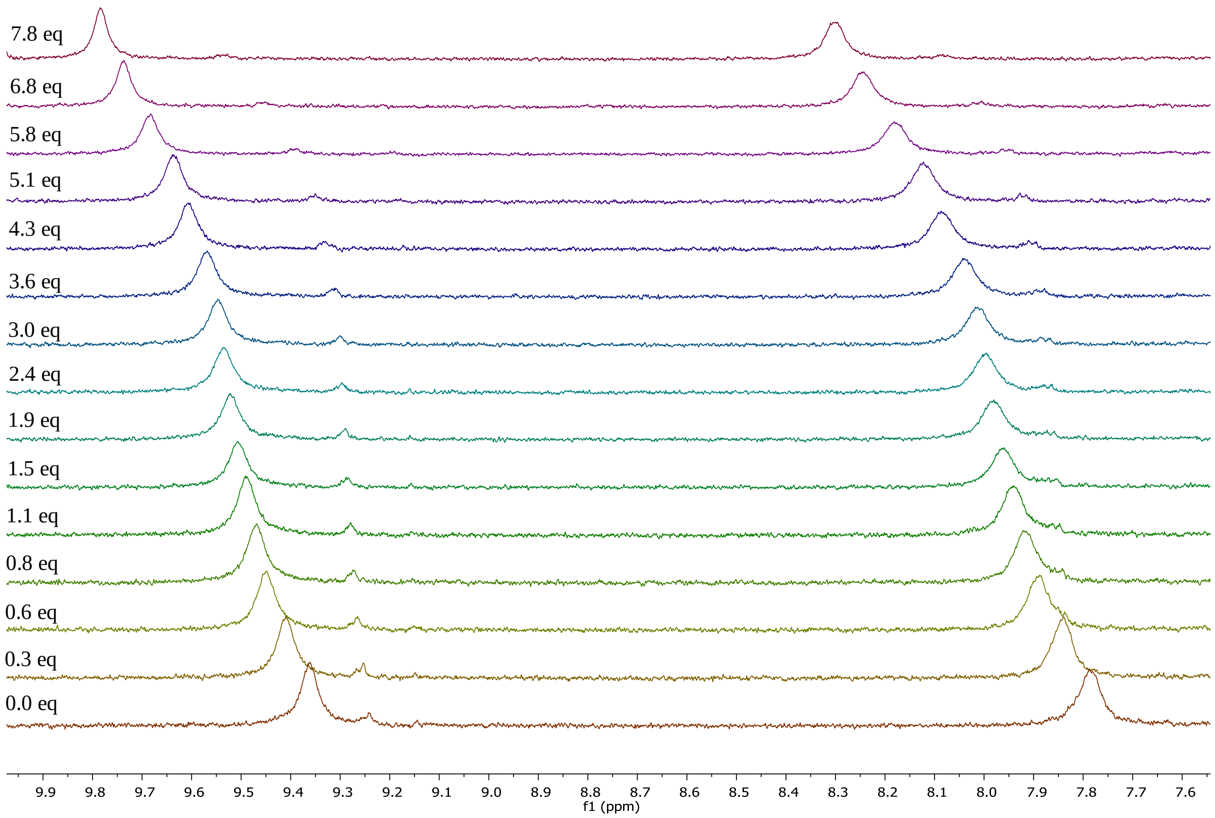

Figure S17. ^{1}H NMR spectra from the titration of $Bu_4N^+Cl^-$ into **7c** (1 mM) in DMSO-d$_6$/0.5% H_2O at 298 K. The amount of $Bu_4N^+Cl^-$ added is shown in equivalents relative to **7c**.

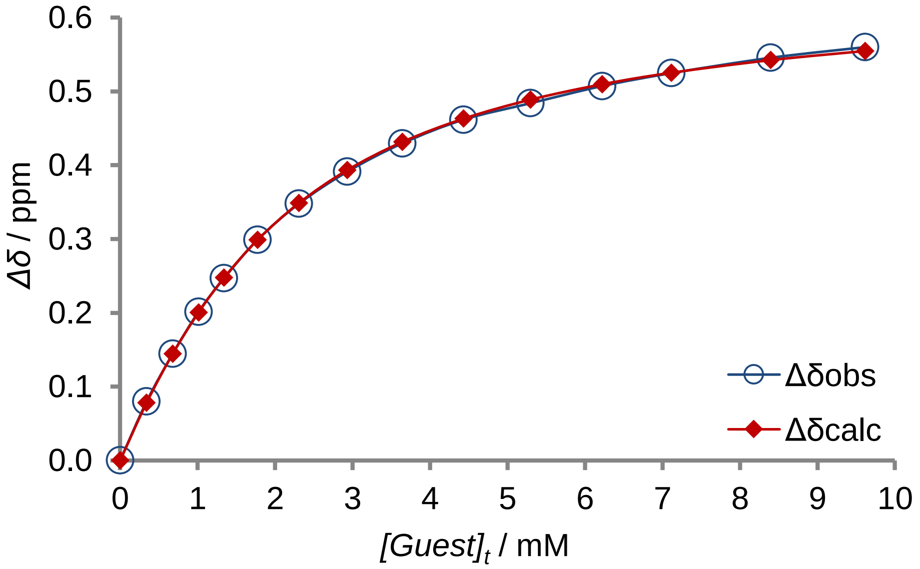

Figure S18. Graph showing the observed binding curve (red) and calculated fitting (blue) for **7c** when titrated against chloride at 298 K. The NH signal at δ = 7.85 ppm is plotted and both NH signals were used to find K_a = 715 ± 6 M^{-1}.

^{1}H NMR titration of 7d with Bu_4NCl in DMSO-d_6/0.5% H_2O

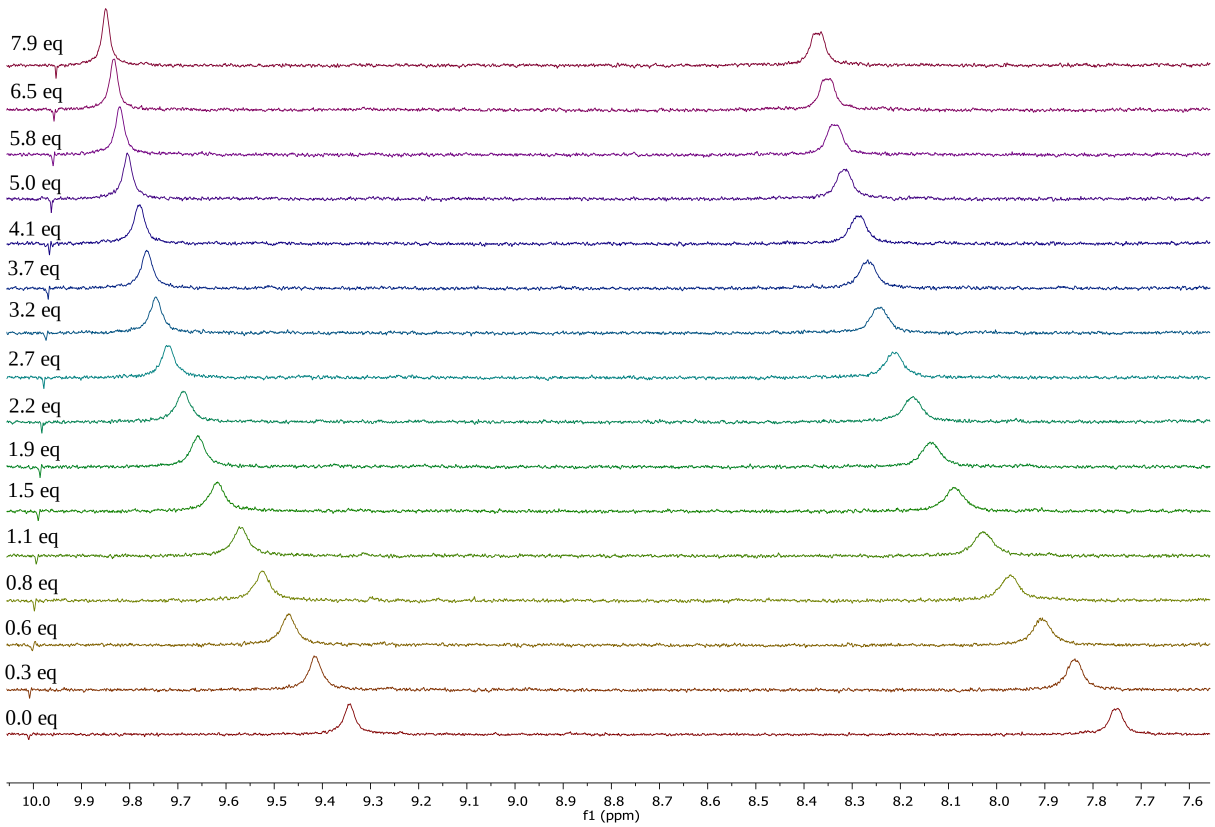

Figure S19. ^{1}H NMR spectra from the titration of $Bu_4N^+Cl^-$ into **7d** (1 mM) in DMSO-d_6/0.5% H_2O at 298 K. The amount of $Bu_4N^+Cl^-$ added is shown in equivalents relative to **7d**.

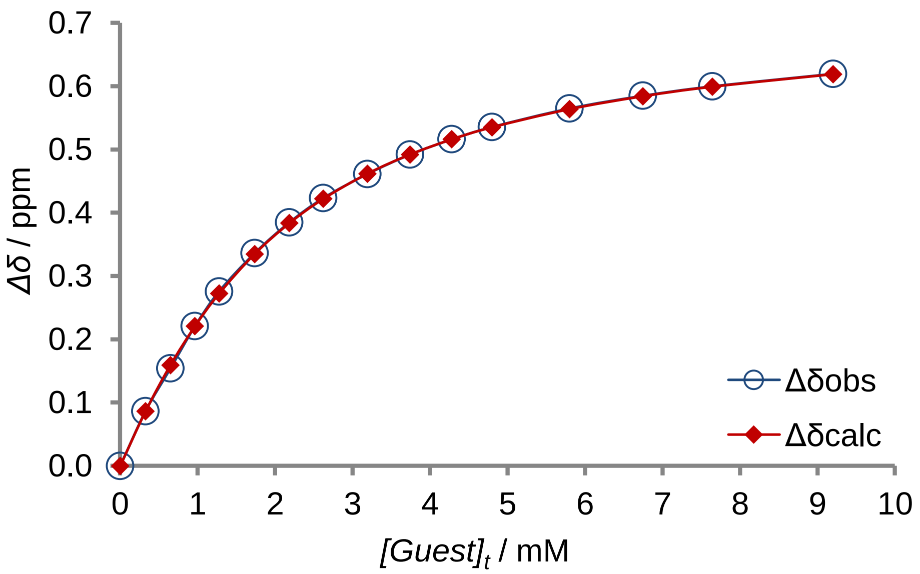

Figure S20. Graph showing the observed binding curve (red) and calculated fitting (blue) for **7d** when titrated against chloride at 298 K. The NH signal at δ = 7.75 ppm is plotted and both NH signals were used to find K_a = 722 ± 7 M^{-1}.

^1H NMR titration of 7e with Bu_4NCl in DMSO-d_6/0.5% H_2O

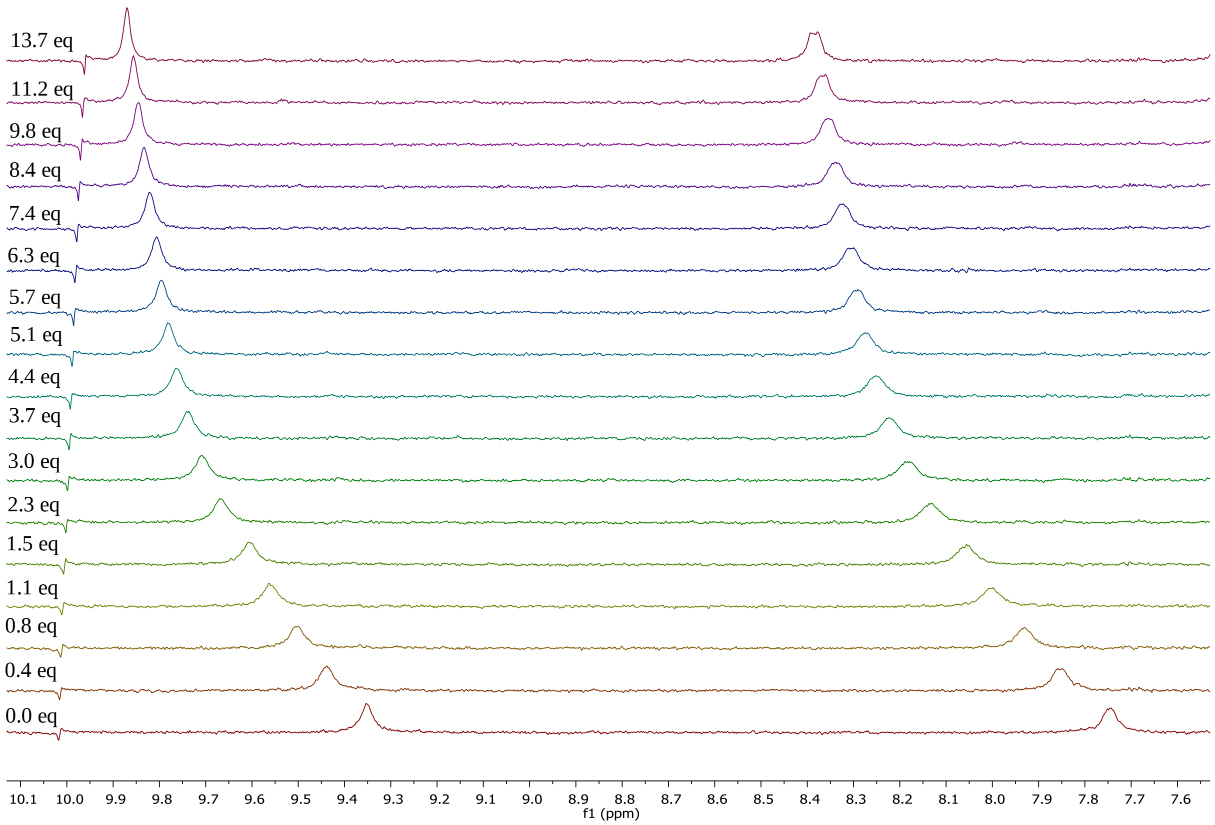

Figure S21. ^1H NMR spectra from the titration of $Bu_4N^+Cl^-$ into **7e** (1 mM) in DMSO-d_6/0.5% H_2O at 298 K. The amount of $Bu_4N^+Cl^-$ added is shown in equivalents relative to **7e**.

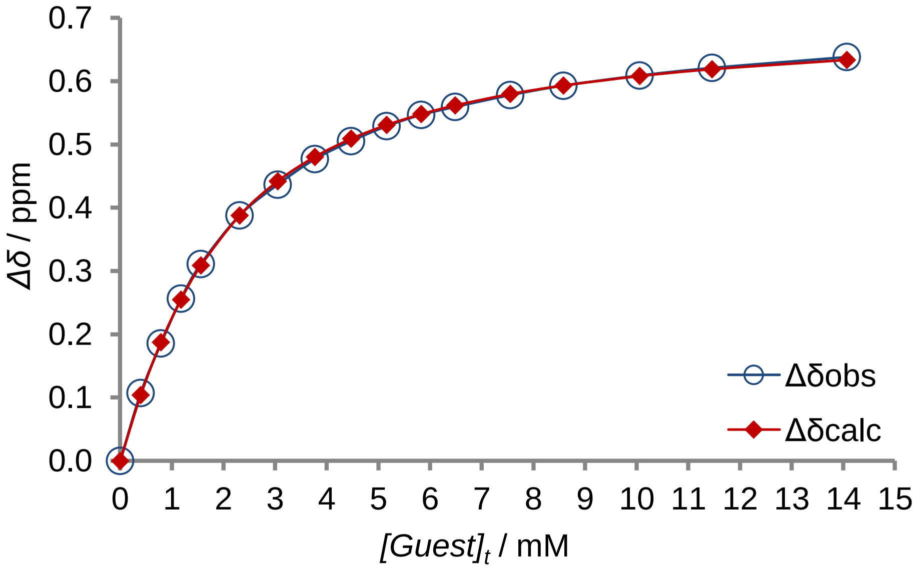

Figure S22. Graph showing the observed binding curve (red) and calculated fitting (blue) for **7e** when titrated against chloride at 298 K. The NH signal at δ = 7.75 ppm is plotted and both NH signals were used to find K_a = 708 ± 7 M^{-1}.

^1H NMR titration of 7f with Bu_4NCl in DMSO-d_6/0.5% H_2O

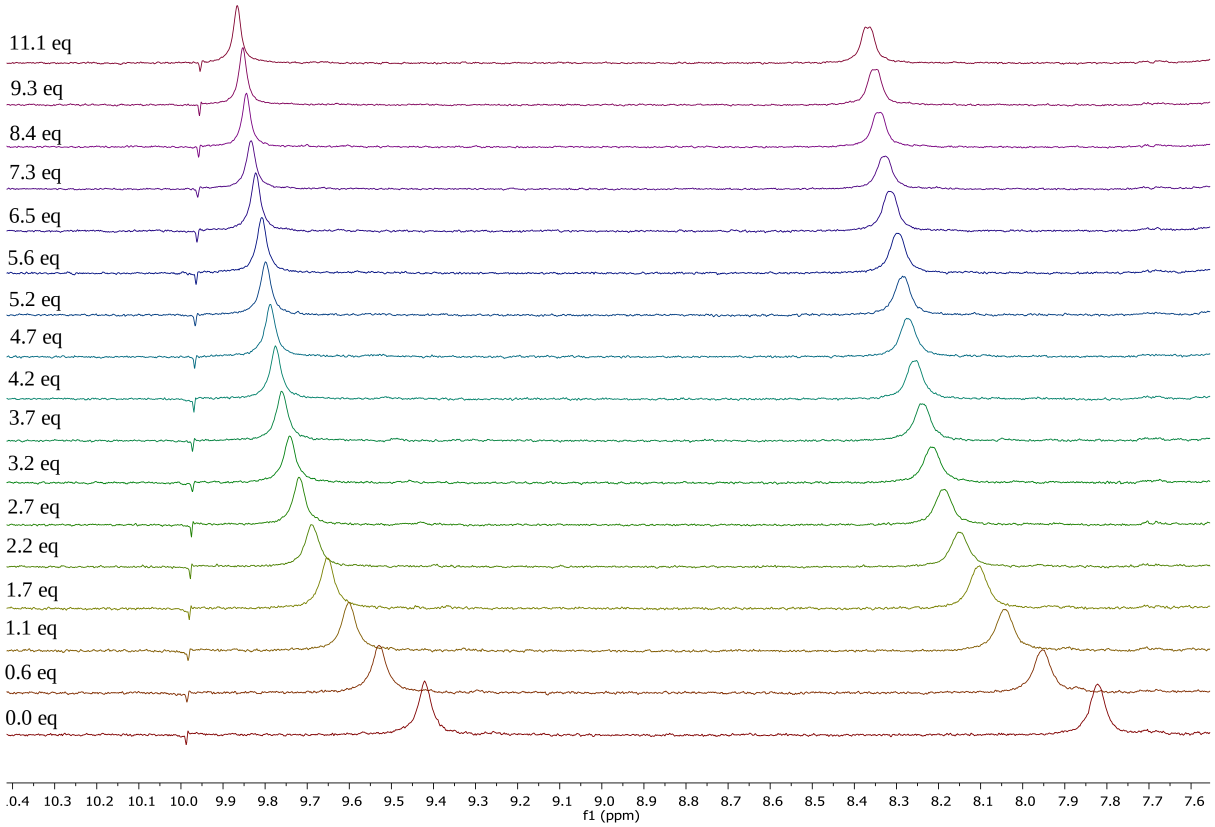

Figure S23. ^1H NMR spectra from the titration of $Bu_4N^+Cl^-$ into **7f** (1 mM) in DMSO-d_6/0.5% H_2O at 298 K. The amount of $Bu_4N^+Cl^-$ added is shown in equivalents relative to **7f**.

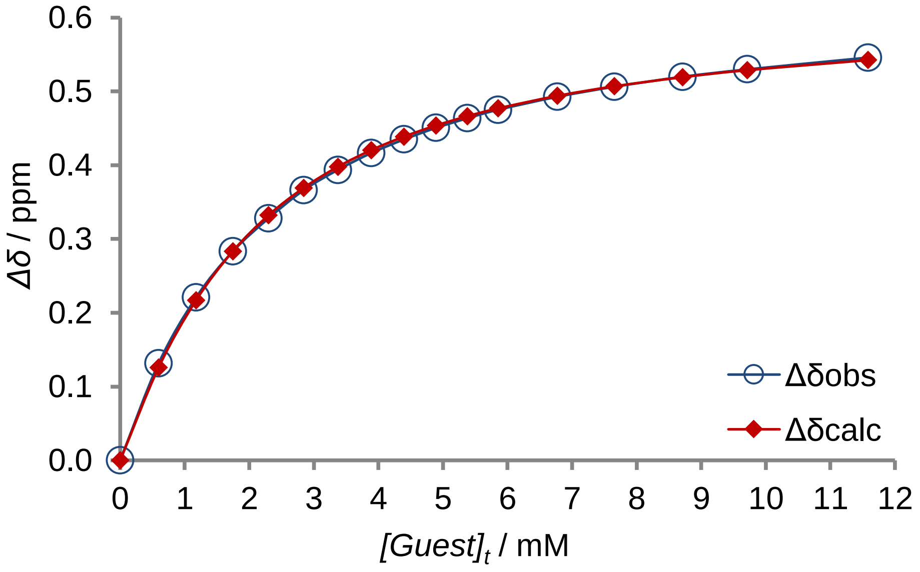

Figure S24. Graph showing the observed binding curve (red) and calculated fitting (blue) for **7f** when titrated against chloride at 298 K. The NH signal at δ = 7.82 ppm is plotted and both NH signals were used to find K_a = 667 ± 10 M^{-1}.

2.2 Chloride binding affinities in chloroform by Cram's extraction method[3,4]

General Procedure

$Et_4N^+Cl^-$ salt was obtained from commercial suppliers and dried overnight under high vacuum before use; receptors were also dried under high vacuum overnight before use. All host solutions were prepared using chloroform that had been deacidified by passage through a flash chromatography column containing activated basic alumina. Guest solutions were prepared using deionised water that had been passed through a Millipore filtration system.

A solution of host (~0.3 mM) in deacidified chloroform was prepared and a known volume (3 mL) was added to a 30 mL vial. To this organic solution was added a known volume (10 mL) of an aqueous solution of $Et_4N^+Cl^-$ (200, 300, or 400 mM in Millipore water). A magnetic stirring bar was added to the vial and the vial was immersed in a water bath that was heated to 303 K. The contents of the vial were stirred vigorously to get good mixing of the two phases. After 30 minutes stirring was stopped and the two phases were allowed to separate. The majority of the aqueous phase was removed using a pipette and the chloroform solution was filtered through Whatman 1PS hydrophobic filter paper to remove any trace of aqueous phase. The filtrate was concentrated *in vacuo* and the resulting solid was dried on a high vacuum line. The solid was then dissolved in acetone-d_6 and a ^{1}H NMR spectrum was collected at 298 K and referenced to the residual solvent peak (δ = 2.05 ppm). The CH_2 signal of $Et_4N^+Cl^-$ was integrated with respect to the receptor signals to give the guest:host ratio (*R*), allowing the value of K_a to be determined (see equations below).

The value for *R* obtained from the ^{1}H NMR spectra was used to calculate the equilibrium constant K_e:

$$H_{org} + X^-_{aq} + Y^+_{aq} \overset{K_e}{\rightleftharpoons} HX^-Y^+_{org} \qquad K_e = \frac{[HX^-Y^+]_{org}}{[H]_{org}[X^-]_{aq}[Y^+]_{aq}}$$

$$K_e = \frac{R}{(1-R)\left([G]^{initial}_{aq} - R\frac{V_{org}}{V_{aq}}[H]^{initial}_{org}\right)^2}$$

We have to take into account the equilibrium between the unbound $Et_4N^+X^-$ guest in the organic phase and the dissociated Et_4N^+ and X^- ions in the aqueous phase, described by:

$$X^-_{aq} + Y^+_{aq} \overset{K_d}{\rightleftharpoons} X^-Y^+_{org} \qquad K_d = \frac{[X^-Y^+]_{org}}{[X^-]_{aq}[Y^+]_{aq}}$$

The K_d value for $Et_4N^+Cl^-$has been determined and reported previously to be 1.269×10^{-5} M^{-1}.[3]

The binding constant K_a is then calculated from K_e and K_d :

$$H_{org} + X^{-}Y^{+}_{org} \overset{K_a}{\rightleftharpoons} HX^{-}Y^{+}_{org} \qquad K_a = \frac{[HX^{-}Y^{+}]_{org}}{[H]_{org}[X^{-}Y^{+}]_{org}} = \frac{K_e}{K_d}$$

Overview extraction experiments and K_a values for chloride

Table S1. Extraction data and derived association constants of decalins **7a-f** to $Et_4N^+Cl^-$ in $CHCl_3$.

Receptor	[H] (mM)	[G] (mM)	V_{org} (mL)	V_{aq} (mL)	R	K_a (M^{-1})
**7a**	0.29	200	3.0	10.0	0.198	4.9×10^5
	0.29	300	3.0	10.0	0.332	4.4×10^5
	0.29	400	3.0	10.0	0.435	3.7×10^5
					**Average:**	$\mathbf{4.3 \times 10^5}$
**7b**	0.28	200	3.0	10.0	0.073	1.5×10^5
	0.28	300	3.0	10.0	0.153	1.5×10^5
	0.28	400	3.0	10.0	0.280	1.8×10^5
					**Average:**	$\mathbf{1.6 \times 10^5}$
**7c**	0.30	200	3.0	10.0	0.087	1.9×10^5
	0.30	300	3.0	10.0	0.157	1.6×10^5
	0.30	400	3.0	10.0	0.219	1.4×10^5
					**Average:**	$\mathbf{1.6 \times 10^5}$
**7d**	0.28	200	3.0	10.0	0.078	1.7×10^5
	0.28	300	3.0	10.0	0.138	1.4×10^5
	0.28	400	3.0	10.0	0.220	1.4×10^5
					**Average:**	$\mathbf{1.5 \times 10^5}$
**7e**	0.28	200	3.0	10.0	0.088	1.9×10^5
	0.28	300	3.0	10.0	0.163	1.7×10^5
	0.28	400	3.0	10.0	0.243	1.6×10^5
					**Average:**	$\mathbf{1.7 \times 10^5}$
**7f**	0.30	200	3.0	10.0	0.090	1.9×10^5
	0.30	300	3.0	10.0	0.171	1.8×10^5
	0.30	400	3.0	10.0	0.255	1.7×10^5
					**Average:**	$\mathbf{1.8 \times 10^5}$

3. Transport Studies

3.1 General experimental description of transport measurements

Chloride ion transport was measured using large unilamellar vesicles (LUVs, 200 nm average diameter) composed of 1-palmitoyl-2-oleoyl-*sn*-glycero-3-phosphocholine (POPC) and cholesterol at a ratio of 7:3. POPC was obtained from Avanti® Polar Lipids, Inc. Extrusion apparatus and 200 nm polycarbonate membranes were obtained from GC Technology Ltd. The lipid solutions were prepared using chloroform that had been pH neutralised by passing the chloroform through a flash chromatography column containing basic alumina, and all aqueous solutions were prepared using deionised water that had been passed through a Millipore filtration system.

Compounds **7a-f** were studied at transporter:lipid ratios of 1:1000 and 1:2500.

The following is a typical example of an anion transport experiment:

A solution of the transporter in HPLC grade MeOH (35.9 µL, 2.4 nmol) was added to solutions of POPC (358 µL, 4.2 µmol) and cholesterol (214 µL, 1.8 µmol) in deacidified $CHCl_3$ to the give a transporter to lipid ratio of 1:2500. The solvents were evaporated under a gentle stream of N_2 and dried under high vacuum for 1 h. The resulting residue was hydrated with 500 µL of an aqueous solution of lucigenin (0.8 mM in 225 mM $NaNO_3$) and then sonicated for 30 s and stirred for 1 h to give heterogeneous LUVs. The multilamellar vesicles were disrupted by 10 freeze-thaw cycles and then the solution was carefully extruded (29 times) through a polycarbonate membrane (200 nm pore size) to give a uniform distribution of LUVs. The external lucigenin was removed by passing the solution through a size exclusion column (~ 2 g Sephadex 50G, eluted in $NaNO_3$ 225 mM) and the collected vesicles were diluted to a volume of 15 mL (0.4 mM) with $NaNO_3$ solution (225 mM).

The vesicle solution (3.00 mL) was transferred into a quartz cuvette and the fluorescence was monitored over 900 s at 25 °C using an excitation wavelength of 450 nm and an emission wavelength of 535 nm on a Perkin Elmer LS45 Fluorescence spectrometer. NaCl (75 µL, 1.0 M in 225 mM $NaNO_3$) was added to the vesicles 30 seconds after the start of the experiment. Every measurement was repeated 3-4 times. The first 30 seconds and the initial drop (due to quenching of external lucigenin) were removed, the traces were normalised and the resulting F/F_0 traces were averaged. The traces thus obtained from the transport measurements at transporter:lipid ratios of 1:1000 are given in Figure 1a of the main text and traces at 1:2500 are shown in Figure S25.

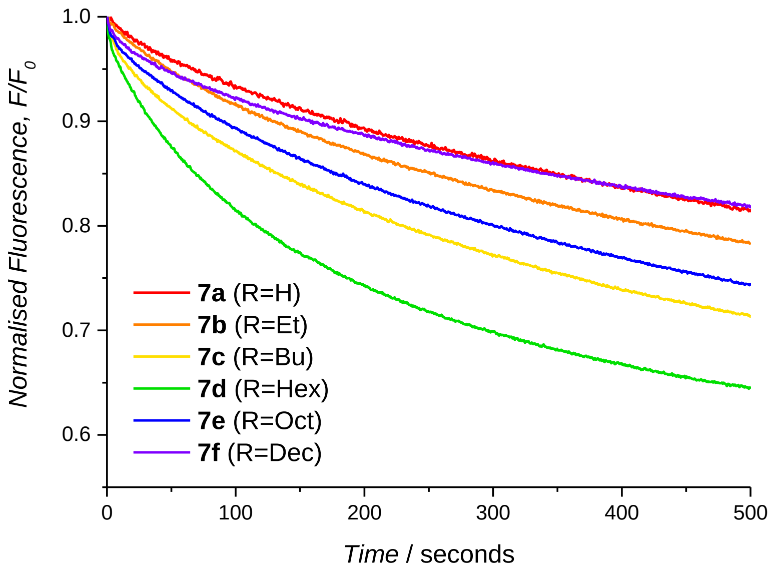

Figure S25. Chloride transport into 200 nm vesicles by decalins **7a-f** at transporter:lipid ratios of 1:2500.

3.2 Fitting of transport data

Half-lives of fluorescence decay were obtained by fitting the reciprocal transport curves (F_0/F) from 0-500 seconds to a single exponential decay function (Eq. 1) using Origin 9.0. The half-life $t_{½}$ was calculated using fit parameter 'b' (Eq. 2).

(1) $$\frac{F_0}{F} = y + ae^{-bt}$$

(2) $$t_{1/2} = \frac{\ln(2)}{b}$$

Initial rates of fluorescence decay were obtained by fitting the reciprocal transport curves (F_0/F) from 0-500 seconds to a double exponential decay function (Eq. 3) using Origin 9.0. Differentiating Eq. 3 at t = 0 gives the initial rate I (Eq. 4).

(3) $$\frac{F_0}{F} = y - ae^{-bt} - ce^{-dt}$$

(4) $$I = ab + cd$$

Specific initial rates [I] are independent of the transporter:lipid ratio and calculated by dividing initial rates I by the transporter:lipid ratio, and then averaging the obtained values for all transporter:lipid ratios studied.[5]

All parameters obtained from fitting the transport data are summarised in Table S2.

Table S2. Half-lives, initial rates, and specific initial rates of transport for decalin compounds **7a-f**.

**Compound**	**R**	**Half-life (s)**		**Initial rate (s^{-1})**		**Specific initial rate [I] (s^{-1})**
		1:2500	*1:1000*	*1:2500*	*1:1000*	
**7a**	H	383	311	0.0010	0.0026	2.6
**7b**	Ethyl	318	266	0.0012	0.0033	3.2
**7c**	*n*-Butyl	305	223	0.0027	0.0043	5.5
**7d**	*n*-Hexyl	196	147	0.0031	0.0068	7.3
**7e**	*n*-Octyl	345	230	0.0018	0.0034	4.0
**7f**	*n*-Decyl	305	289	0.0015	0.0024	3.1

3.3 Test for leaching of decalin 7a from vesicle membranes

Decalin **7a** was pre-incorporated into vesicles (7 µmol cholesterol, 3 µmol cholesterol, containing 0.8 mM lucigenin and 225 mM $NaNO_3$) at a 1:2500 transporter to lipid ratio as previously described, the final step being size exclusion chromatography over Sephadex 50G. The resulting suspension of vesicles was diluted to a volume of 5 mL with 225 mM $NaNO_3$ to give a 2.0 mM lipid solution. 3.0 mL of this solution was diluted to a volume of 15 mL with 225 mM $NaNO_3$ to give a 0.4 mM lipid solution, and aliquots of this solution were further diluted to give a 0.2 mM lipid solution and a 0.1 mM lipid solution. The remaining 2.0 mL of the 2.0 mM solution was passed through a size exclusion column for a second time and then diluted to a concentration of 0.4 mM (in 225 mM $NaNO_3$). The decay in florescence on addition of NaCl to these four vesicle solutions is shown in Figure S26.

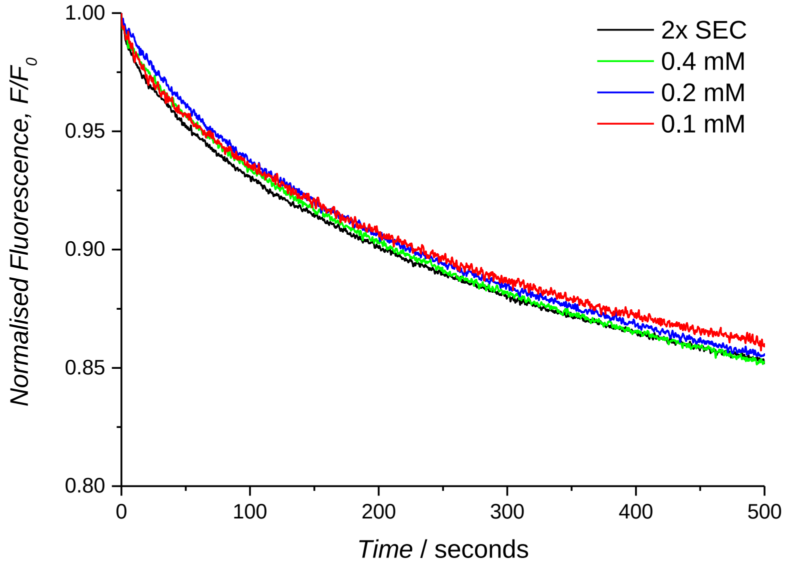

Figure S26. Average fluorescence traces for chloride transport by decalin **7a** at varying vesicle concentrations (green = 0.4 mM, blue = 0.2 mM, red = 0.1 mM) and with multiple runs through a size exclusion column (black).

If the transporter were capable of leaching from the membrane into the aqueous phase, both dilution with water and the additional passage through Sephadex would be expected to lower the concentration in the membrane (in the former case by increasing the volume of the aqueous reservoir, in the latter by removing transporter dissolved in water). Neither treatment caused a significant change in the rate of chloride transport. Therefore, we conclude that **7a** does not leach from the membrane into the aqueous phase and that the lower rates of transport by **7a** with respect to **7d** are not caused by a lower amount of transporter present in the membranes during the measurements.[6]

4. Molecular Dynamics Simulations

4.1 Computational Methods

The MD simulations were carried out with the AMBER 14 software suite,[7] using the LIPID14 force field[8] for the POPC lipids and the GAFF[9] parameters and atomic RESP charges[10] for transporters **7a-f** as follows.

a) Calculation of atomic RESP charges for transporters 7a-7f

The initial structure of **7a** was generated from a crystal structure of a decalin derivative featuring a carboxylate group at C4a position.[11] Two phenyl thiourea substituents were appended at both C2 and C7 decalin skeleton positions. The remaining five molecules of the diaxial thiourea series were generated from **7a** by addition of the appropriate alkyl chain at the *para* position of both phenyl rings. Subsequently, all structures were optimized at the HF/6-31G* level with the Gaussian 09 software.[12] The optimization of each single structure of **7a-7f** was followed by a single point calculation to generate the electrostatic potential (ESP) at the same theory level, using the Merz-Singh-Kollman scheme with 4 concentric layers per atom and 6 density points in each layer (IOp(6/33=2, 6/41=4, 6/42=6)). The initial atomic charges of each molecule were then calculated by RESP fitting, along with the attribution of GAFF atom types, using the antechamber module, as implemented in the AMBER software suite.[7a]

To obtain atomic charges less dependent of the molecular conformation or orientation, the calculation of the final RESP charges employed in the MD simulations reported was preceded by conformational analyses on receptors **7a-f**. The initial molecular mechanics (MM) energy minimized structures, using the initial RESP charges, were heated in the gas phase at 500 K for 50 ps, followed by a collection run of 5 ns, using a time step of 1 fs. The use of this high temperature allows the stochastic searching of conformational space, since the energetic barriers are easily surmounted.[13] Frames were saved every 0.1 ps leading to a trajectory file containing 50000 structures for each molecule. All these structures were further minimized by MM using a steepest descendent gradient followed by the conjugate gradient algorithm, until the convergence criterion of 0.0001 kcal mol^{-1} $Å^{-1}$ was attained. Afterwards, the MM minimised structures were clustered with the UCSF Chimera software.[14] Five representative conformations with substantially different RMSD values with both thiourea binding units adopting a *syn* configuration but displaying a different spatial disposition were selected and underwent new HF/6-31G* geometry optimizations and ESP calculations as described above. The individual ESP data were extracted from the corresponding Gaussian 09[12] output and then the ESP data of the five conformations were concatenated and subsequently used to generate the input files for the two-stage RESP fitting, using identical weights for all conformations.

b) Generation of chloride complexes

The structures of **7a-7f** chloride complexes were obtained in gas phase *via* conformational analyses as described above for the free molecules. However, the production runs were carried out only for 1 ns, leading at the end the quenched dynamics protocol to 10000 MM minimized structures. The most representative structure of each complex, obtained by cluster analysis with UCSF Chimera software,[8],was selected for the subsequent MD simulations (Figure S27).

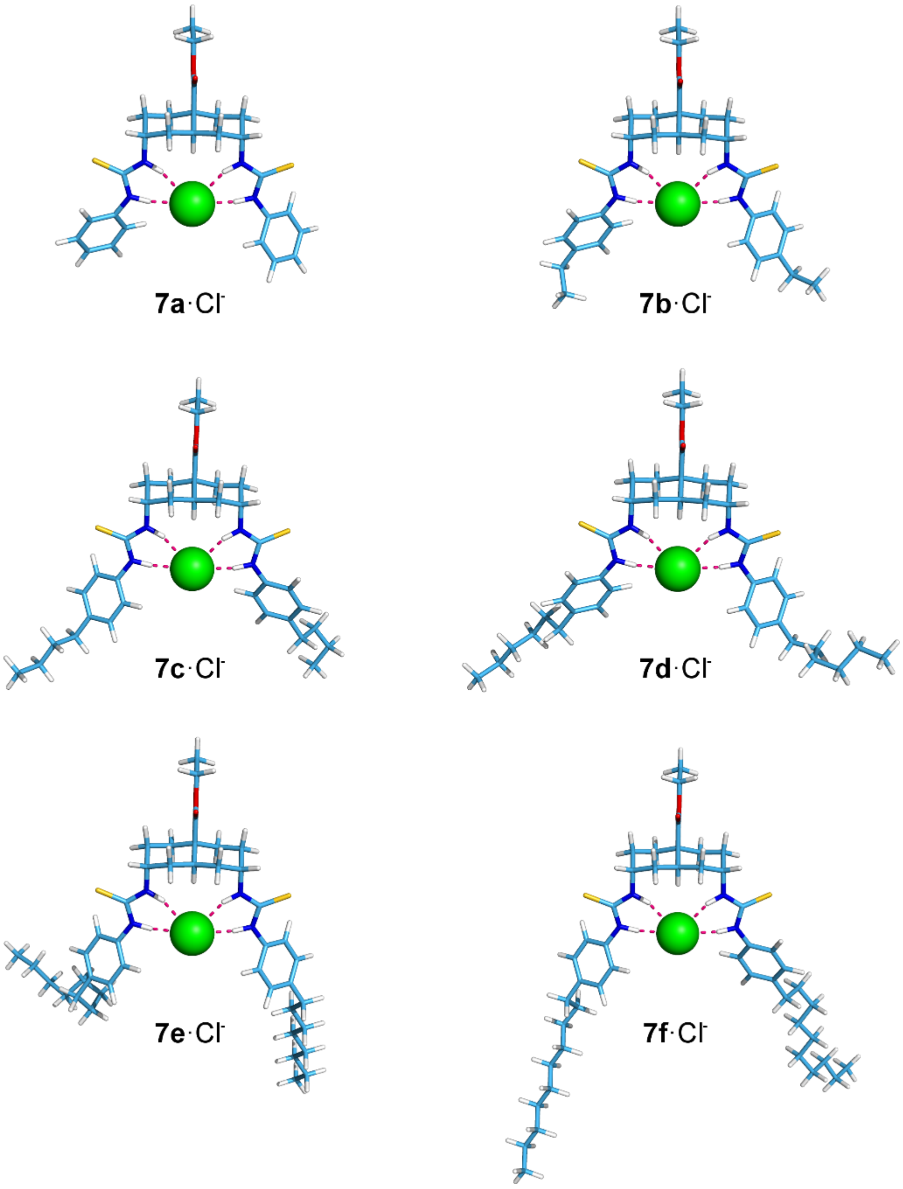

Figure S27. Chloride complexes of **7a-7f** obtained from quenched molecular dynamics simulations. The transporter is shown in sticks, with the hydrogen atoms shown in white, oxygen atoms in red, nitrogen atoms in blue, sulfur atoms in yellow, and carbon atoms in light blue. The chloride is shown as a green sphere and the N-H···Cl^- bonds are shown as magenta dashes.

c) Simulations in a POPC bilayer

i. Simulation of the initial POPC bilayer

Before the simulation of the chloride complexes inserted into a POPC membrane model, a membrane system with a orthorhombic shape (dubbed *free membrane*) composed of 128 phospholipids, 6500 TIP3P water molecules, 18 Cl^- and 18 Na^+ ions (0.15 M) was built with a water per lipid ratio of 50.1. This free membrane was simulated for 150 ns, under periodic boundary conditions at 303 K with force field parameters taken from LIPID14 and the ions described with van der Waals parameters developed to be used along with the TIP3P water model.[15] The simulation protocol followed is equivalent to the one detailed below for the membrane systems with chloride complexes. The structural parameters area per lipid, bilayer thickness, order parameters and electron density profiles estimated for the last 70 ns of sampling, are similar to those originally reported for the POPC bilayer system containing a water per lipid ratio of 31.6.[16] Therefore, increasing the number water molecules had no impact in the bilayer system, and this equilibrated *free membrane* was subsequently used in the MD simulations carried out with the chloride complexes either inserted into the core of the bilayer (setup A – an approximation to the experimental chloride transmembrane transport studies) or immersed in the water slab (setup B). This initial positioning of the chloride complexes of **7a-7f** within the free membrane was performed with Packmol,[17] and yielded systems A.**7a** to A.**7f**, as well as systems B.**7a** to B.**7f**, as given in Table S3.

ii. Simulation of membrane systems with chloride complexes at the bilayer core – setup A

The MD simulations A_1.**7a** to A_4.**7f** were carried out as follows: the initial configuration of each system was submitted to 10000 steps of MM energy minimization with a 500 kcal/mol $Å^2$ positional restraint on the chloride complex and lipid molecules, through the steepest descent algorithm for 3000 steps plus 7000 steps of the conjugated gradient algorithm, followed by the relaxation of the entire system for another 10000 steps, with the same protocol. The equilibration of the system proceeded by heating it to 303 K in an NVT ensemble for 100 ps with a 10 kcal/mol $Å^2$ restraint on the chloride complex and lipid molecules. The equilibration stage proceeded with a 5 ns run using an NPT ensemble with a 5 kcal/mol $Å^2$ restraint on the chloride complex. Then, the positional restraint was removed and the simulation continued for further 150 ns. The long-range electrostatic interactions were described with the Particle Mesh Ewald (PME) algorithm[18] using a real-space cut-off at 10 Å. The cut-off for the Lennard-Jones interactions was also set at 10 Å. The temperature of the system was maintained independently by coupling the system to an external bath temperature of 303 K, using the Langevin thermostat,[19] and a coupling constant τ_T of 1.0 ps. The pressure was controlled by the Berendsen barostat[20] at 1 atm and compressibility of 44.6×10^{-6} bar^{-1}, with a coupling constant τ_P of 1.0 ps. The covalent bonds to hydrogen atoms were constrained using the SHAKE algorithm,[21] allowing the use of a 2 fs time step. This protocol

was employed to run four independent replicates of 150 ns of setup A membrane systems (Table S3), with the frames saved every 10.0 ps.

iii. Simulation of membrane systems with chloride complexes at the bilayer core – setup A'

From the end of the equilibration period of simulations A_1.**7a**-A_1.**7f**, a single production run of 150 ns was undertaken with each chloride complex maintained through the use of four distance restraints between the nitrogen atoms of each thiourea binding units and the chloride. Harmonic restraints of 5 kcal/mol were applied to obtain N···Cl^- distances of 3.5 Å.

iv. Simulation of membrane systems with chloride complexes in the water slab – setup B

The MD simulations B_1.**7a**-B_2.**7f**, listed in Table S3, were carried out using the aforementioned protocol given for simulations of setup A, except for the length of the production runs, which were 200 ns long.

Table S3. Membrane systems simulated at 303 K.

Setup	Transporter	System ID	Simulation ID Rep. 1	Rep. 2	Rep. 3	Rep. 4	Initial Position	Simulation time (ns)	N···Cl^- distance restraints
A	**7a**	A.**7a**	A_1.**7a**	A_2.**7a**	A_3.**7a**	A_4.**7a**	Bilayer core	150	No
A	**7b**	A.**7b**	A_1.**7b**	A_2.**7b**	A_3.**7b**	A_4.**7b**	Bilayer core	150	No
A	**7c**	A.**7c**	A_1.**7c**	A_2.**7c**	A_3.**7c**	A_4.**7c**	Bilayer core	150	No
A	**7d**	A.**7d**	A_1.**7d**	A_2.**7d**	A_3.**7d**	A_4.**7d**	Bilayer core	150	No
A	**7e**	A.**7e**	A_1.**7e**	A_2.**7e**	A_3.**7e**	A_4.**7e**	Bilayer core	150	No
A	**7f**	A.**7f**	A_1.**7f**	A_2.**7f**	A_3.**7f**	A_4.**7f**	Bilayer core	150	No
A'	**7a**	A'.**7a**	A'_1.**7a**	–	–	–	Bilayer core	150	Yes
A'	**7b**	A'.**7b**	A'_1.**7b**	–	–	–	Bilayer core	150	Yes
A'	**7c**	A'.**7c**	A'_1.**7c**	–	–	–	Bilayer core	150	Yes
A'	**7d**	A'.**7d**	A'_1.**7d**	–	–	–	Bilayer core	150	Yes
A'	**7e**	A'.**7e**	A'_1.**7e**	–	–	–	Bilayer core	150	Yes
A'	**7f**	A'.**7f**	A'_1.**7f**	–	–	–	Bilayer core	150	Yes
B	**7a**	B.**7a**	B_1.**7a**	B_2.**7a**	–	–	Water phase	200	No
B	**7b**	B.**7b**	B_1.**7b**	B_2.**7b**	–	–	Water phase	200	No
B	**7c**	B.**7c**	B_1.**7c**	B_2.**7c**	–	–	Water phase	200	No
B	**7d**	B.**7d**	B_1.**7d**	B_2.**7d**	–	–	Water phase	200	No
B	**7e**	B.**7e**	B_1.**7e**	B_2.**7e**	–	–	Water phase	200	No
B	**7f**	B.**7f**	B_1.**7f**	B_2.**7f**	–	–	Water phase	200	No

4.2 Structural impact of 7a-f on the bilayer model

The structural impact of **7a-f** in the membrane model was examined through evaluation of the area per lipid, bilayer thickness, electron density profiles and order parameters $|S_{CD}|$ during the last 50 ns of each MD replicate of setup A with each transporter (see Table S3). The average values for area per lipid and bilayer thickness are gathered in Table S4 and the electron density profiles and order parameters are plotted in Figures Figure **S28** to Figure **S39**. The area per lipid and bilayer thickness average values are quite similar to the ones reported for the membrane system without any transporter. The small variations observed in all these structural parameters can be attributed to the necessary adjustments for the nesting of the transporters at the interface level.

Table S4. Comparison between the area per lipid and bilayer thickness of simulations A_1.**7a**-A_4.**7f** (50 ns of sampling) to the free membrane system (70 ns of sampling).

Simulation ID	Area per lipid (Å^2)		Bilayer thickness (Å^2)		*N*
	Avg ± SD	Range	Avg ± SD	Range	
Free membrane	65.58 ± 1.19	[61.98:68.98]	37.75 ± 0.57	[36.05:39.60]	7000
A_1.**7a**	66.26 ± 1.12	[63.22:69.29]	37.50 ± 0.52	[36.28:39.22]	5000
A_2.**7a**	65.04 ± 1.34	[62.00:68.51]	38.15 ± 0.68	[36.57:40.02]	5000
A_3.**7a**	66.19 ± 0.93	[63.16:68.80]	37.57 ± 0.46	[36.21:38.95]	5000
A_4.**7a**	65.38 ± 0.84	[62.77:68.21]	37.93 ± 0.47	[36.40:39.48]	5000
A_1.**7b**	66.07 ± 0.86	[63.64:68.98]	37.69 ± 0.39	[36.22:38.73]	5000
A_2.**7b**	65.68 ± 1.26	[61.21:69.21]	37.84 ± 0.56	[36.44:40.09]	5000
A_3.**7b**	66.04 ± 1.00	[63.12:68.31]	37.65 ± 0.47	[36.46:39.26]	5000
A_4.**7b**	66.10 ± 1.00	[63.34:68.96]	37.66 ± 0.42	[36.46:38.78]	5000
A_1.**7c**	66.76 ± 1.67	[63.11:70.61]	37.39 ± 0.75	[35.47:39.33]	5000
A_2.**7c**	65.48 ± 0.93	[62.66:68.40]	37.98 ± 0.45	[36.49:39.45]	5000
A_3.**7c**	65.73 ± 1.45	[61.95:69.61]	37.90 ± 0.70	[36.02:39.82]	5000
A_4.**7c**	66.40 ± 1.15	[63.47:70.31]	37.61 ± 0.55	[35.94:38.99]	5000
A_1.**7d**	65.66 ± 1.34	[62.69:69.17]	37.88 ± 0.71	[35.87:39.79]	5000
A_2.**7d**	65.65 ± 1.19	[62.10:70.29]	37.88 ± 0.56	[35.79:39.77]	5000
A_3.**7d**	65.59 ± 1.23	[62.05:68.73]	38.01 ± 0.52	[36.60:39.49]	5000
A_4.**7d**	65.39 ± 1.01	[62.55:68.42]	37.98 ± 0.50	[36.59:39.55]	5000
A_1.**7e**	65.76 ± 1.00	[63.11:68.80]	37.92 ± 0.52	[36.27:39.47]	5000
A_2.**7e**	66.00 ± 0.97	[63.78:69.30]	37.82 ± 0.50	[36.20:39.19]	5000
A_3.**7e**	65.37 ± 1.20	[61.96:69.07]	38.05 ± 0.58	[36.32:39.91]	5000
A_4.**7e**	66.52 ± 1.13	[63.49:69.80]	37.54 ± 0.50	[36.13:38.93]	5000
A_1.**7f**	66.08 ± 1.27	[63.18:69.30]	37.77 ± 0.58	[36.22:39.36]	5000
A_2.**7f**	66.03 ± 1.45	[62.65:70.70]	37.71 ± 0.64	[35.86:39.46]	5000
A_3.**7f**	65.17 ± 1.09	[61.97:67.87]	38.16 ± 0.51	[36.79:39.61]	5000
A_4.**7f**	65.34 ± 0.97	[62.90:68.36]	38.09 ± 0.44	[36.54:39.34]	5000

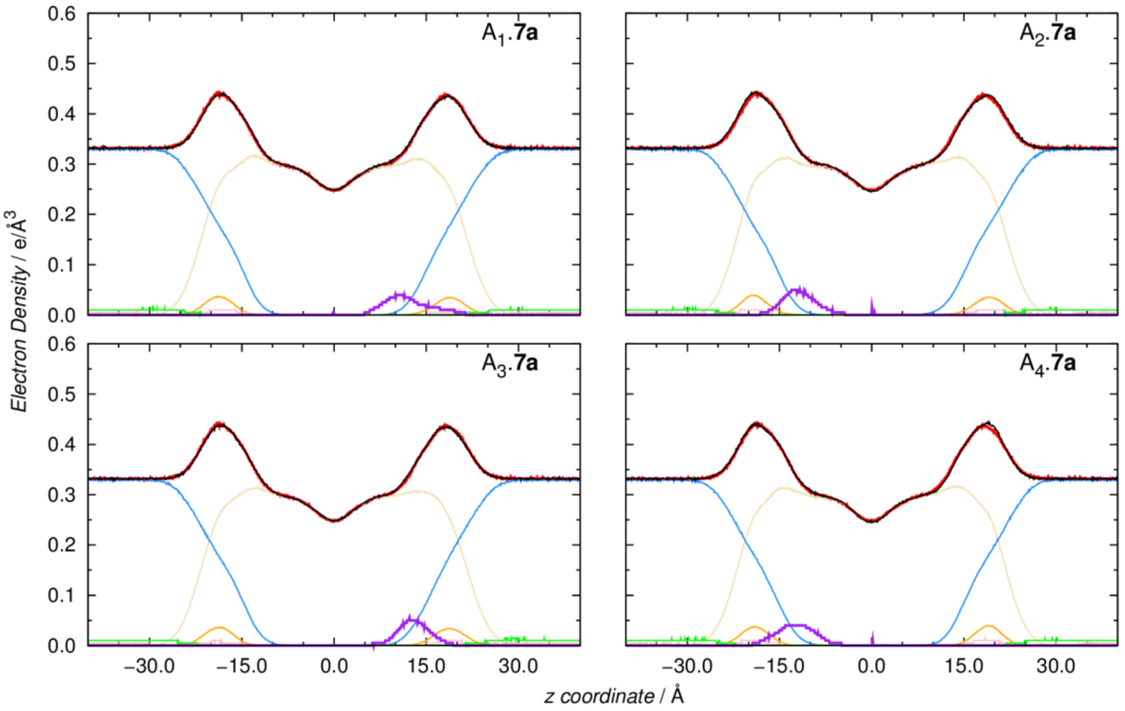

Figure S28. Electron density profiles of simulations A_1.**7a**-A_4.**7a** with the full system plotted in black, water in blue, phospholipids in wheat, phosphorus atoms in orange, chloride ions in green, sodium atoms in pink and **7a** in purple. The ions and the transporter are scaled 5 times. The z = 0 Å corresponds to the core of the POPC bilayer. The free membrane profile is also shown as a red line.

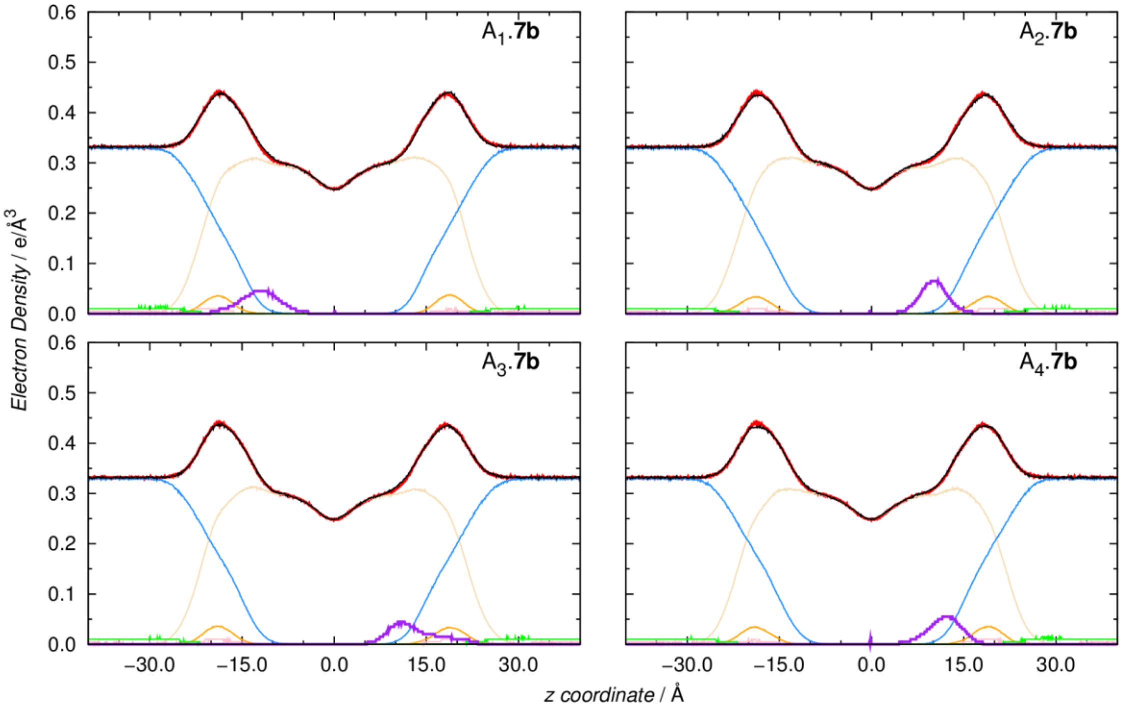

Figure S29. Electron density profiles of simulations A_1.**7b**-A_4.**7b** with the full system plotted in black, water in blue, phospholipids in wheat, phosphorus atoms in orange, chloride ions in green, sodium atoms in pink and **7b** in purple. The ions and the transporter are scaled 5 times. The z = 0 Å corresponds to the core of the POPC bilayer. The free membrane profile is also shown as a red line.

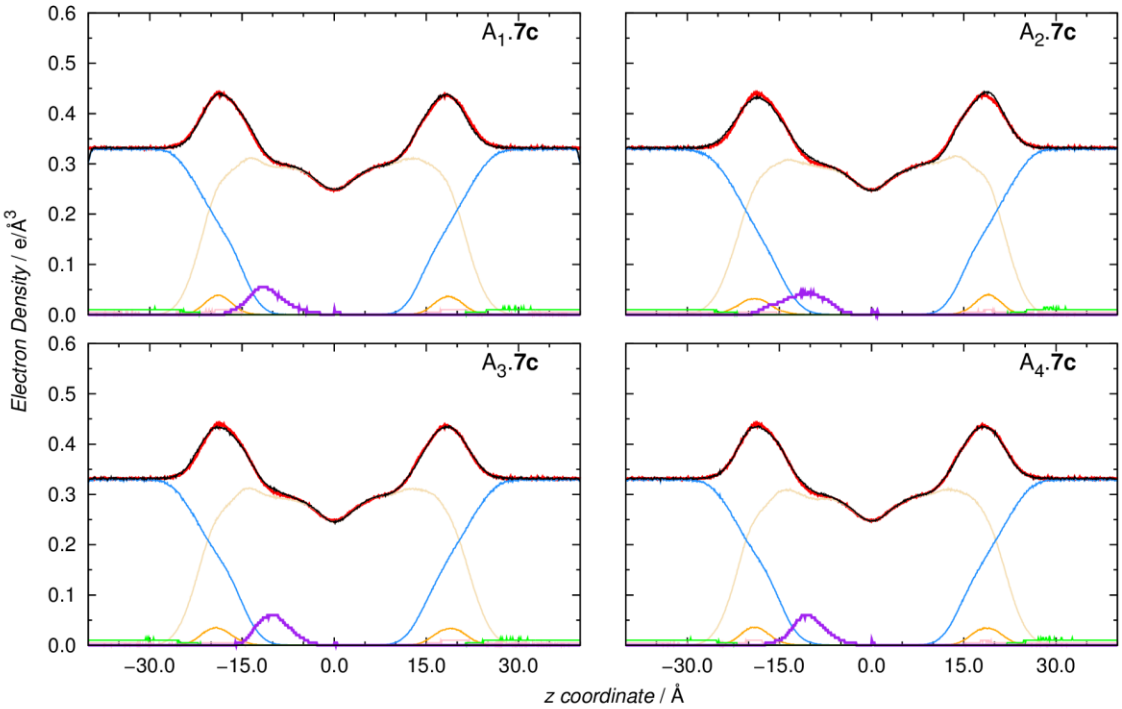

Figure S30. Electron density profiles of simulations A_1.**7c**-A_4.**7c** with the full system plotted in black, water in blue, phospholipids in wheat, phosphorus atoms in orange, chloride ions in green, sodium atoms in pink and **7c** in purple. The ions and the transporter are scaled 5 times. The z = 0 Å corresponds to the core of the POPC bilayer. The free membrane profile is also shown as a red line.

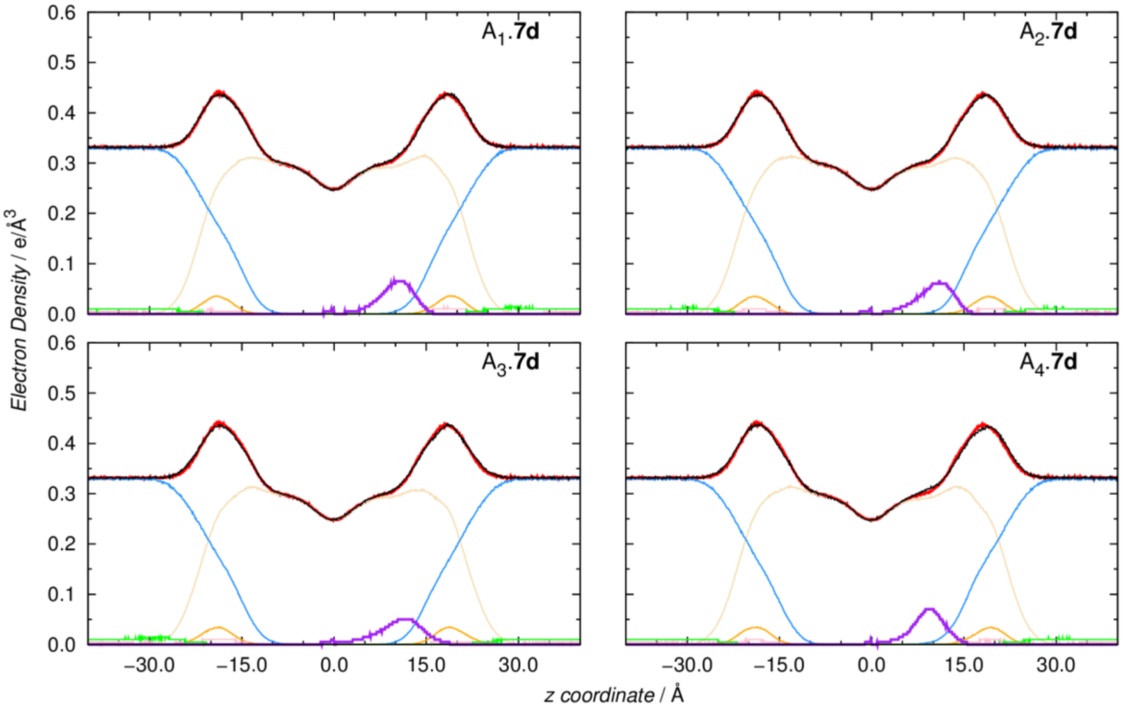

Figure S31. Electron density profiles of simulations A_1.**7d**-A_4.**7d** with the full system plotted in black, water in blue, phospholipids in wheat, phosphorus atoms in orange, chloride ions in green, sodium atoms in pink and **7d** in purple. The ions and the transporter are scaled 5 times. The z = 0 Å corresponds to the core of the POPC bilayer. The free membrane profile is also shown as a red line.

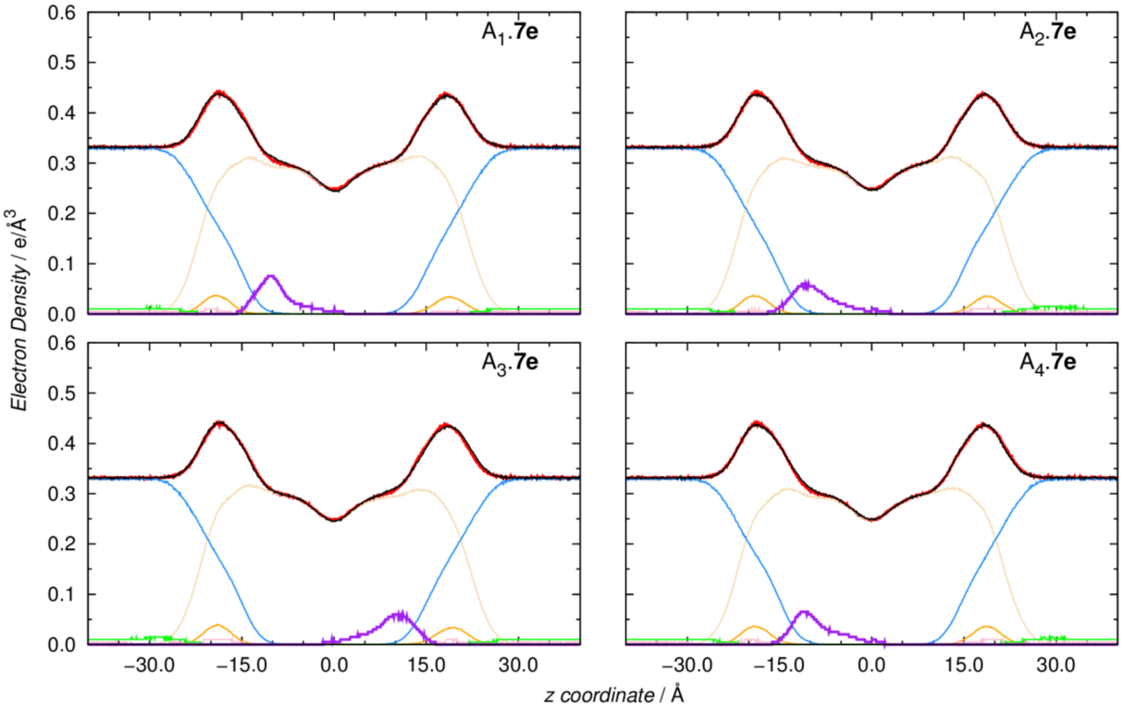

Figure S32. Electron density profiles of simulations A_1.**7e**-A_4.**7e** with the full system plotted in black, water in blue, phospholipids in wheat, phosphorus atoms in orange, chloride ions in green, sodium atoms in pink and **7e** in purple. The ions and the transporter are scaled 5 times. The z = 0 Å corresponds to the core of the POPC bilayer. The free membrane profile is also shown as a red line.

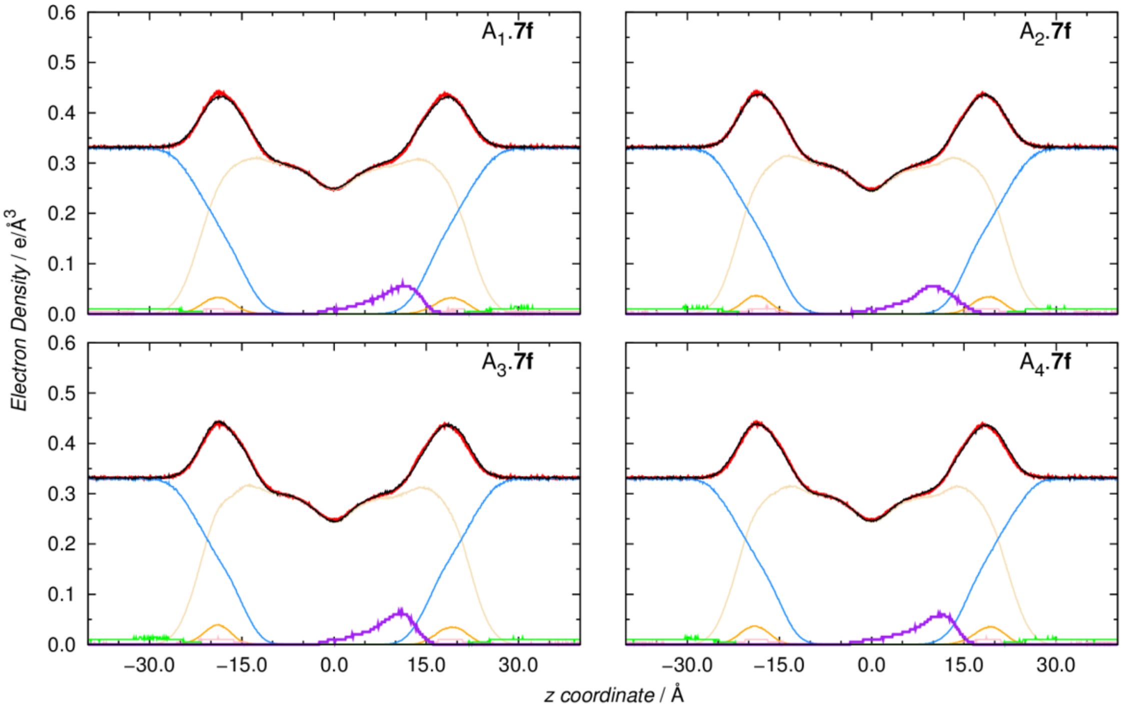

Figure S33. Electron density profiles of simulations A_1.**7f**-A_4.**7f** with the full system plotted in black, water in blue, phospholipids in wheat, phosphorus atoms in orange, chloride ions in green, sodium atoms in pink and **7f** in purple. The ions and the transporter are scaled 5 times. The z = 0 Å corresponds to the core of the POPC bilayer. The free membrane profile is also shown as a red line.

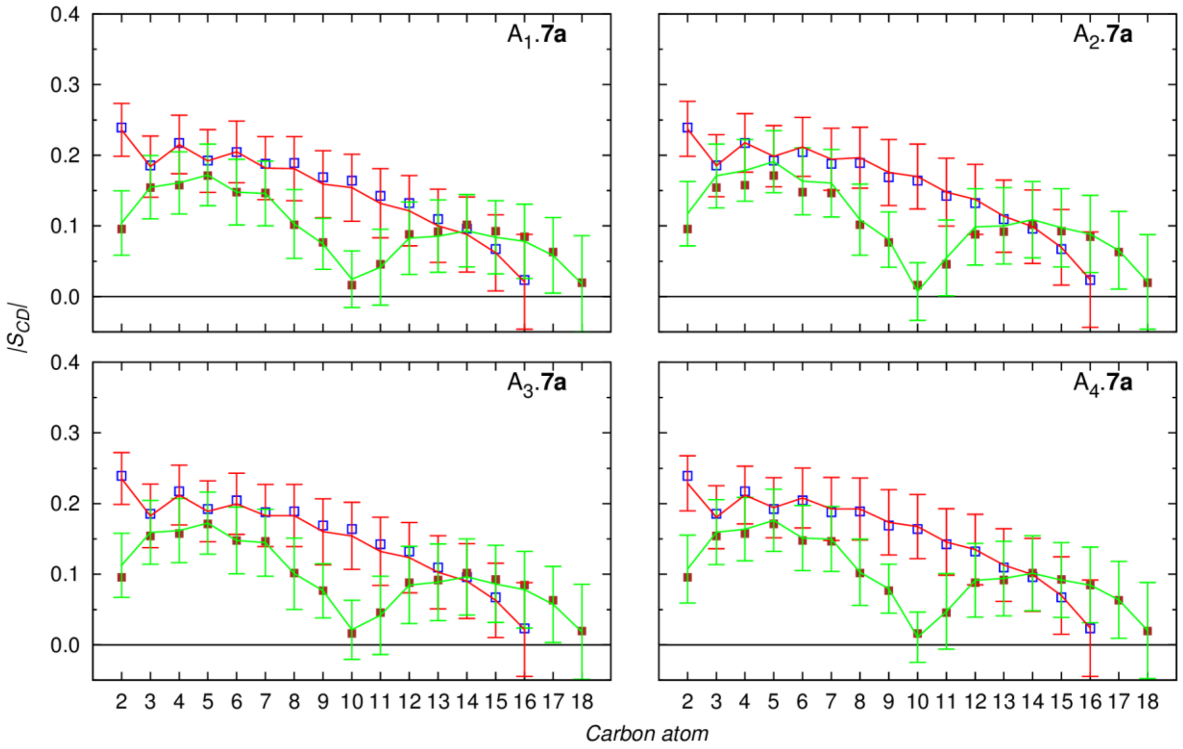

Figure S34. Computed $|S_{CD}|$ for the palmitoyl and oleyl chains for the last 50 ns of MD simulation runs A_1.**7a**-A_4.**7a**. The $|S_{CD}|$ values calculated for the sn-1 chain are shown in red, while the values for the sn-2 chain are shown in green. The error bars associated with these results correspond to the SD. The computed $|S_{CD}|$ values from the free membrane are presented as blue open squares (sn-1 chain), and brown filled squares (sn-2 chain).

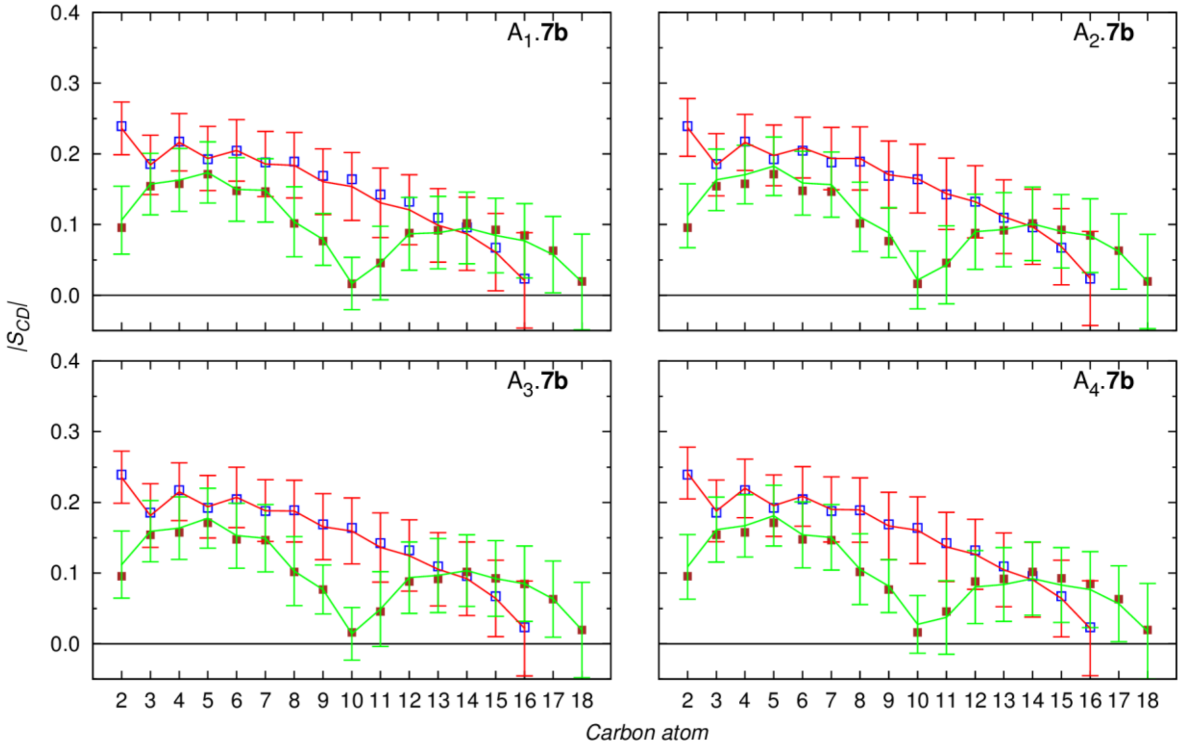

Figure S35. Computed $|S_{CD}|$ for the palmitoyl and oleyl chains for the last 50 ns of MD simulation runs A_1.**7b**-A_4.**7b**. The $|S_{CD}|$ values calculated for the sn-1 chain are shown in red, while the values for the sn-2 chain are shown in green. The error bars associated with these results correspond to the SD. The computed $|S_{CD}|$ values from the free membrane are presented as blue open squares (sn-1 chain), and brown filled squares (sn-2 chain).

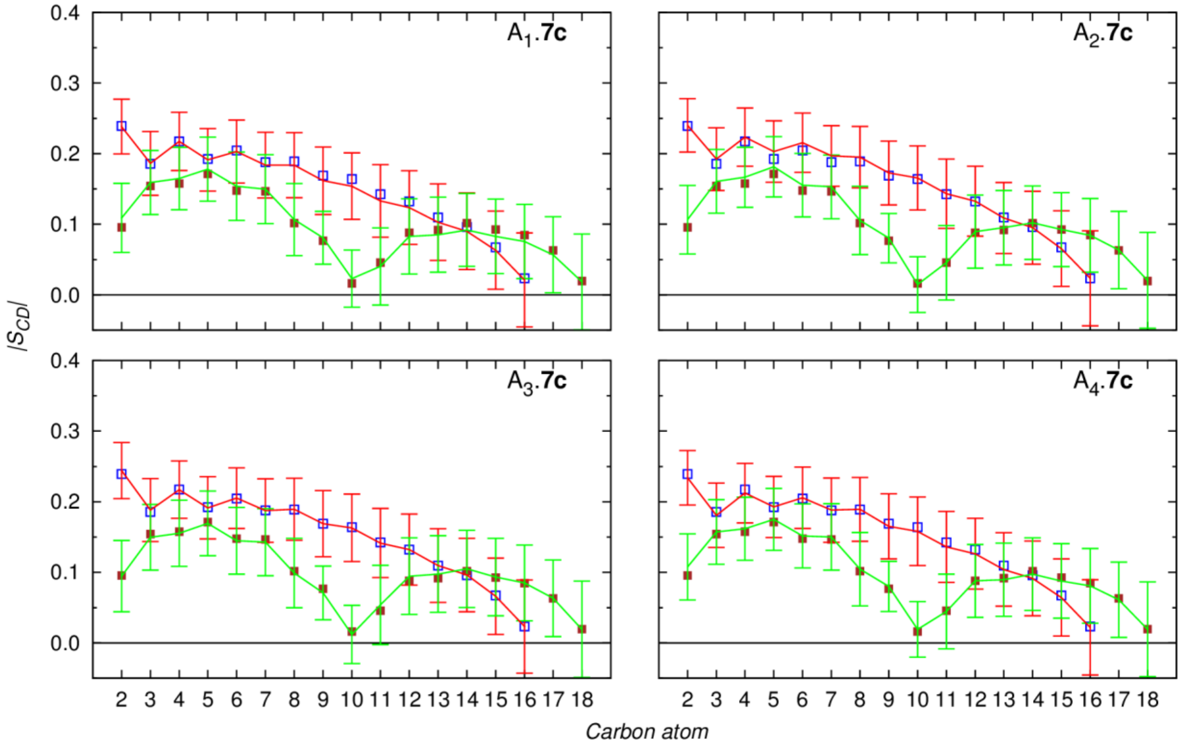

Figure S36. Computed $|S_{CD}|$ for the palmitoyl and oleyl chains for the last 50 ns of MD simulation runs A_1.**7c**-A_4.**7c**. The $|S_{CD}|$ values calculated for the sn-1 chain are shown in red, while the values for the sn-2 chain are shown in green. The error bars associated with these results correspond to the SD. The computed $|S_{CD}|$ values from the free membrane are presented as blue open squares (sn-1 chain), and brown filled squares (sn-2 chain).

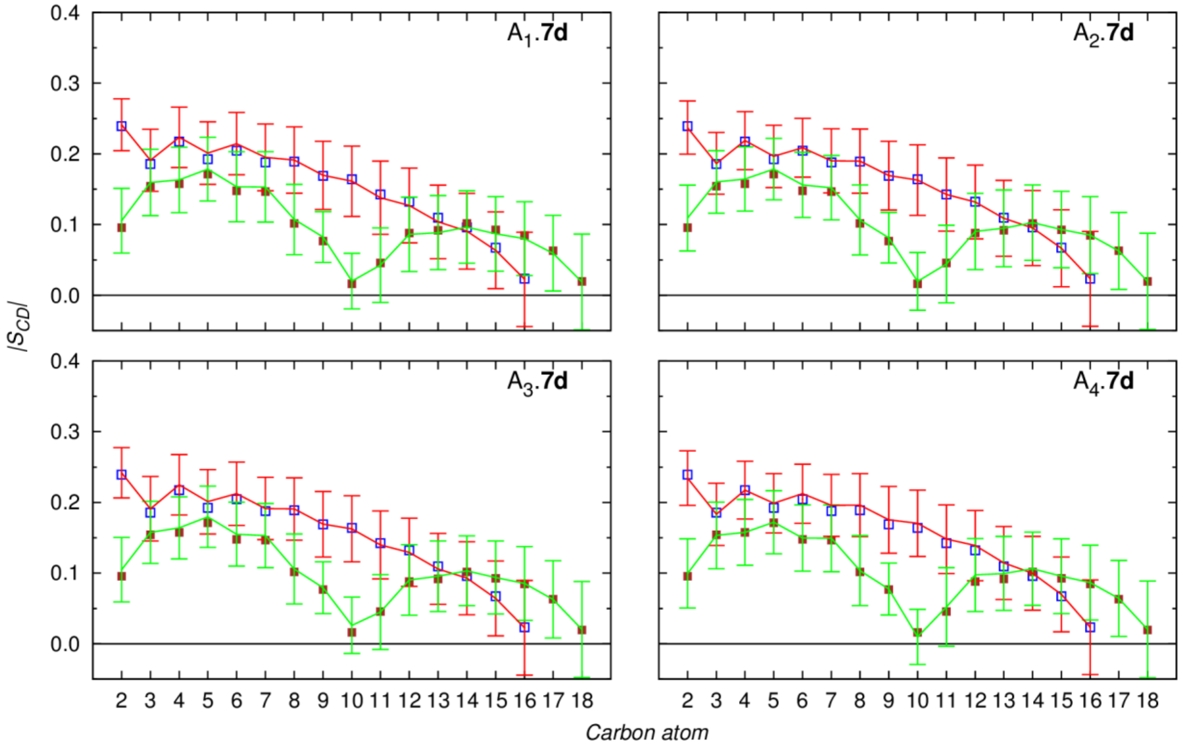

Figure S37. Computed $|S_{CD}|$ for the palmitoyl and oleyl chains for the last 50 ns of MD simulation runs A_1.**7d**-A_4.**7d**. The $|S_{CD}|$ values calculated for the sn-1 chain are shown in red, while the values for the sn-2 chain are shown in green. The error bars associated with these results correspond to the SD. The computed $|S_{CD}|$ values from the free membrane are presented as blue open squares (sn-1 chain), and brown filled squares (sn-2 chain).

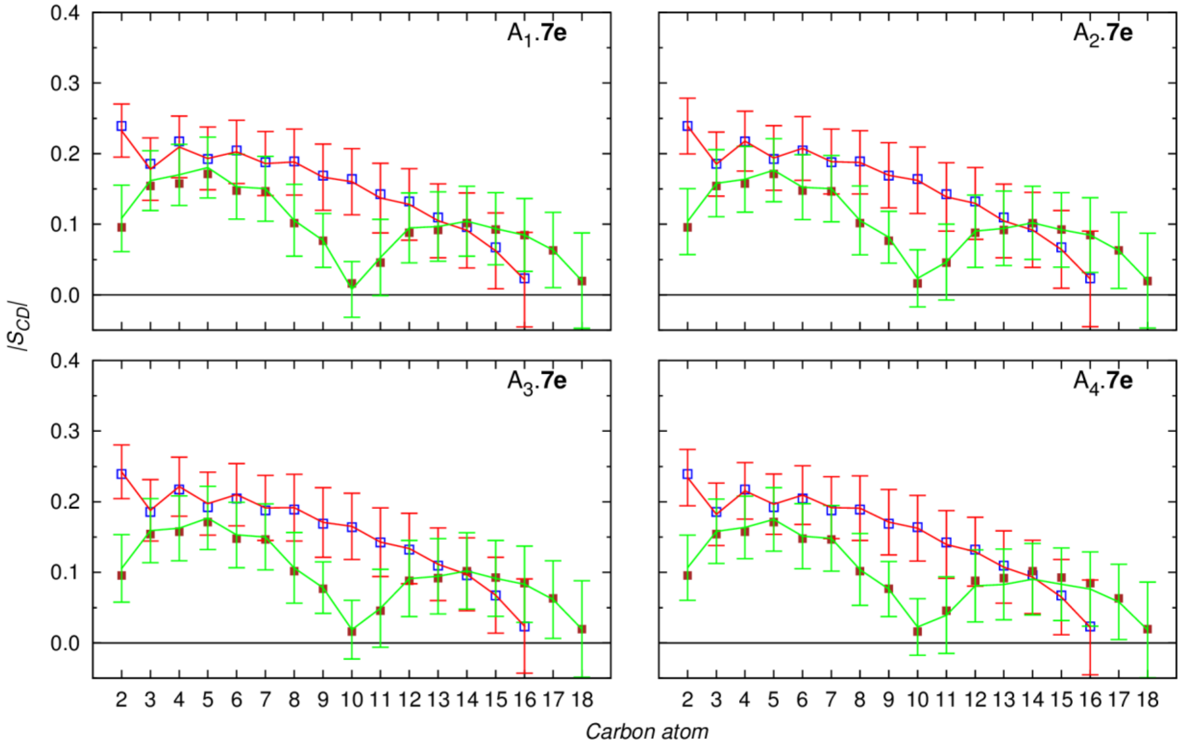

Figure S38. Computed |SCD| for the palmitoyl and oleyl chains for the last 50 ns of MD simulation runs A1.7e-A4.7e. The |SCD| values calculated for the sn-1 chain are shown in red, while the values for the sn-2 chain are shown in green. The error bars associated with these results correspond to the SD. The computed |SCD| values from the free membrane are presented as blue open squares (sn-1 chain), and brown filled squares (sn-2 chain).

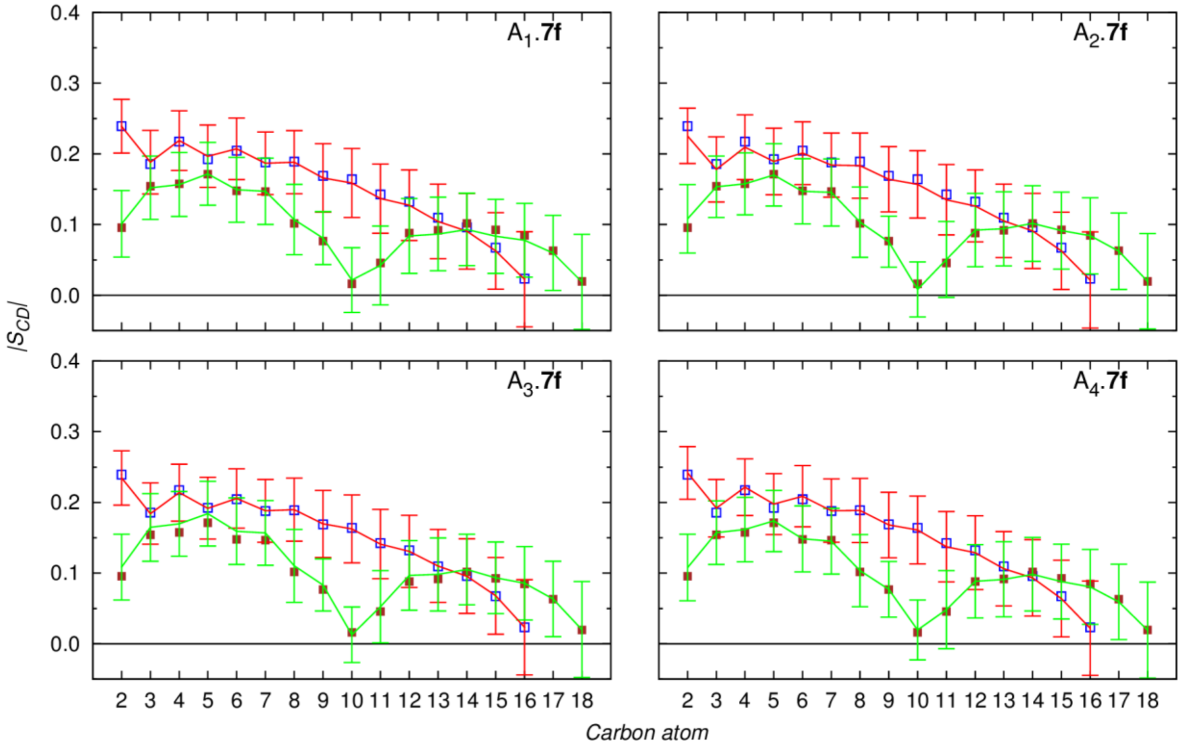

Figure S39. Computed $|S_{CD}|$ for the palmitoyl and oleyl chains for the last 50 ns of MD simulation runs A_1.**7f**-A_4.**7f**. The $|S_{CD}|$ values calculated for the sn-1 chain are shown in red, while the values for the sn-2 chain are shown in green. The error bars associated with these results correspond to the SD. The computed $|S_{CD}|$ values from the free membrane are presented as blue open squares (*sn*-1 chain), and brown filled squares (*sn*-2 chain).

4.3 Detailed Results of the Molecular Dynamics Simulations

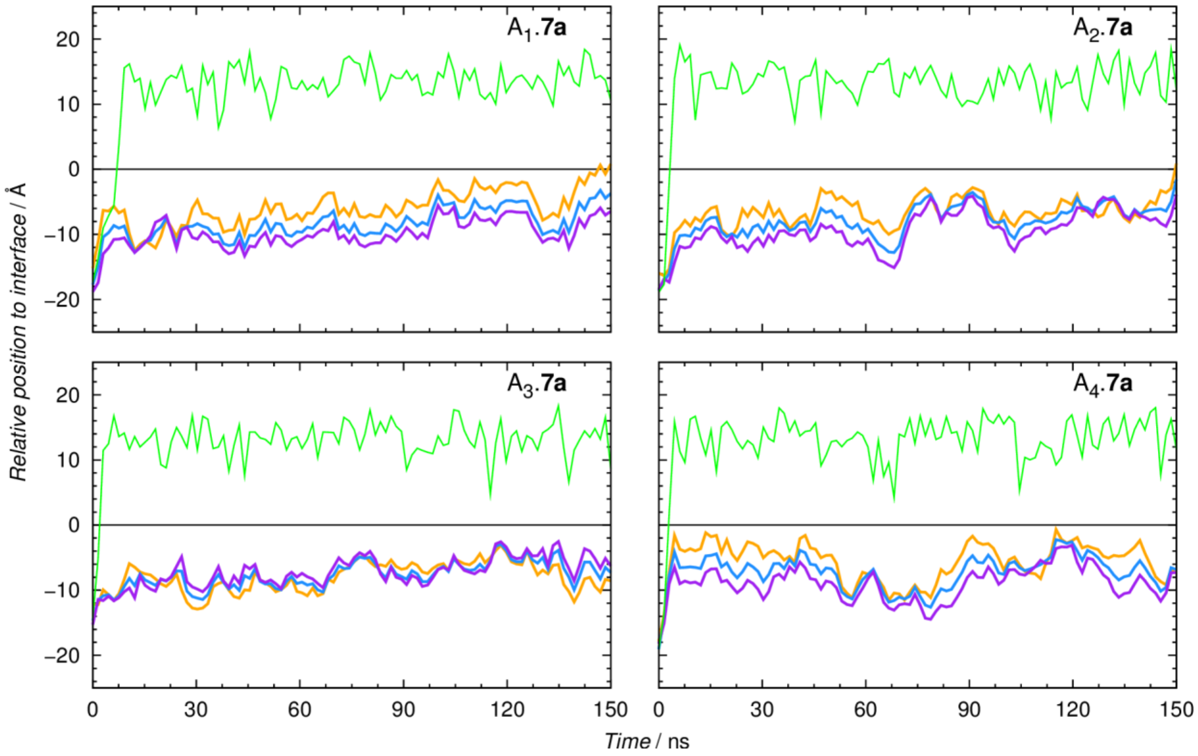

Figure S40. Evolution of $P_{int}\cdots decalin_{COM}$, $P_{int}\cdots N\text{-}H_{COM}$ and $P_{int}\cdots p\text{-}C_{COM}$ distances for MD simulations A_1.**7a**-A_4.**7a**, as well as $P_{int}\cdots Cl^-$ during 150 ns of simulation time. $P_{int}\cdots decalin_{COM}$, $P_{int}\cdots N\text{-}H_{COM}$, $P_{int}\cdots p\text{-}C_{COM}$ and $P_{int}\cdots Cl^-$ are shown in purple, blue, orange and green lines, in this order. The line colouring is in agreement with the sketch given in Fig. 2 of the main text. The water/lipid interface is represented as a black line at z = 0 Å.

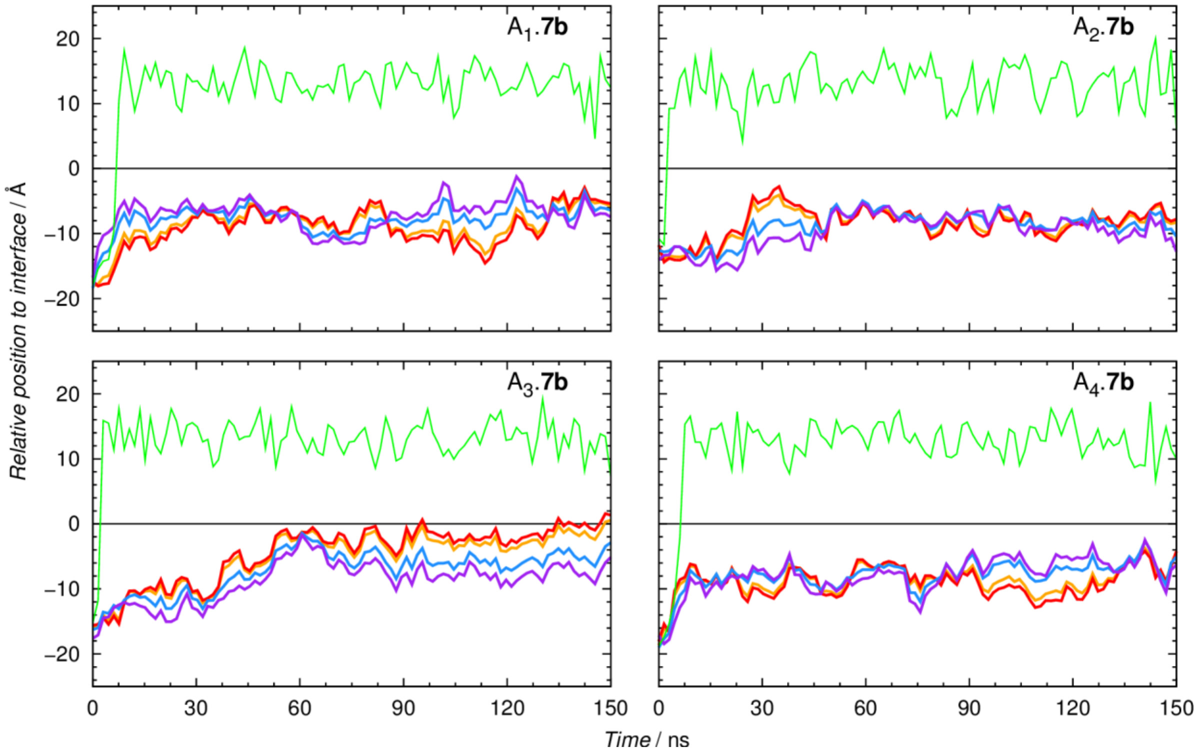

Figure S41. Evolution of $P_{int}\cdots decalin_{COM}$, $P_{int}\cdots N\text{-}H_{COM}$, $P_{int}\cdots p\text{-}C_{COM}$ and $P_{int}\cdots tail_{COM}$ distances for MD simulations A_1.**7b**-A_4.**7b**, as well as $P_{int}\cdots Cl^-$ during 150 ns of simulation time. $P_{int}\cdots decalin_{COM}$, $P_{int}\cdots N\text{-}H_{COM}$, $P_{int}\cdots p\text{-}C_{COM}$, $P_{int}\cdots tail_{COM}$ and $P_{int}\cdots Cl^-$ are shown in purple, blue, orange and red and green lines, in this order. The line colouring is in agreement with the sketch given in Fig. 2 of the main text. The water/lipid interface is represented as a black line at z = 0 Å.

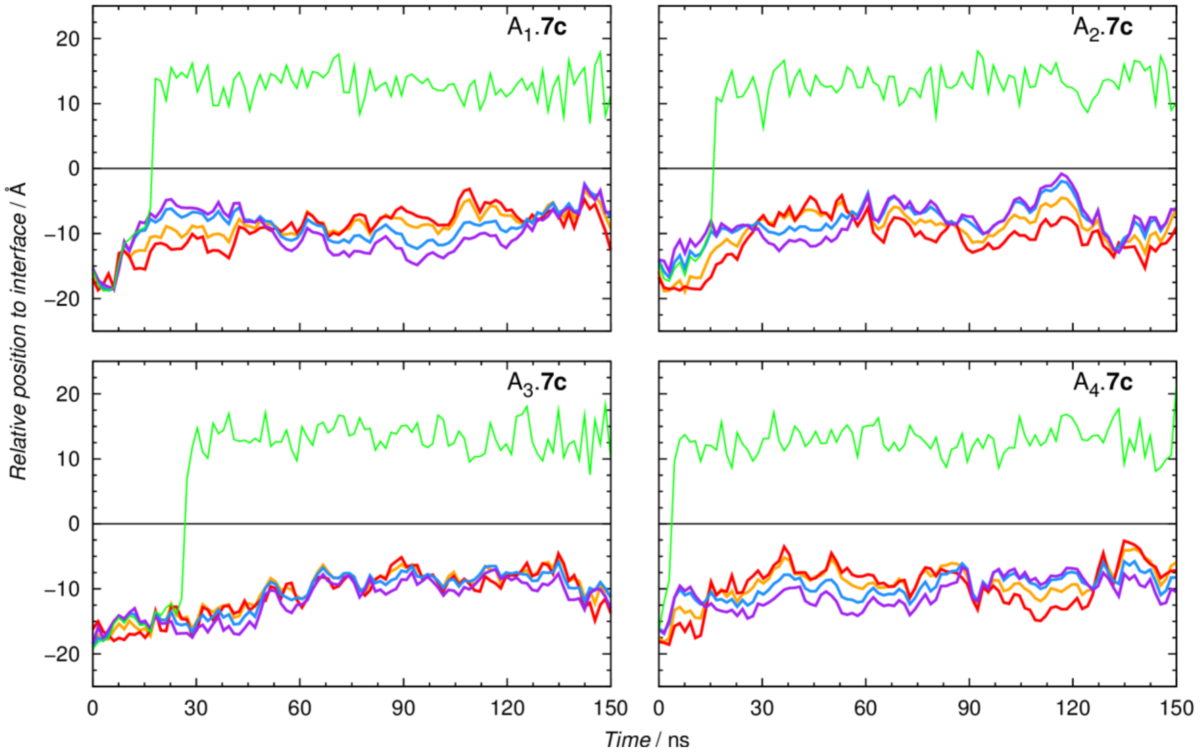

Figure S42. Evolution of P_{int}···decalin$_{COM}$, P_{int}···N-H$_{COM}$, P_{int}···*p*-C$_{COM}$ and P_{int}···tail$_{COM}$ distances for MD simulations A_1.**7c**-A_4.**7c**, as well as P_{int}···Cl^- during 150 ns of simulation time. P_{int}···decalin$_{COM}$, P_{int}···N-H$_{COM}$, P_{int}···*p*-C$_{COM}$, P_{int}···tail$_{COM}$ and P_{int}···Cl^- are shown in purple, blue, orange and red and green lines, in this order. The line colouring is in agreement with the sketch given in Fig. 2 of the main text. The water/lipid interface is represented as a black line at z = 0 Å.

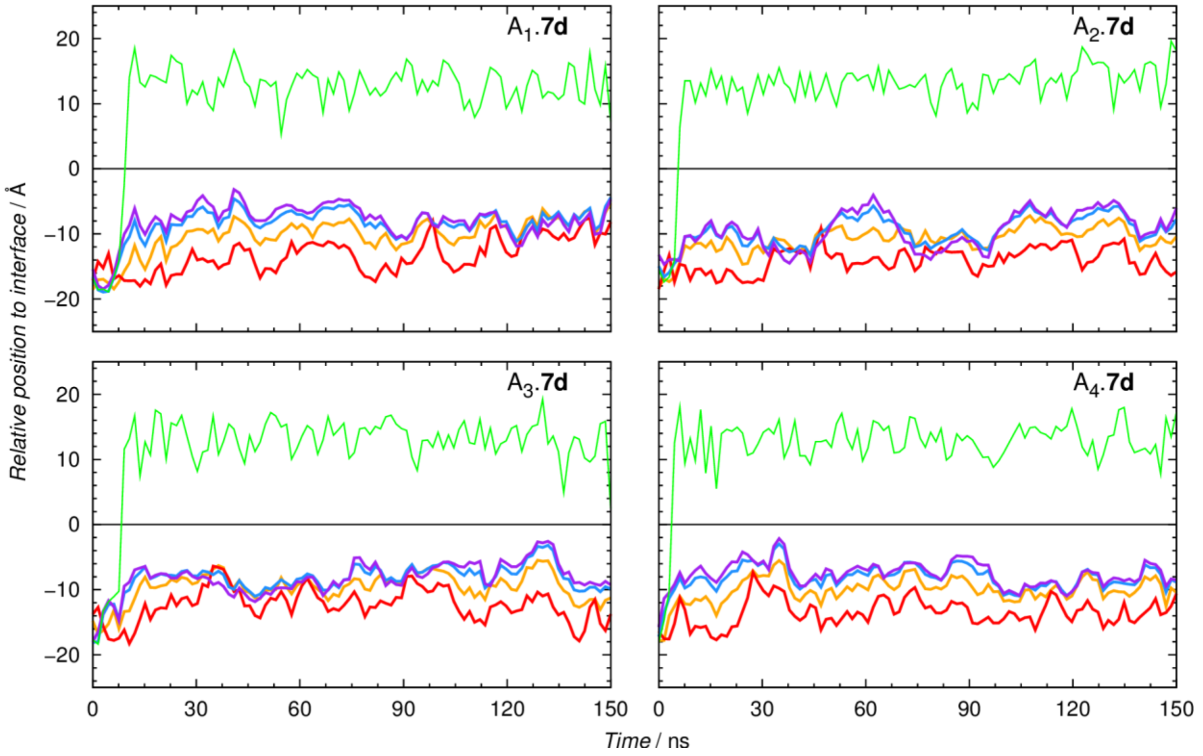

Figure S43. Evolution of P_{int}···decalin$_{COM}$, P_{int}···N-H$_{COM}$, P_{int}···*p*-C$_{COM}$ and P_{int}···tail$_{COM}$ distances for MD simulations A_1.**7d**-A_4.**7d**, as well as P_{int}···Cl^- during 150 ns of simulation time. P_{int}···decalin$_{COM}$, P_{int}···N-H$_{COM}$, P_{int}···*p*-C$_{COM}$, P_{int}···tail$_{COM}$ and P_{int}···Cl^- are shown in purple, blue, orange and red and green lines, in this order. The line colouring is in agreement with the sketch given in Fig. 2 of the main text. The water/lipid interface is represented as a black line at z = 0 Å.

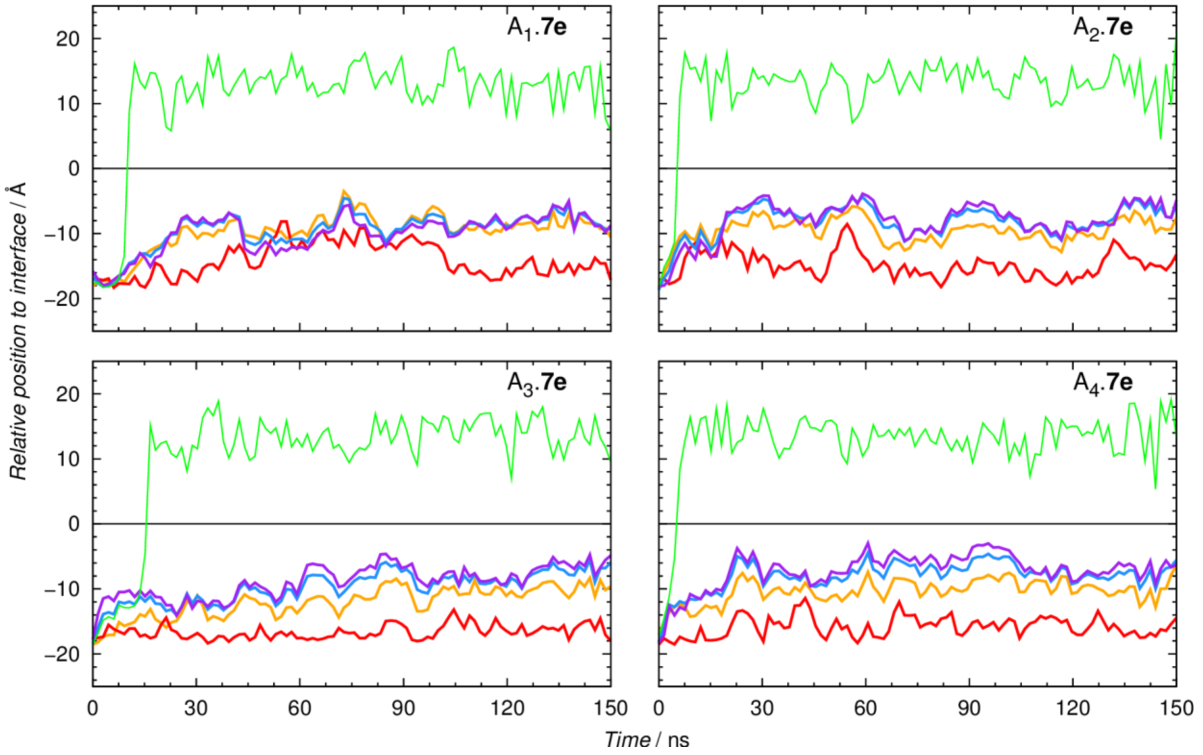

Figure S44. Evolution of P$_{int}$···decalin$_{COM}$, P$_{int}$···N-H$_{COM}$, P$_{int}$···*p*-C$_{COM}$ and P$_{int}$···tail$_{COM}$ distances for MD simulations A$_1$.**7e**-A$_4$.**7e**, as well as P$_{int}$···Cl$^-$ during 150 ns of simulation time. P$_{int}$···decalin$_{COM}$, P$_{int}$···N-H$_{COM}$, P$_{int}$···*p*-C$_{COM}$, P$_{int}$···tail$_{COM}$ and P$_{int}$···Cl$^-$ are shown in purple, blue, orange and red and green lines, in this order. The line colouring is in agreement with the sketch given in Fig. 2 of the main text. The water/lipid interface is represented as a black line at z = 0 Å.

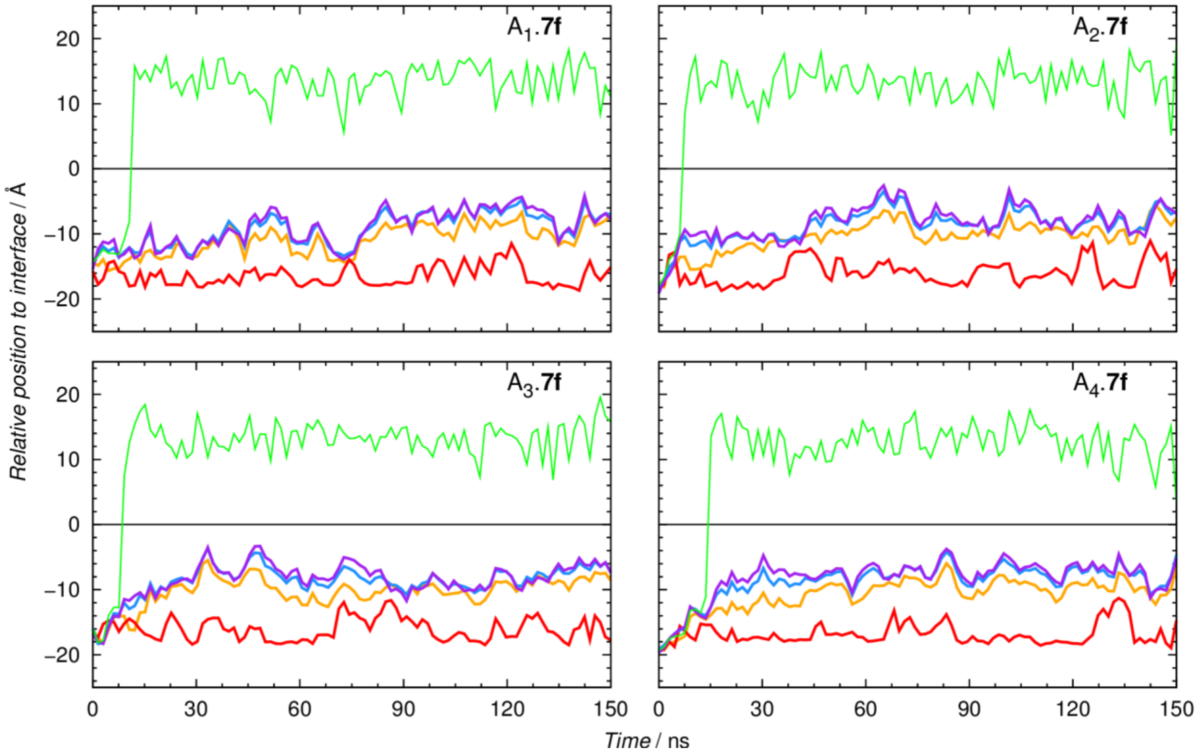

Figure S45. Evolution of P$_{int}$···decalin$_{COM}$, P$_{int}$···N-H$_{COM}$, P$_{int}$···*p*-C$_{COM}$ and P$_{int}$···tail$_{COM}$ distances for MD simulations A$_1$.**7f**-A$_4$.**7f**, as well as P$_{int}$···Cl$^-$ during 150 ns of simulation time. P$_{int}$···decalin$_{COM}$, P$_{int}$···N-H$_{COM}$, P$_{int}$···*p*-C$_{COM}$, P$_{int}$···tail$_{COM}$ and P$_{int}$···Cl$^-$ are shown in purple, blue, orange and red and green lines, in this order. The line colouring is in agreement with the sketch given in Fig. 2 of the main text. The water/lipid interface is represented as a black line at z = 0 Å.

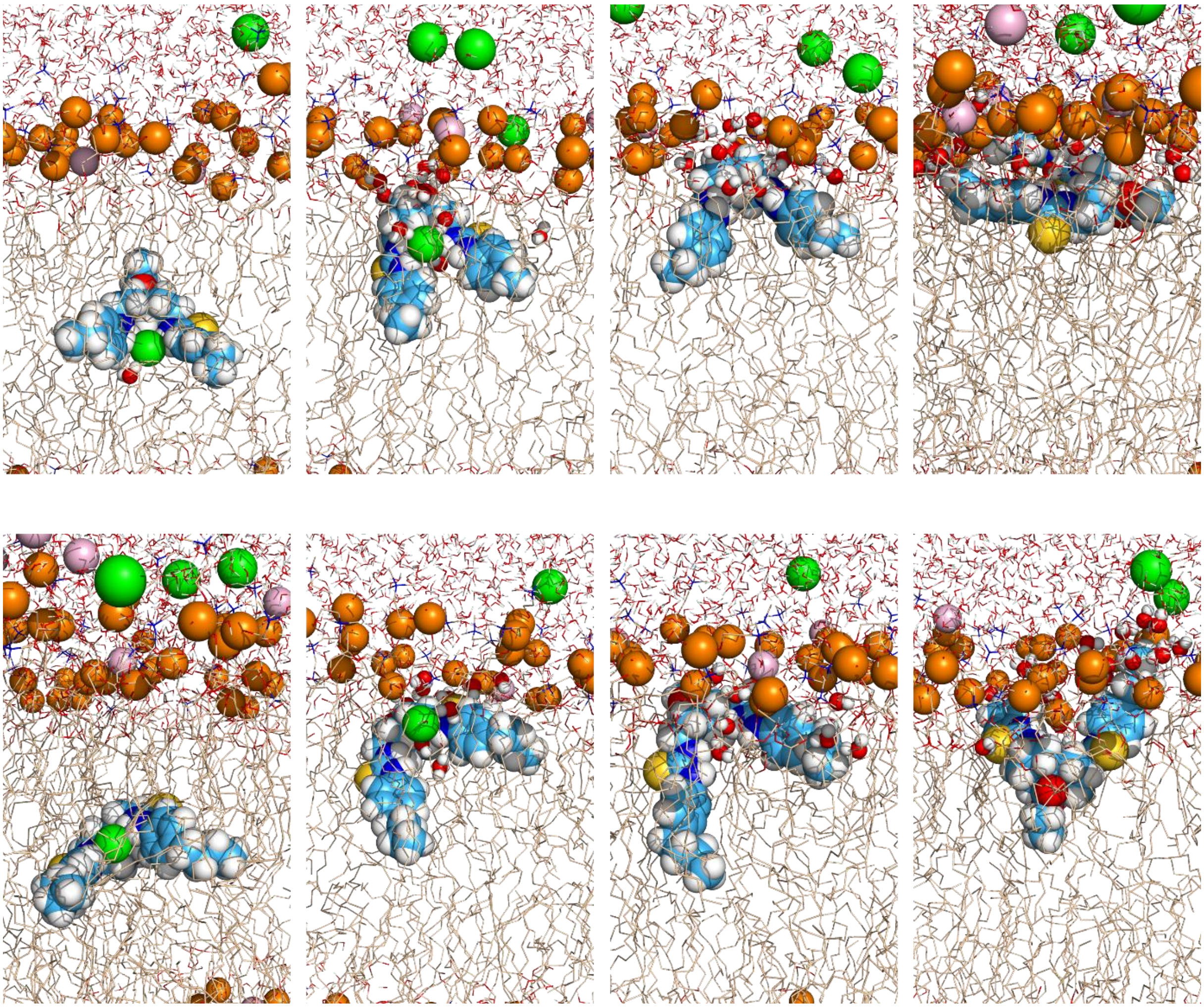

Figure S46. Consecutive snapshots depicting the diffusion of the chloride complex of **7b** and **7c** in simulations A_1.**7b** (top) and A_1.**7c** (bottom). The transporter, the phosphorus atoms and ions are represented in spheres. The hydrogen atoms are shown in white, oxygen atoms in red, nitrogen atoms in blue, sulfur atoms in yellow, phosphorus atoms in orange and carbon atoms in light blue (transporter) or wheat (phospholipids), while the chloride and sodium ions are shown in green and pink, respectively. The chloride decomplexation assisted by water is emphasized with the depiction of water molecules within 3.5 Å from **7b** or **7c** as spheres. The lipids C–H bonds were omitted for clarity.

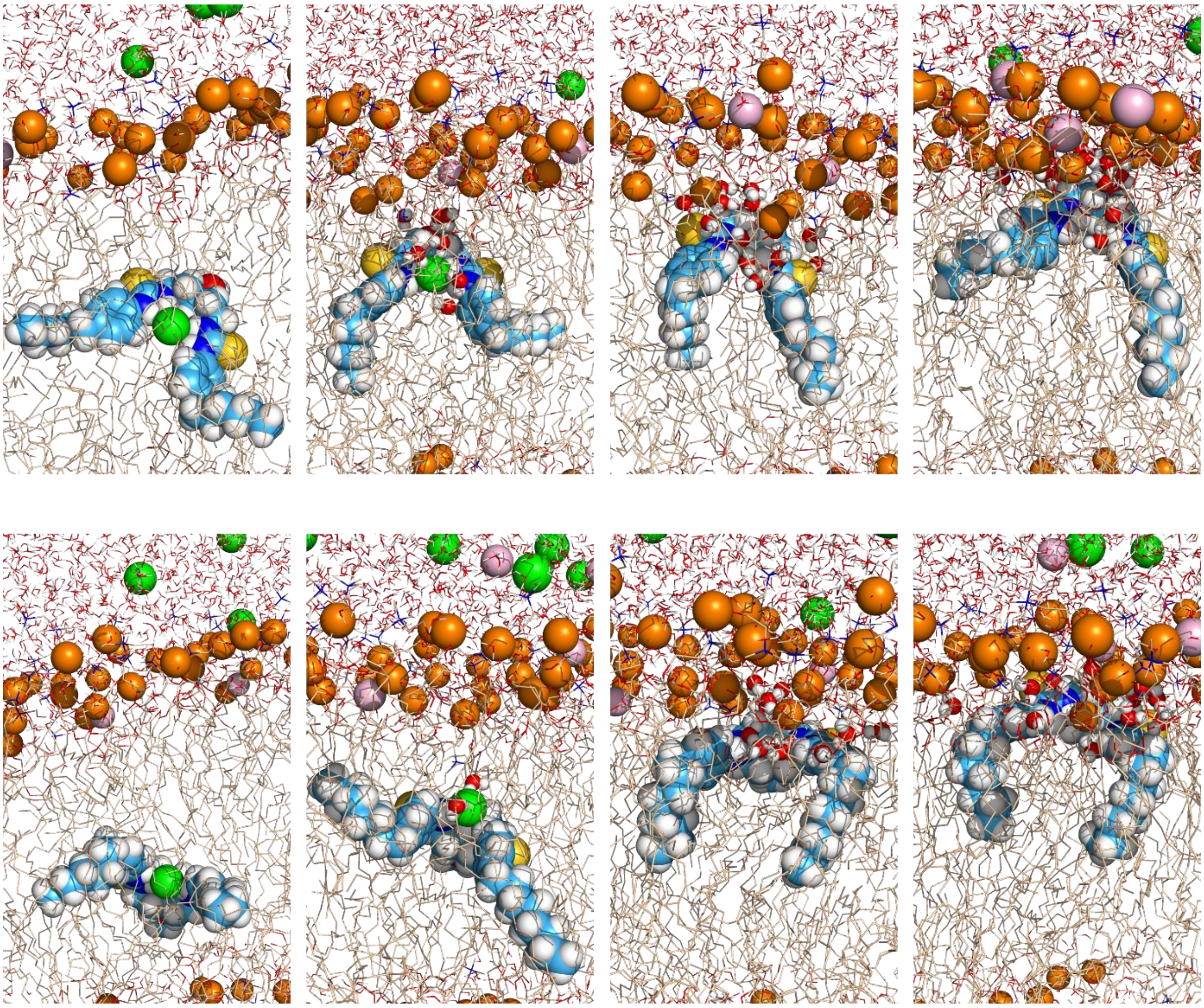

Figure S47. Consecutive snapshots depicting the diffusion of the chloride complex of **7d** and **7e** in simulations A_1.**7d** (top) and A_1.**7e** (bottom). The transporter, the phosphorus atoms and ions are represented in spheres. The hydrogen atoms are shown in white, oxygen atoms in red, nitrogen atoms in blue, sulfur atoms in yellow, phosphorus atoms in orange and carbon atoms in light blue (transporter) or wheat (phospholipids), while the chloride and sodium ions are shown in green and pink, respectively. The chloride decomplexation assisted by water is emphasized with the depiction of water molecules within 3.5 Å from **7d** or **7e** as spheres. The lipids C–H bonds were omitted for clarity.

Table S5. $P_{int}\cdots decalin_{COM}$, $P_{int}\cdots N\text{-}H_{COM}$, $P_{int}\cdots p\text{-}C_{COM}$ and $P_{int}\cdots tail_{COM}$ distances (Å) averaged (Avg) over all replicates for each system with the corresponding standard deviations (SD).[a,b]

System ID	$P_{int}\cdots decalin_{COM}$	$P_{int}\cdots N\text{-}H_{COM}$	$P_{int}\cdots p\text{-}C_{COM}$	$P_{int}\cdots tail_{COM}$
	Avg ± SD	Avg ± SD	Avg ± SD	Avg ± SD
A.**7a**	7.17 ± 2.36	6.27 ± 1.92	2.12 ± 3.04	NA[c)]
A.**7b**	7.17 ± 2.20	6.80 ± 1.79	5.40 ± 1.54	6.86 ± 3.99
A.**7c**	8.51 ± 2.79	7.87 ± 2.26	7.86 ± 2.10	9.00 ± 3.08
A.**7d**	7.73 ± 2.07	8.04 ± 1.88	8.01 ± 1.99	12.80 ± 2.40
A.**7e**	7.55 ± 1.72	7.94 ± 1.49	8.52 ± 1.83	15.60 ± 1.63
A.**7f**	7.37 ± 1.93	7.63 ± 1.66	7.98 ± 1.66	16.19 ± 2.21
A'.**7a**	6.07 ± 2.26	4.85 ± 2.52	2.12 ± 3.04	NA[c)]
A'.**7b**	6.34 ± 1.77	5.70 ± 1.38	5.40 ± 1.54	5.32 ± 1.82
A'.**7c**	9.42 ± 1.64	8.30 ± 1.43	7.86 ± 2.10	7.97 ± 3.31
A'.**7d**	6.66 ± 2.93	6.66 ± 2.49	8.01 ± 1.99	12.01 ± 1.71
A'.**7e**	8.07 ± 1.70	8.47 ± 1.59	8.52 ± 1.83	13.65 ± 2.53
A'.**7f**	6.81 ± 2.74	7.42 ± 2.27	7.98 ± 1.66	14.07 ± 2.03
B.**7a**	7.05 ± 2.13	5.27 ± 2.07	2.12 ± 3.04	NA[c)]
B.**7b**	8.60 ± 1.71	8.21 ± 1.50	5.40 ± 1.54	8.22 ± 2.64
B.**7c**	8.15 ± 2.01	8.40 ± 2.01	7.86 ± 2.10	10.51 ± 3.46
B.**7d**	7.53 ± 1.66	7.78 ± 1.64	8.01 ± 1.99	12.77 ± 2.17
B.**7e**	6.81 ± 2.36	7.50 ± 2.00	8.52 ± 1.83	14.31 ± 2.19
B.**7f**	9.22 ± 2.24	8.59 ± 1.79	7.98 ± 1.66	15.28 ± 2.71

[a)] N =20000, 5000 and 10000 for systems derived from setups A, A' and B, respectively. [b)] Values for each independent replicate can be found in Table S6 and Table S7; [c)] NA – Not Applicable.

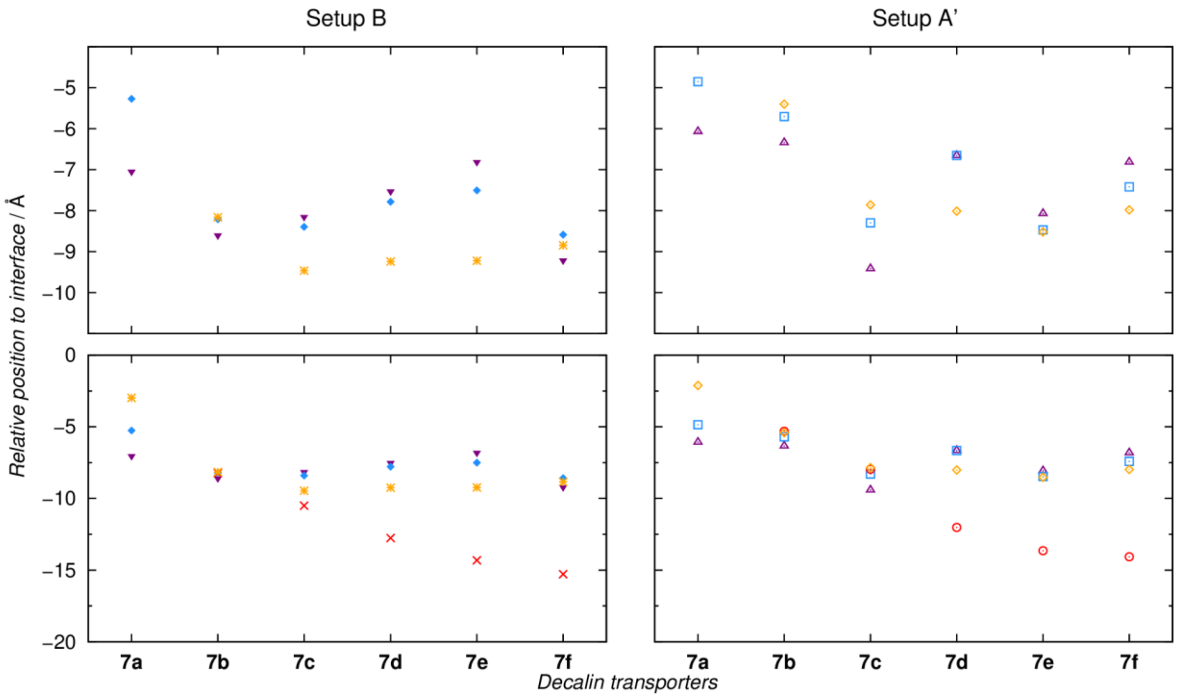

Figure S48. Average positions of decalin$_{COM}$, N-H$_{COM}$, tail$_{COM}$ and *p*-C$_{COM}$ thiourea reference points relatively to the closest interface. Each point was calculated averaging 100 ns for setup B simulations (decalin$_{COM}$ – ▼; N-H$_{COM}$ – ◆; *p*-C$_{COM}$ – ✳; and tail$_{COM}$ – ×), and 50 ns for setup A’ simulations (decalin$_{COM}$ – △; N-H$_{COM}$ – □; *p*-C$_{COM}$ – ◊; and tail$_{COM}$ – O).

Table S6. $P_{int}\cdots decalin_{COM}$, $P_{int}\cdots N\text{-}H_{COM}$, $P_{int}\cdots p\text{-}C_{COM}$ and $P_{int}\cdots tail_{COM}$ distances (Å) averaged (Avg) for the last 50 ns of simulations A_1.**7a**-A_4.**7f** with the corresponding standard deviations (SD).[a)]

Simulation ID	$P_{int}\cdots decalin_{COM}$	$P_{int}\cdots N\text{-}H_{COM}$	$P_{int}\cdots p\text{-}C_{COM}$	$P_{int}\cdots tail_{COM}$
	Avg ± SD	Avg ± SD		Avg ± SD
A_1.**7a**	8.11 ± 1.87	6.27 ± 2.01	3.32 ± 2.27	NA[b)]
A_2.**7a**	7.89 ± 2.21	6.77 ± 1.78	6.05 ± 1.87	NA[b)]
A_3.**7a**	5.35 ± 1.82	6.04 ± 1.75	6.79 ± 2.40	NA[b)]
A_4.**7a**	7.32 ± 2.39	6.02 ± 2.03	4.95 ± 2.24	NA[b)]
A_1.**7b**	5.73 ± 2.03	6.59 ± 1.73	8.06 ± 2.85	8.63 ± 3.46
A_2.**7b**	9.21 ± 1.59	8.39 ± 1.31	8.23 ± 1.52	8.22 ± 1.80
A_3.**7b**	7.71 ± 1.34	5.59 ± 1.28	2.45 ± 1.40	1.42 ± 1.52
A_4.**7b**	6.02 ± 1.75	6.64 ± 1.55	8.49 ± 2.15	9.19 ± 2.50
A_1.**7c**	8.82 ± 3.05	7.59 ± 2.36	6.75 ± 1.68	6.79 ± 1.99
A_2.**7c**	6.66 ± 3.31	7.07 ± 2.96	8.75 ± 2.56	10.79 ± 2.14
A_3.**7c**	9.75 ± 1.70	8.56 ± 1.58	8.30 ± 1.78	8.92 ± 2.35
A_4.**7c**	8.80 ± 1.69	8.26 ± 1.51	8.29 ± 2.56	9.50 ± 3.94
A_1.**7d**	8.42 ± 1.76	8.02 ± 1.60	8.68 ± 1.87	11.29 ± 2.69
A_2.**7d**	7.23 ± 1.82	7.78 ± 1.67	9.53 ± 1.66	13.23 ± 2.01
A_3.**7d**	6.55 ± 2.23	7.33 ± 2.25	9.47 ± 2.36	13.15 ± 2.45
A_4.**7d**	8.74 ± 1.62	9.02 ± 1.49	10.10 ± 1.48	13.52 ± 1.65
A_1.**7e**	8.08 ± 1.61	8.08 ± 1.34	8.78 ± 1.21	15.38 ± 1.43
A_2.**7e**	7.70 ± 1.97	7.94 ± 1.80	9.62 ± 1.65	15.30 ± 2.00
A_3.**7e**	7.40 ± 1.57	8.04 ± 1.33	10.09 ± 1.12	15.89 ± 1.43
A_4.**7e**	7.03 ± 1.53	7.72 ± 1.41	9.80 ± 1.56	15.82 ± 1.51
A_1.**7f**	6.77 ± 1.85	7.20 ± 1.68	9.13 ± 1.68	15.87 ± 2.20
A_2.**7f**	7.43 ± 2.06	7.64 ± 1.76	9.15 ± 1.66	15.72 ± 2.37
A_3.**7f**	8.03 ± 1.81	8.08 ± 1.56	9.42 ± 1.48	16.53 ± 1.68
A_4.**7f**	7.23 ± 1.79	7.58 ± 1.50	9.59 ± 1.32	16.65 ± 2.36

[a)] N = 5000; [b)] NA – Not Applicable.

Table S7. $P_{int}\cdots decalin_{COM}$, $P_{int}\cdots N\text{-}H_{COM}$, $P_{int}\cdots p\text{-}C_{COM}$ and $P_{int}\cdots tail_{COM}$ distances (Å) averaged (Avg) for the last 50 ns of simulations B_1.**7a**-B_2.**7f** with the corresponding standard deviations (SD).[a)]

Simulation ID	$P_{int}\cdots decalin_{COM}$	$P_{int}\cdots N\text{-}H_{COM}$	$P_{int}\cdots p\text{-}C_{COM}$	$P_{int}\cdots tail_{COM}$
	Avg ± SD	Avg ± SD	Avg ± SD	Avg ± SD
B_1.**7a**	7.07 ± 2.19	6.05 ± 1.81	5.03 ± 2.97	NA[b)]
B_2.**7a**	7.02 ± 2.07	4.49 ± 2.03	0.93 ± 2.14	NA[b)]
B_1.**7b**	9.11 ± 1.66	7.79 ± 1.43	7.14 ± 1.60	7.01 ± 1.85
B_2.**7b**	8.09 ± 1.60	8.63 ± 1.45	9.18 ± 2.31	9.43 ± 2.75
B_1.**7c**	7.94 ± 2.06	9.31 ± 1.97	11.60 ± 2.08	13.20 ± 2.39
B_2.**7c**	8.36 ± 1.93	7.48 ± 1.57	7.33 ± 1.52	7.82 ± 1.93
B_1.**7d**	7.37 ± 1.68	7.91 ± 1.78	9.82 ± 2.06	13.69 ± 2.10
B_2.**7d**	7.69 ± 1.64	7.66 ± 1.47	8.66 ± 1.43	11.85 ± 1.81
B_1.**7e**	7.23 ± 2.11	8.01 ± 1.91	10.31 ± 1.87	15.25 ± 2.14
B_2.**7e**	6.40 ± 2.52	7.00 ± 1.96	8.14 ± 1.53	13.37 ± 1.81
B_1.**7f**	8.02 ± 1.62	8.04 ± 1.52	9.13 ± 1.56	16.53 ± 1.93
B_2.**7f**	10.41 ± 2.14	9.14 ± 1.87	8.57 ± 1.72	14.03 ± 2.80

[a)] N = 5000; [b)] NA – Not Applicable.

4.4 Interaction energies between transporters 7a-f and POPC bilayer

The interaction energies between the POPC membrane model and the thiourea molecules were energetically evaluated through equation 1:

$$\Delta E_{TOTAL} = \Delta E_{ELEC} + \Delta E_{vdW} + \Delta E_{INT} \quad (1)$$

ΔE_{ELEC} and ΔE_{vdW} correspond to the non-bonded electrostatic and van der Waals energy terms, while ΔE_{INT} corresponds to the sum of bond, angle, and dihedral energies. The three individual molecular mechanics energy components are given by equations 2-4:

$$\Delta E_{ELEC} = E_{ELEC}(System) - (E_{ELEC}(POPC) + E_{ELEC}(\boldsymbol{7m})) \quad (2)$$

$$\Delta E_{vdW} = E_{vdW}(System) - (E_{vdW}(POPC) + E_{vdW}(\boldsymbol{7m})) \quad (3)$$

$$\Delta E_{INT} = E_{INT}(System) - (E_{INT}(POPC) + E_{INT}(\boldsymbol{7m})) \quad (4)$$

Here *System* represents the transporter and the phospholipid membrane discarding the water molecules and all chloride anions and sodium counter-ions. *POPC* stands for the 128 phospholipid molecules and ***7m*** (*m* = **a**, **b**, **c**, **d**, **e** or **f**) for the isolated transporter. These three energies were estimated with snapshots extracted every 100 ps from the last 50 ns of each MD simulation carried out with setup A.

The bonded term (ΔE_{INT}) amounts to zero, given that the individual terms INT_{System}, INT_{POPC} and INT_{7m} were calculated using the same MD simulation. In these conditions, through equation 1, the contribution of both non-bonded energy terms for the intermolecular interactions between the phospholipids and decaline transporters is evaluated. The average values of ΔE_{TOTAL}, ΔE_{ELEC} and ΔE_{vdW} for 200 ns of sampling, given in Table S8 for each system and in Table S9 for each replicate, and visualized in Figure S49.

Table S8. Average molecular mechanics energy terms (kcal/mol) with the corresponding standard deviations.[a)]

System ID	ΔE_{TOTAL}	ΔE_{ELEC}	ΔE_{vdW}
A.**7a**	-95.64 ± 15.90	-33.95 ± 12.45	-61.69 ± 6.45
A.**7b**	-108.21 ± 16.56	-38.84 ± 15.65	-69.37 ± 6.73
A.**7c**	-112.94 ± 14.59	-33.41 ± 11.34	-79.53 ± 6.51
A.**7d**	-113.26 ± 23.45	-29.06 ± 14.24	-84.20 ± 11.58
A.**7e**	-134.24 ± 14.78	-36.87 ± 11.28	-97.37 ± 6.64
A.**7f**	-127.66 ± 14.02	-25.10 ± 11.25	-102.55 ± 7.17

[a)] *N* = 2000 (100 ps interval between snapshots)

Table S9. Average molecular mechanics energy terms (kcal/mol) with the corresponding standard deviations for each MD simulation.[a)]

Simulation ID	ΔE_{TOTAL}	ΔE_{ELEC}	ΔE_{vdW}
	Avg ± SD	Avg ± SD	Avg ± SD
A_1.**7a**	-108.02 ± 10.65	-44.65 ± 9.20	-63.38 ± 4.87
A_2.**7a**	-84.79 ± 17.83	-27.82 ± 14.48	-56.97 ± 5.97
A_3.**7a**	-101.42 ± 7.41	-34.83 ± 6.73	-66.59 ± 4.31
A_4.**7a**	-88.35 ± 12.98	-28.52 ± 9.94	-59.83 ± 6.00
A_1.**7b**	-104.01 ± 14.15	-32.61 ± 13.56	-71.40 ± 4.36
A_2.**7b**	-115.38 ± 14.14	-40.19 ± 11.28	-75.19 ± 5.31
A_3.**7b**	-113.62 ± 16.87	-50.82 ± 15.04	-62.81 ± 5.50
A_4.**7b**	-99.82 ± 15.67	-31.73 ± 14.47	-68.09 ± 4.60
A_1.**7c**	-107.70 ± 14.26	-32.90 ± 11.43	-74.80 ± 5.13
A_2.**7c**	-118.93 ± 8.38	-34.81 ± 6.23	-84.12 ± 5.25
A_3.**7c**	-111.07 ± 13.60	-29.66 ± 9.13	-81.41 ± 6.20
A_4.**7c**	-114.07 ± 18.07	-36.28 ± 15.42	-77.79 ± 5.24
A_1.**7d**	-80.51 ± 7.60	-13.85 ± 5.85	-66.67 ± 5.49
A_2.**7d**	-124.48 ± 11.32	-32.30 ± 8.41	-92.18 ± 5.49
A_3.**7d**	-109.17 ± 8.84	-22.78 ± 6.92	-86.40 ± 4.90
A_4.**7d**	-138.89 ± 8.03	-47.32 ± 6.65	-91.57 ± 4.76
A_1.**7e**	-149.52 ± 8.35	-47.84 ± 6.72	-101.68 ± 4.79
A_2.**7e**	-130.64 ± 10.62	-32.55 ± 8.89	-98.09 ± 5.30
A_3.**7e**	-128.90 ± 17.21	-36.53 ± 12.62	-92.37 ± 6.86
A_4.**7e**	-127.90 ± 9.01	-30.56 ± 6.87	-97.34 ± 5.85
A_1.**7f**	-129.55 ± 14.37	-29.23 ± 12.08	-100.33 ± 5.54
A_2.**7f**	-119.45 ± 10.41	-18.52 ± 6.20	-100.93 ± 7.86
A_3.**7f**	-131.60 ± 14.20	-24.40 ± 12.84	-107.20 ± 5.09
A_4.**7f**	-130.03 ± 13.36	-28.27 ± 9.35	-101.76 ± 7.62

[a)] N = 500 (100 ps interval between snapshots)

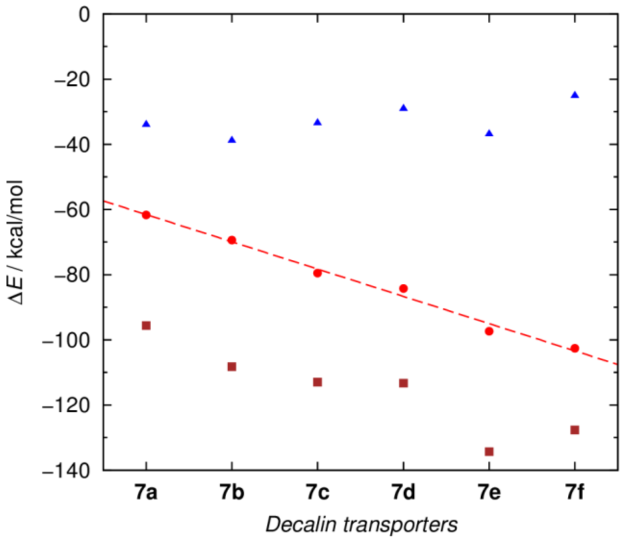

Figure S49. Average ΔE_{TOTAL} (■), ΔE_{ELEC} (▲), and ΔE_{vdW} (●) energies. Each point was calculated averaging the 200 ns of sampling for setup A simulations. A linear fit of the ΔE_{vdW} energies is shown as red dashed line ($R^2 = 0.99$) with a slope of -8 kcal/mol per ethylene unit.

The ΔE_{ELEC} values are almost independent of the transporter. This term also includes the hydrogen bonding interactions between the phospholipid heads and N-H binding sites, which were not found to differ over the range of transporters (Table S8) in agreement with the similar P_{int}···decalin$_{COM}$ and P_{int}···N-H_{COM} distances reported in Fig. 4 of the main text. In contrast, the ΔE_{vdW} term is the major contribution for the stabilisation of the transporters inserted into the phospholipid bilayers. As expected, a linear trend between the number of carbon atoms of alkyl chain substituent (and thus the total size of the molecule) and the ΔE_{vdW} was found, with the most negative stabilizing energy between **7f**, bearing the longest alkyl substituents, and the phospholipids.

4.5 Supplementary MD Movie Caption

Movie S1.Passive diffusion of the chloride complex of 7d in simulation A_1.7d. This movie shows the passive diffusion of the chloride complex of **7d** from the membrane core towards the water interface (simulation A_1.**7d**, between 6-10 ns of MD simulation time). This movie also features the chloride release below the interface assisted by water molecules coming from the water phase. The chloride decomplexation assisted by water molecules is emphasized with the depiction of water molecules within 3.5 Å from the anion. All atoms are presented in spheres apart from those of phospholipids, which are drawn in stick fashion. The hydrogen atoms are in white, oxygen atoms in red, nitrogen atoms in blue, sulfur atoms in yellow, phosphorus atoms in orange, sodium counter-ions in pink, and carbon atoms in light blue (transporter) or wheat (phospholipids). The coordinated chloride is shown in light green, while the remaining aqueous phase chloride anions are shown in dark green. The lipid C–H bonds are omitted for clarity.

5. References

1 S. Hussain, P. R. Brotherhood, L. W. Judd and A. P. Davis, *J. Am. Chem. Soc.*, **2011**, *133*, 1614–1617.

2 H. Munch, J. S. Hansen, M. Pittelkow, J. B. Christensen and U. Boas, *Tetrahedron Lett.*, **2008**, *49*, 3117–3119.

3 J. P. Clare, A. J. Ayling, J. B. Joos, A. L. Sisson, G. Magro, M. N. Pérez-Payán, T. N. Lambert, R. Shukla, B. D. Smith and A. P. Davis, *J. Am. Chem. Soc.*, **2005**, *127*, 10739–10746.

4 E. P. Kyba, R. C. Helgeson, K. Madan, G. W. Gokel, T. L. Tarnowski, S. S. Moore and D. J. Cram, *J. Am. Chem. Soc.*, **1977**, *99*, 2564–2571.

5 H. Valkenier, L. W. Judd, H. Li, S. Hussain, D. N. Sheppard and A. P. Davis, *J. Am. Chem. Soc.*, **2014**, *136*, 12507–12512.

6 H. Valkenier, C. J. E. Haynes, J. Herniman, P. A. Gale and A. P. Davis, *Chem. Sci.*, **2014**, *5*, 1128–1134.

7 (a) D. A. Case, V. Babin, J. T. Berryman, R. M. Betz, Q. Cai, D. S. Cerutti, I. T. E. Cheatham, T. A. Darden, R. E. Duke, H. Gohlke, A. W. Goetz, S. Gusarov, N. Homeyer, P. Janowski, J. Kaus, I. Kolossváry, A. Kovalenko, T. S. Lee, S. LeGrand, T. Luchko, R. Luo, B. Madej, K. M. Merz, F. Paesani, D. R. Roe, A. Roitberg, C. Sagui, R. Salomon-Ferrer, G. Seabra, C. L. Simmerling, W. Smith, J. Swails, R. C. Walker, J. Wang, R. M. Wolf, X. Wu and P. A. Kollman, *AMBER 14*, (**2014**) University of California, San Francisco; (b) A. W. Gotz, M. J. Williamson, D. Xu, D. Poole, S. Le Grand and R. C. Walker, *J. Chem. Theory Comput.*, **2012**, *8*, 1542–1555; (c) S. Le Grand, A. W. Götz and R. C. Walker, *Comput. Phys. Commun.*, **2013**, *184*, 374–380; (d) R. Salomon-Ferrer, A. W. Gotz, D. Poole, S. Le Grand and R. C. Walker, *J. Chem. Theory Comput.*, **2013**, *9*, 3878–3888.

8 A. A. Skjevik, B. D. Madej, R. C. Walker and K. Teigen, *J. Phys. Chem. B*, **2012**, *116*, 11124–11136.

9 (a) J. Wang, R. M. Wolf, J. W. Caldwell, P. A. Kollman and D. A. Case, *J. Comput. Chem.*, **2004**, *25*, 1157–1174; (b) J. Wang, R. M. Wolf, J. W. Caldwell, P. A. Kollman and D. A. Case, *J. Comput. Chem.*, **2005**, *26*, 114–114.

10 C. I. Bayly, P. Cieplak, W. D. Cornell and P. A. Kollman, *J. Phys. Chem.*, **1993**, *97*, 10269–10280.

11 D. J. Chadwick and J. D. Dunitz, *J Chem Soc Perk T 2*, **1979**, 276–284.

12 M. J. Frisch, G. W. Trucks, H. B. Schlegel, G. E. Scuseria, M. A. Robb, J. R. Cheeseman, G. Scalmani, V. Barone, B. Mennucci, G. A. Petersson, H. Nakatsuji, M. Caricato, X. Li, H. P. Hratchian, A. F. Izmaylov, J. Bloino, G. Zheng, J. L. Sonnenberg, M. Hada, M. Ehara, K. Toyota, R. Fukuda, J. Hasegawa, M. Ishida, T. Nakajima, Y. Honda, O. Kitao, H. Nakai, T. Vreven, J. J. A. Montgomery, J. E. Peralta, F. Ogliaro, M. Bearpark, J. J. Heyd, E. Brothers, K. N. Kudin, V. N. Staroverov, R. Kobayashi, J. Normand, K. Raghavachari, A. Rendell, J. C. Burant, S. S. Iyengar, J. Tomasi, M. Cossi, N. Rega, J. M. Millam, M. Klene, J. E. Knox, J. B. Cross, V. Bakken, C. Adamo, J. Jaramillo, R. Gomperts, R. E. Stratmann, O. Yazyev, A. J. Austin, R. Cammi, C. Pomelli, J. W. Ochterski, R. L. Martin, K. Morokuma, V. G. Zakrzewski, G. A. Voth, P. Salvador, J. J. Dannenberg, S. Dapprich, A. D. Daniels, Ö. Farkas, J. B. Foresman, J. V. Ortiz, J. Cioslowski and D. J. Fox, *Gaussian 09 Revision A1*, (**2009**) Gaussian, Inc., Pittsburgh PA.

13 A. K. Rappe and C. J. R. Casewit, *Molecular Mechanics Across Chemistry*, 1st edn., Univ Science Books **1997**.

14 E. F. Pettersen, T. D. Goddard, C. C. Huang, G. S. Couch, D. M. Greenblatt, E. C. Meng and T. E. Ferrin, *J. Comput. Chem.*, **2004**, *25*, 1605–1612.

15 I. S. Joung and T. E. Cheatham, 3rd, *J. Phys. Chem. B*, **2008**, *112*, 9020–9041.

16 C. J. Dickson, B. D. Madej, A. A. Skjevik, R. M. Betz, K. Teigen, I. R. Gould and R. C. Walker, *J. Chem. Theory Comput.*, **2014**, *10*, 865–879.

17 L. Martinez, R. Andrade, E. G. Birgin and J. M. Martinez, *J. Comput. Chem.*, **2009**, *30*, 2157–2164.

18 T. Darden, D. York and L. Pedersen, *J. Chem. Phys.*, **1993**, *98*, 10089.

19 R. J. Loncharich, B. R. Brooks and R. W. Pastor, *Biopolymers*, **1992**, *32*, 523–535.

20 H. J. C. Berendsen, J. P. M. Postma, W. F. Vangunsteren, A. Dinola and J. R. Haak, *J. Chem. Phys.*, **1984**, *81*, 3684–3690.

21 J.-P. Ryckaert, G. Ciccotti and H. J. C. Berendsen, *J. Comput. Phys.*, **1977**, *23*, 327–341.
